# Sulfated mannan of diatoms selects host-specific microbiota in the sunlit ocean

**DOI:** 10.1186/s40168-026-02379-9

**Published:** 2026-03-26

**Authors:** J. Krull, C. Sidhu, V. Solanki, M. Bligh, L. Rößler, R. K. Singh, G. Huang, C. S. Robb, H. Teeling, P. H. Seeberger, T. Schweder, C. J. Crawford, J.-H. Hehemann

**Affiliations:** 1 Faculty of Chemistry & Biology, BIOM, University of Bremen, Bremen, Germany; 2 MARUM, University of Bremen, Bremen, Germany; 3 Max Planck Institute for Marine Microbiology, Bremen, Germany; 4 Max Planck Institute of Colloids and Interfaces, Potsdam, Germany; 5 Institute of Pharmacy, University of Greifswald, Greifswald, Germany; 6 Institute of Marine Biotechnology, Greifswald, Germany; 7 Institute for Chemistry and Biology of the Marine Environment (ICBM), School of Mathematics and Science, Carl Von Ossietzky Universität Oldenburg, Oldenburg, Germany; 8 School of Chemistry, Trinity Biomedical Sciences Institute, Trinity College Dublin, Dublin, Ireland

## Abstract

**Background:**

Diatoms, a keystone phylum in Earth’s ecosystems, are responsible for substantial oxygen production and the fixation of carbon dioxide in the form of carbohydrates that fuel global food webs. They host diverse prokaryotes, yet how diatoms preferentially recruit those with complementary metabolic traits remains unknown.

**Results:**

We discovered that diatoms exude a C6-sulfated α-1,3-mannan that serves as a selective carbon source for adapted *Polaribacter*. Its structure was resolved using NMR spectroscopy, chromatography, chemical synthesis, and enzymatic dissection. Biochemical, physiological, and structural analyses demonstrated, that specialized Bacteroidota employ a four-enzyme pathway to metabolize this glycan. Metagenomic and transcriptomic data revealed that sulfated mannan utilization loci are globally abundant and actively expressed in surface ocean bacterioplankton. Because this mannan provides only carbon, oxygen, sulfur, and hydrogen, bacteria must obtain other essential elements elsewhere, reinforcing metabolic interdependence.

**Conclusions:**

Together, these results define a chemically specific interaction between diatoms and specialized bacteria that is mediated by a single sulfated polysaccharide and a dedicated four-enzyme degradation pathway. Presence of this pathway in marine metagenomes and transcriptomes indicates that a sulfated mannan from diatoms exerts selection pressure in the sunlit ocean microbiome.

Video Abstract

**Supplementary Information:**

The online version contains supplementary material available at 10.1186/s40168-026-02379-9.

## Main

Marine diatoms account for about 20% of global carbon dioxide fixation [1]. Up to half of their primary production is released into seawater [2, 3], and a major fraction of this material consists of diverse carbohydrates [4]. These include readily degradable glycans, such as laminarin, that support rapid bacterial growth, and chemically complex glycans, such as sulfated fucans, that resist degradation and contribute to the export of reduced carbon into the deep ocean [4, 5]. Given the scale of diatom primary production, their glycans may influence carbon cycling, carbon sequestration, and the organisation of bloom-associated microbiomes. However, the structures and quantities of most bloom-derived glycans remain unknown, which limits our ability to assess their ecological roles [6, 7].

Mannose-containing polysaccharides appear to be important in carbon storage and cycling [8–10]. Mannose contributes substantially to both particulate and dissolved organic matter, and several studies have reported mannose-rich glycans produced by diatoms, although their structures have not been resolved [3, 8, 11–13]. Metatranscriptomic and fluorescence in situ hybridisation studies have detected the upregulation of α-mannan-degrading enzymes in bacteria associated with diatom-dominated blooms, which suggests that mannose-based substrates may be actively metabolised in situ [14, 15], but the corresponding marine substrate has not been identified.

Here, we report the isolation of a structurally homogeneous, sulfated α-mannan exuded by the centric diatom *Conticribra weissflogii* and other diatoms. We determine its structure using nuclear magnetic resonance supported by synthetic standards, and we define the complete four-enzyme cascade that enables its degradation by bloom-associated Bacteroidota. Using these enzymes, we quantify mannan production and consumption. Finally, we use metagenomic datasets to assess the wider relevance of this pathway in surface ocean microbiomes [16].

## Results

### Diatoms may cultivate adapted bacterial taxa by exudation of sulfated mannan

**Fig. 1:**
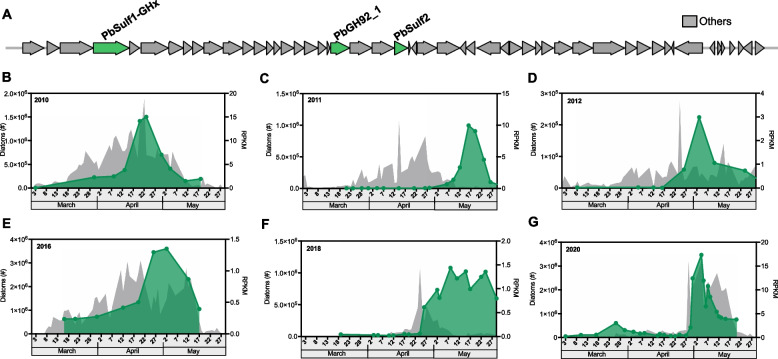
Specialized Bacteroidota with putative mannan utilization enzymes follow diatoms during algal blooms. **A** The organization of the studied mannan PUL (~ 87 kb) in *Polaribacter* sp. Hel1_33_78 is shown. The genes characterized in this study are highlighted in green, whereas other genes are shown in gray. The synteny of this PUL with other, closely related PULs is shown in the Supplementary information (Fig. S1B). **B**–**G** Shown are data from six years from the long-term ecological research station (LTER) on Helgoland. The diatom cells are followed by certain Bacteroidota taxa that have a polysaccharide utilization locus (PUL, Fig. S2) with putative genes encoding for enzymes that indicate utilization of a hypothetical, sulfated α-mannan secreted by diatoms. The DNA sequence of the mannan PUL of *Polaribacter* sp. Hel1_33_49 was mapped against metagenomes collected previously from the LTER Helgoland Roads. Gray shows diatom cell counts obtained by microscopy. Green shows the PUL abundance over years calculated in terms of Reads Per Kilobase per Million mapped reads (RPKM). The x-axis represents the date of each year. For more information, see “Materials and methods“ section

During the analysis of diatom bloom-associated metagenomes and bacterioplankton genomes, we noted the recurrence of bacterial taxa with a putative polysaccharide utilization locus (PUL) for mannan that consistently co-occurred with diatoms (Fig. 1). Here, we use the term ‘adapted’ to describe bacteria that encode dedicated PUL enabling the degradation of specific substrates. The PUL is found in certain marine *Bacteroidota* including *Formosa* spp., *Ochrovirga* spp., and *Polaribacter* spp., which are globally distributed and often highly abundant, like the diatoms they frequently associate with (Fig. S1). Some of these species can account for ~ 10% of the bacterioplankton cells surrounding diatoms [17–19]. We reasoned that, among many organic molecules, a mannan may contribute to a stable microbiome [15, 20–22]. To uncover this hypothetical mannan, we investigated the centric diatom *Conticribra weissflogii* (formerly *Thalassiosira weissflogii*), a diatom model species [23]. Notably, the diatom species composition varies annually, which may explain certain discrepancies between PUL abundance and diatom cell counts [17–19].

### A C6-sulfated α-1,3-mannan secreted by a diatom

We previously used anionic exchange chromatography (AEX) to show that several diatom species, including *C. weissflogii*, secrete a fucose-containing sulfated polysaccharide (FCSP) of unknown structure [24]. By refining AEX chromatography to achieve higher molecular resolution, we found that the material secreted by *C. weissflogii* comprises at least three anionic glycans: the FCSP, a glucuronomannan [25, 26] and a previously unknown homomannan. The FCSP and the glucuronomannan eluted at lower salt concentrations (0.5–1.2 M NaCl) and contained diverse monosaccharides, whereas, the homomannan eluted only at 2 M NaCl (Fig. S2A–C). Acid hydrolysis coupled with HPAEC-PAD (high-performance anion exchange chromatography with pulsed amperometric detection) revealed a composition of ~ 95 mol % mannose. Additional ion chromatography revealed that the mannan contains an almost equimolar ratio of sulfate to mannose (Fig. S2D).

During chromatography, concentration and dialysis, the mannan remained soluble in both seawater and deionized water. It reached solubility up to 20 mg mL^−1^, the highest tested, whereas other algal polysaccharides (agar, κ-carrageenan, alginate, and pectate) formed gels under seawater concentrations of calcium and magnesium ions [27]. These insoluble glycans may target, or be targeted by, distinct bacterial populations [21], in contrast to the soluble mannan. From 160 L of diatom culture, we obtained between 15 and 20 mg of the mannan (~ 100 µg L^−1^).

**Fig. 2:**
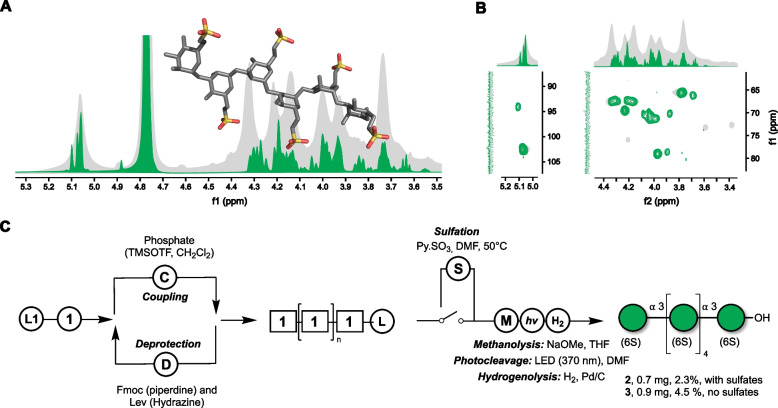
Diatoms synthesize a homomannan made of α−1,3-mannose-6-sulfate. In addition to chromatographic structure elucidation (Fig. S2B, D) we used automated glycan assembly (AGA) to synthesize a mannan oligosaccharide of the NMR-hypothesized structure of the diatom mannan and compared the two spectra. **A** ^1^H-NMR overlay of an automated glycan assembly derived synthetic α−1,3-mannose-6-sulfate hexaoligosaccharide (green) and the mannan purified from Conticribra weissflogii culture supernatant (gray). **B** Shown is an overlay of ^1^H-^13^C HSQC spectra from the synthetic mannan hexamer (green) and the purified diatom mannan (gray). NMR spectra at 700 MHz were collected at 20 °C. HSQC-TOCSY and ^1^H-^13^C HMBC can be found in supplementary data. **C** Scheme of chemical synthesis of mannan oligosaccharides. L1: Free reducing end solid-phase linker [28]. 1: Dibutyl 2-O-benzoyl-4-O-benzyl-6-O-(9-fluorenylmethoxycarbonyl)−3-O-levulinoyl-1- phosphate-α-D-mannopyranoside

For configuration and connectivity, we used one- and two-dimensional NMR (Fig. 2A, B). Details are provided in the supplementary information. Briefly, the major component was α-1,3-D-mannopyranose with the C6-carbon de-shielded at 67.5 ppm, consistent with 6-*O*-sulfation and confirming the equimolar mannose-to-sulfate ratio (Fig. 2B, Supplementary text). For further verification, we synthesized a mannan oligosaccharide and used it as a reference for comparative NMR.

We used automated glycan assembly to synthesize a reference mannan oligosaccharide [29, 30]. As a building block, we prepared ethyl-2-*O*-benzoyl-4-*O*-benzyl-6-*O*-(9-fluorenylmethoxycarbonyl)-3-*O*-levulinoyl-1-thio-α-D-mannopyranoside, which was converted into a dibutyl phosphate donor [1] (Fig. 2C, Supplementary text) [31]. This enabled the automated assembly of a hexa-α-1,3-mannose bearing one sulfate on each C6. Comparison of NMR spectra from natural and synthetic mannan showed deviations of < 0.03 ppm in the proton and < 0.1 ppm in the carbon dimension (Fig. 2A, B, Table S1), confirming the structure as a C6-sulfated α-1,3-mannan (Fig. S14–19).

### *Polaribacter* spp. can metabolize the mannan

We tested with *Polaribacter* strains—Hel1_33_49, _78 and _96 whether the PUL [32] enables mannan metabolism. These strains have 100% identical 16S rRNA genes and 98.5% genome-wide average nucleotide identity (ANI) [33], providing three biological replicates. They have the PUL, indicating a similar ability to metabolize the mannan. Accordingly, when the sulfated mannan was provided as a limited carbon source, the three strains grew better (Fig. S3–S6, Table S2). As a negative control, we used *Polaribacter* sp. Hel1_88 [19, 32], which lacks the mannan PUL [20]. The Hel1_88 strain grew less (OD_600_ 0.212) on mannan in comparison to Hel1_33_49 (OD_600_0.410), _78 (OD_600_0.411), and _96 (OD_600_0.383). The mannan remained unconsumed in the culture of *Polaribacter* sp. Hel1_88 (Fig. S4).

In 2020, we showed *Polaribacter* spp. transcribe this PUL in situ [15] in the North Sea. To confirm transcription and translation of enzymes capable of depolymerizing the mannan, we used cell lysates of *Polaribacter* sp. Hel1_33_78 for activity tests. Enzymes were extracted from bacterial cultures grown with or without the mannan (Fig. S5, Fig. S6). The enzymes in the lysates from cells grown with the mannan digested the mannan. These expression and activity assays suggest these enzymes of adapted *Polaribacter* spp. catalyze the metabolism of the mannan. The in situ enzyme expression indicates bacteria respond to and interact with the mannan released by diatoms into their environment**.**

### The bacterial enzymes can hydrolyze the mannan

**Fig. 3:**
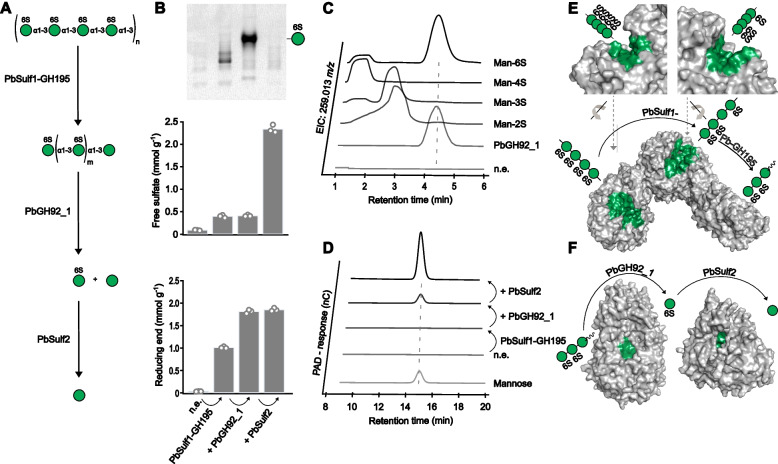
Four bacterial enzymes in three genes hydrolyze the sulfated α-mannan from diatoms. **A** Suggested degradative pathway for sulfated α-mannan. **B** Enzymatic digestions of the mannan via sequentially added PbSulf1-GH195, PbGH92_1, and PbSulf2. Top: Product separation by fluorophore-assisted carbohydrate electrophoresis using 2-aminoacridone as fluorophore. Middle: Free sulfate quantification. Bottom: Reducing end-assay. Bars represent mean of *n* = 3, and circles display individual data points. n.e. = no enzyme. **C** Digestion of oligosaccharides produced by PbSulf1-GH195 through PbGH92_1 was analyzed using LC–MS. Extracted ion chromatograms at 259.013 m/z of the digestion compared with synthesized or commercially purchased mannose-sulfate standards. Man-2S: Mannose-2-Sulfate; Man-3S: Mannose-3-Sulfate; Man-4S: Mannose-4-Sulfate; Man-6S: Mannose-6-Sulfate. **D** Mannose release after a sequential digestion of the mannan by PbSulf1-GH195, PbGH92_1 and PbSulf2, detected using HPAEC-PAD. **E**–**F** Structural models of enzymes involved in the degradative cascade were generated using AlphaFold2. Active site regions (green color) were identified using structural superimpositions with previously characterized enzymes. A simplified version of the degradative cascade is depicted according to the symbol nomenclature for glycans [35]. **E** The PbSulf1-GH195 tandem enzyme, with the rotated active site region of the Sulfatase- and GH195-domain shown in top left and right, respectively. Gray arrows indicate the rotation axis of each domain towards the full-length protein (below). **F** Exo-acting PbGH92_1 and PbSulf2 have a pocket-shaped active site

To verify enzyme activity and specificity, we used recombinant gene expression, enzymology and structural biology (Fig. 3). The PUL comprises 23 genes (Fig. 1), 21 of which encode putative enzymes. We cloned these genes from *Polaribacter* sp. Hel1_33_78 into vectors, transformed and expressed them in *Escherichia coli*, and conducted activity tests using purified mannan, synthesized mannan oligosaccharides, and para-nitrophenyl-labeled substrates (PNP-substrates). The products of these substrates digested with the enzymes were analyzed by fluorophore-assisted carbohydrate gel electrophoresis (FACE) and reducing sugar assays (Table S3). To further substantiate the findings, we obtained homologous enzymes from another marine *Bacteroidota*, *Ochrovirga pacifica* S85, which was isolated from the Pacific Ocean [34], with 69.8% ANI with the *Polaribacter* sp. Hel1_33_78 genome.

*O. pacifica* has a distantly related version of the PUL [36], with 68.8% ANI to the mannan PUL of *Polaribacter* sp. Hel1_33_78. We found that the four enzymes are present in both PULs (*Polaribacter*: PbSulf1-GH195, PbGH92_1, PbSulf2; *O. pacifica*: OpSulf1, OpGH195, OpGH92, OpSulf2). These enzymes, comprising one unknown family GH195, one GH92 and two sulfatases, hydrolyzed the mannan into mannose and sulfate (Fig. 3A). The remaining 18 putative enzymes were cloned and tested but appeared inactive on the mannan (Table S3). For one sulfatase (OpSulf1), the validity of biochemical experiments could be supported by X-ray crystallography analysis. For the other enzymes, which did not crystallize, we used in silico generated AlphaFold2 [37] structure models to falsify the biochemical data.

Mannan degradation is accomplished in four enzymatic steps. The first two steps are completed by the multimodular enzyme PbSulf1-GH195. The enzyme contains a T9SS domain for extracellular secretion. The sulfatase and GH195 domains in PbSulf1-GH195 work sequentially to catalyze the first and second steps of the cascade. The sulfatase domain belongs to the S1 family, subfamily 51 [38]. It is the first described activity of this subfamily and the first known sulfatase with this specificity. The GH195 domain is distantly related to endo α-1,2 mannanases of the GH99 family and may therefore classify as a new family [39]. Separating and visualizing fluorophore-labeled reaction products on FACE gels showed hydrolysis of the mannan by PbSulf1-GH195, producing a ladder-like pattern of oligosaccharides typical for endo-acting enzymes [40] (Fig. 3B). The tandem enzyme released sulfate and oligosaccharides as shown by ion chromatography and reducing sugar assays (Fig. 3B). Liquid chromatography mass spectrometry (LC–MS) showed that the released oligosaccharides constituted sulfated hexose oligomers with degrees of polymerization between three and seven (Fig. S7). The oligosaccharides were consistently missing one sulfate group, e.g., a mannose tetrasaccharide had only three sulfate groups, further indicating that both catalytic domains were active on the mannan.

We reproduced the results with enzymes from *O.* *pacifica,* which encodes the two catalytic domains as two separate genes. The enzymes OpSulf1 and OpG195x exhibit 53% and 36% amino acid identity to the respective PbSulf1-GH195 domains. The separated enzymes showed that prior sulfatase activity is indeed necessary for GH195 activity, suggesting that this enzyme requires a desulfated mannose for productive binding (Fig. S8). The AlphaFold2 model of PbSulf1-GH195 showed two appended catalytic domains with open, extended clefts (Fig. 3E), typical for endo-acting glycoside hydrolases [40] and sulfatases [41], corroborating the biochemical experiments. We obtained diffraction data from a crystal structure for the OpSulf1 at 1.49 Ȧ resolution (Fig. S9, Table S4). The crystal structure confirmed an extended substrate binding cleft, compatible with the elongated mannan chain. The conserved catalytic residues present in the center may remove one sulfate from the center of the bound mannan chain, which then becomes accessible to the GH195 (Fig. S9B, C). In conclusion, we identified and characterized two new enzyme activities, one endo C6-sulfate α-1,3-mannanase forming a new CAZy family, and an endo C6-sulfate α-1,3-mannan sulfatase. These predicted outer membrane enzymes may produce oligosaccharides of suitable size and shape for import into the periplasm.

The third step in the mannan degradation cascade was catalyzed by the periplasmic PbGH92_1, a mannosidase that removed C6-sulfated mannose from the oligosaccharides. Periplasmic localization of PbGH92_1, which belongs to the GH92 family [42], was predicted by the Sec/SPI signal peptide [43]. The GH92 family includes Ca^2+^-dependent exo-acting α-1,2/3/4/6 mannosidases, but an enzyme, specific for sulfated mannan remains unknown. Hydrolysis of oligosaccharides into mannose-6-sulfate and a minor amount of mannose was shown by FACE, reducing sugar assays and HPAEC-PAD (Fig. 3B, D). The mannose-6-sulfate product was verified using LC–MS (Fig. 3C). For this analysis, we synthesized mannose-2/3/4-sulfate isomers (Supplementary text, Data S1, Fig. S10). The retention times revealed that only the mannose-6-sulfate aligned with the enzyme product peak and therefore confirmed this isomer as the product of the PbGH92_1 (Fig. S11). The AlphaFold2 model showed a pocket topology of size and shape typical for an exo-acting GH (Fig. 3F), binding one monomer in the − 1 subsite [40]. Conclusively, PbGH92_1 is a 6-sulfate α-1,3-mannosidase, representing a new specificity within the GH92 family.

The fourth step was catalyzed by PbSulf2 that desulfates mannose-6-sulfate to yield mannose. A periplasmatic location was predicted by a Sec/SPII signal peptide. PbSulf2 is classified into sulfatase family S1, subfamily 11. Enzymes in this subfamily have been described to act on glucosamine-6-sulfate [44] and 6-sulfated porphyran oligosaccharides [45], but not on mannose-6-sulfate. PbSulf2 removes a sulfate group from mannose-6-sulfate, as shown by appearance of the final product mannose in HPAEC-PAD measurements (Fig. 3D) and the disappearance of sulfated mannose in a complementary FACE experiment (Fig. 3B). The AlphaFold2 model aligned with the two previously described subfamily members (PDB ID Codes: 5G2V/7LJ2, rmsd: 0.613 Ȧ/0.845 Ȧ) and showed a pocket topology typical for exo-acting sulfatases [41]. PbSulf2 is a mannose-6-sulfate sulfatase, representing a new specificity within this enzyme family.

In summary, we elucidated a minimal enzymatic cascade consisting of four enzymes that achieve complete hydrolysis of the mannan into mannose and sulfate. The oligo- and monosaccharide structures produced by the enzymes confirmed the mannan structure solved with NMR.

### Biocatalytic quantification of mannan synthesis and degradation

**Fig. 4:**
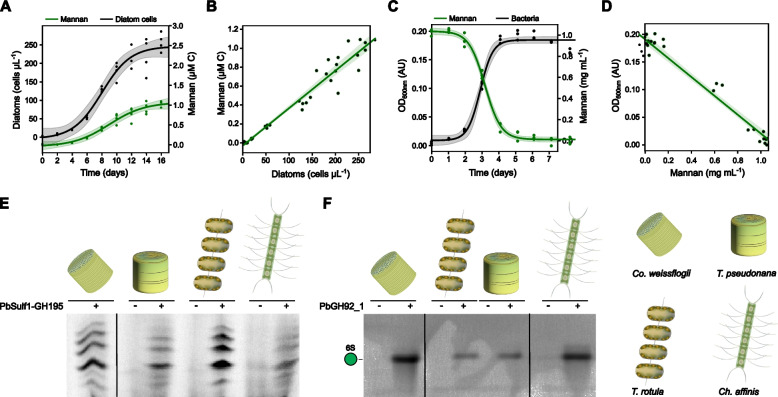
Synthesis of mannan by diatoms and consumption by bacteria, quantified with the mannan-degrading enzymes. **A** Growth analysis of *Conticribra weissflogii* cultures using cell counts (black) and enzymatic quantification of sulfated α-mannan (green) in the anionic fraction of the culture supernatant. Data points were modeled using a logistic growth function employing the non-linear least squares method for fitting. *n* = 4. **B** Linear regression analysis of diatom cell counts vs. mannan C production. The net production of mannan per cell was 3.9 fmol C cell-1 with an *R*^2^ = 0.95. **C** *Polaribacter* sp. Hel1_33_49 grown with purified mannan for one week at 18 °C. The black line represents optical density measured at 600 nm. The green line shows the mannan consumption as detected via enzymatic quantification. Data points were modeled using a logistic growth function employing the non-linear least squares method for fitting. *n* = 3. **D** Linear regression analysis of mannan concentration vs. optical density of the growth curve. **E**–**F** Mannan identification in anionic exudates purified from 0.5 to 5 M NaCl from AEX from *Conticribra weissflogii*, *Thalassiosira pseudonana*, *Thalassiosira rotula* and *Chaetoceros affinis*. Products were digested with PbSulf1-GH195 (**E**) and PbGH92_1 (**F**) and analyzed via fluorophore-assisted carbohydrate electrophoresis using 8-aminonaphthalene-1,3,6-trisulfonic acid (**E**) or 2-aminoacridone (**F**) as fluorophore. Negative controls (−) contained heat inactivated enzyme

To quantify mannan production by diatom cells and its consumption by adapted *Polaribacter* species, we developed a quantitative assay using the characterized enzymes. The assay involved a combination of chromatography and enzyme digest steps for structure-specific quantification. We measured the enzyme product, mannose, that provides a much stronger signal than the undigested polysaccharide, with different quantitative approaches, e.g., reducing sugar assay or HPAEC-PAD (Fig. S12A). Chromatographic separation and enrichment of the mannan were crucial for enzyme digestion. Without this purification step, we found the enzymes remained inactive, potentially due to sulfate groups from FCSP or other sulfated glycans binding to and interfering with the enzymes in the mixture [46]. After chromatographic isolation of the mannan, the biocatalytic assay detected up to 842 ± 62 µg L^−1^ in the diatom culture supernatant (Fig. 4A). The concentration was positively correlated with the number of diatom cells (*y* = 3.97 × 10^–3^ *x *2.94 × 10^–2^, *P* = 1 × 10^–23^, *R*^*2*^ = 0.95). Each individual *C. weissflogii* cell produced, on average, a net amount of 3.9 fmol carbon in form of mannan (Fig. 4B). Substantially less mannan was present in diatom cells or particles (Fig. S12B, C). Notably, this new biocatalytic assay detects almost ten-fold higher concentrations (842 ± 62 µg L^−1^) compared to our initial assessment (~ 100 µg L^−1^), based on the preparative mannan purification yield, highlighting the analytic value of enzyme-based assays.

Next, we conducted growth experiments with *Polaribacter* sp. Hel1_33_49 and applied the biocatalytic assay to quantify mannan consumption (Fig. 4C). The bacteria grew to an OD_600_ of 0.2 when providing 0.1% (w/v) of the mannan as the limiting carbon source. Mannan concentration and bacterial abundance were negatively correlated (*y* =− 5.62 *x* + 1.09, *P* = 1 × 10^–18^, *R*^2^ = 0.96) (Fig. 4D). Conclusively, under nutrient-replete conditions, the bacteria can remove the mannan within a few days.

To test whether other diatoms also release this type of mannan, we adapted the biocatalytic assay into a qualitative version that rapidly identifies the structure in different species. We tested *Thalassiosira pseudonana*, *Thalassiosira rotula* and *Chaetoceros affinis,* genera of global ecological and biogeochemical relevance [17]. We used AEX to enrich the mannan from each culture supernatant, applied the enzymes and analyzed the products using FACE (Fig. 4E, F). For each diatom, the first two enzymes (PbSulf1-GH195) produced the same set of oligosaccharides that we identified above as oligomers of C6-sulfated α-1,3-mannose missing one sulfate group. PbGH92_1 released mannose-6-sulfate, showing that the same mannan structure is produced by different diatoms common around the globe.

### Mannan draws adapted bacteria towards diatoms around the globe

To test the hypothesis that the mannan selects for bacteria with these enzymes we quantified the mannan PUL within the *Tara* Oceans metaG dataset [47]. As negative and positive controls for glycan structure-based selection of adapted microbiome members, we used bacterial PULs specific for known glycans from red, green and brown algal phyla. Preconditions for the PULs used as controls were that their target glycans are soluble and therefore accessible to planktonic bacteria, that the glycan structure has been verified by NMR and/or other methods, and that the structure and function of the PUL or of a closely related variant has been empirically verified with biochemical experiments. As negative control we used porphyran, a mixed linked α-, β-galactan that is also sulfated on C6, and the corresponding PUL from the flavobacterium *Zobellia galactanivorans* Dsij^T^ [48, 49]. Compared to diatoms, the porphyran-producing red macroalgae *Porphyra* spp., are sessile, require rocks or other hard substrates and grow along coasts. This range restriction makes the corresponding porphyran-PUL a suitable negative control. This type of analysis has been carried out previously [50–52].

As a positive control, we used laminarin (chrysolaminarin), a β-glucan that is also produced by diatoms, and the corresponding PUL from a metagenome-assembled genome (MAG C_MB344, accession: GCA_943788335) of a flavobacterial *Aurantivirga* species [15, 53, 54]. Finally, we used ulvan, a sulfated rhamnogalacturonan produced by green macroalgal species, including *Ulva* spp. *Ulva* spp. grows attached and planktonic, moves via currents between coastal and offshore regions [55]. Hence, whether the corresponding PUL can serve as negative or positive control was unclear. The corresponding PUL [56] is from the flavobacterium *Formosa agariphila* KMM 3901^T^ [57, 58].

Mannan PUL abundances ranged from 0.0025 to 0.41 RPKM in the 45 sampled sites including open and coastal ocean areas of the Atlantic, Pacific and Indian Oceans (Fig. S13A). Higher mannan PUL abundances were detected at upwelling sites (Benguela 0.41 RPKM, Chile 0.06 RPKM, Peru 0.03 RPKM, California 0.04 RPKM), where higher nutrient concentrations enhance diatom-dominated algal blooms [59, 60]. The positive control, laminarin PUL, showed comparable abundances between 0.006 and 2.27 RPKM (Fig. S13C). The porphyran PUL showed the lowest RPKM values of the tested glycans and was absent in the open ocean (Fig. S13E), as expected for this negative control. The ulvan PUL showed abundances between 0.002 and 0.08 RPKM, but at different locations compared to the mannan and laminarin PULs (Fig. S13D). The observed differences between the compared groups were significant across the dataset (Friedmann-Test: *P* = 6 × 10^–21^).

**Fig. 5:**
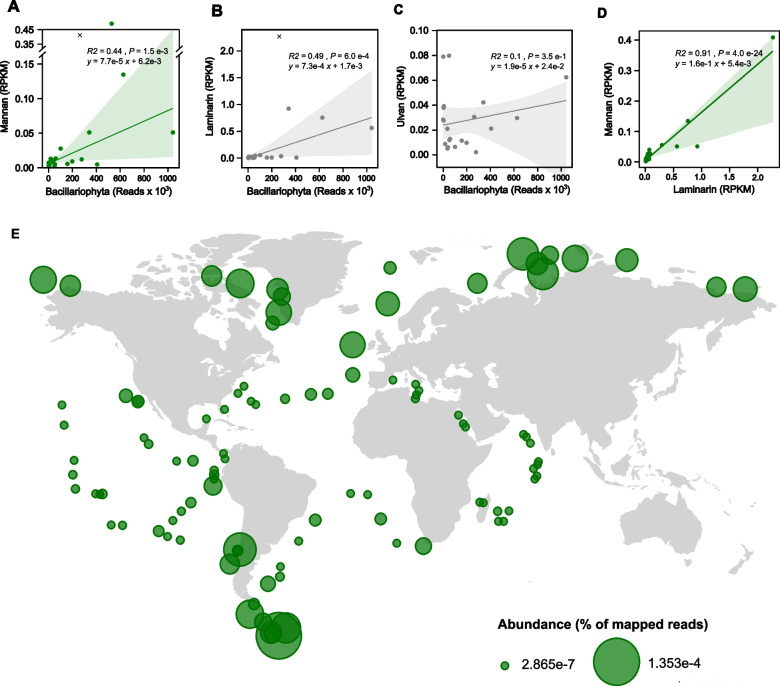
Bacteria with mannan-degrading enzymes follow diatoms around the globe. Global abundance of the mannan utilization locus from *Polaribacter* sp. Hel1_33_49 from *Tara* Oceans database. The DNA sequence of the mannan PUL was mapped against the raw metagenome reads of *Tara* Oceans surface water samples, filtered at sizes ranging 0.2–3 µm, across 45 different stations [47]. Read abundances were calculated in terms of RPKM and normalized by length of the PUL. Corresponding analyses for the laminarin PUL from *Aurantivirga* MAG C_MB344 [15], the ulvan PUL from *Formosa agariphila* KMM 3901^T^ [56] and the porphyran PUL from *Zobellia galactanivorans* Dsij^T^ [48] were carried out. For more information, see Fig. S13A, C-E. **A**–**C** Linear regression analyses of raw read abundance of Bacillariophyta in V9 rDNA metabarcoding datasets from *Tara* Oceans surface water samples filtered at sizes ranging 5–20 µm across 35 stations versus RPKM of different PULs across the same stations. Please note: no diatom data were available for the remaining stations. Outliers were identified individually for each plot using the studentized residuals method with a threshold of three and excluded from the linear regression. Plots display the correlation analysis of Bacillariophyta vs. **A** mannan PUL, **B** laminarin PUL, and **C** ulvan PUL. **D** Linear regression analyses of mannan PUL and Laminarin PUL RPKM within the *Tara* Oceans metagenomes. **E** For expression analysis of the PUL, the GH92 sequence from the *Polaribacter* cascade was searched against the *Tara* Oceans OM-RGCv2 database, which includes metatranscriptomic (metaT) data. The sequence was queried using BLASTp, with an E-value cutoff of ≤ 1 × 10^⁻20^, via the Ocean Gene Atlas v2 web platform (https://tara-oceans.mio.osupytheas.fr/ocean-gene-atlas/). The resulting hits were used to calculate relative abundance, expressed as the percentage of mapped reads, and were visualized on a global map. The detection of transcripts indicates the global distribution and expression of this specific mannan-degrading GH92 enzyme, suggesting that the associated bacteria contribute to the degradation of diatom-derived mannan at a global scale

Metagenomics of bacterioplankton and eukaryotic 18S rRNA-based taxonomy indicated an association of the mannan PUL with marine diatoms. To verify that diatoms were the main phytoplankton associated with the large RPKM values of PULs at the time of sampling for the *Tara* Oceans metaG dataset, we used previously reported total V9 read numbers as a proxy for diatoms and correlated those with PUL reads [60]. This approach was previously verified by showing that V9 reads correlate with diatom algal cell counts [61]. At the sites where microalgae were measured, diatoms were a major phytoplankton group [60]. Linear regression analyses showed that the abundance of the mannan PUL correlated with diatom read numbers (*y* = 7.7 × 10^–5^ *x* + 6.2 × 10^–3^, *P* = 1.5 × 10^–3^, *R*^*2*^ = 0.44) (Fig. 5A). The laminarin PUL also significantly correlated with diatom abundances (*y* = 7.3 × 10^–4^ *x* + 1.7 × 10^–3^, *P* = 6.0 × 10^–4^, *R*^*2*^ = 0.49) (Fig. 5B). In contrast, we did not observe correlation between the ulvan PUL and diatom cell abundances (*P* = 0.35; *R*^2^ = 0.05), which is consistent with the synthesis of this glycan by green algae instead of diatoms (Fig. 5C).

Diatoms are the only known microalgae that synthesize both, the mannan and the laminarin. Hence, we asked whether the PULs for those two glycans co-occurred, which would further support that diatoms are the source of these carbon-rich structures. We found a strong linear relationship between mannan and laminarin PULs (*y* = 0.158 *x* + 5.35 × 10^–3^, *P* = 4 × 10^–24^, *R*^*2*^ = 0.91, Fig. 5D). In contrast, the PULs for mannan and ulvan did not correlate (*P* = 0.414; *R*^2^ = 0.02). Finally, we queried for gene expression of the *Polaribacter* GH92 and found transcripts in the *Tara* Oceans metaT dataset [47]. The presence of transcripts suggests associated bacteria degrade the mannan released by diatoms globally (Fig. 5E). Our bioinformatic analyses indicate that spatially, temporally and phylogenetically separated micro- and macroalgae produce distinguished glycans. In conjunction, the diatom glycans seem to select for specifically adapted bacteria. Their exudation may contribute to the formation of functional and predictable, host-specific microbiomes in the sunlit ocean.

## Discussion

We show that diatoms secrete a sulfated mannan and that this glycan forms a selective interaction with specialized Bacteroidota. The mannan can be isolated directly from dissolved organic matter and its structure was resolved by NMR and validated with chemically synthesised standards. The structural homogeneity is notable because algal sulfated glycans are often too heterogeneous for direct NMR assignment [5, 62]. *Polaribacter* spp. contain a compact but complete set of enzymes that initiate extracellular hydrolysis, remove sulfate groups and deconstruct the α-1,3 backbone. This contrasts with the large cascades that can be required to degrade algal fucoidan [5, 63]. We disclose a new glycoside hydrolase family within a bimodular, endo-acting enzyme and determine the first crystal structure of an S1_51 sulfatase.

Using these enzymes, we developed an assay to quantify mannan production and degradation. The glycan occurs predominantly in the dissolved fraction and is consumed efficiently by *Polaribacter* spp., linking diatom production directly to heterotrophic utilization. This assay enables the contribution of the mannan to carbon flow to be quantified during diatom blooms. Ecological data indicate that this pathway is widespread [15]. Mannan PULs recur seasonally during spring diatom blooms at Helgoland, and their abundance co-varies with *Polaribacter* spp. In situ visualization studies have shown that *Polaribacter* spp. expressing GH92 enzymes are associated with algal-derived material during North Sea spring blooms, supporting the ecological relevance of particle-associated niches for mannan-specialist taxa [14]. In global ocean metagenomes, mannan PULs are broadly distributed, and multiple diatom genera, including *Thalassiosira* and *Chaetoceros*, produce sulfated α-mannans [17, 64]. This supports the ecological relevance of mannan turnover across marine environments.

The biochemical properties of the mannan may provide a mechanistic basis for its effects. Sulfated polysaccharides can act as polyelectrolytes with high surface activity and can interact with positively charged amino acid residues on proteins [46]. Sulfation at C6 extends the sulfate group away from the sugar backbone, increasing access to protein surfaces [48]. Such interactions may alter bacterial attachment to diatoms and interfere with bacterial surface proteins. In this context, bacteria adapt either by degrading the mannan or by minimising interactions with it. *Polaribacter* spp. invest in the mannan PUL, gaining carbon and removing a surface-active polymer. In contrast, groups such as SAR11 rely on low binding cell surfaces and focus on smaller metabolites [65, 66]. These strategies resemble known competition–dispersal trade-offs observed in other marine bacteria [65, 67, 68].

Together, these findings define a specific interaction between diatoms and specialized Bacteroidota. The diatom mannan and the dedicated bacterial four-enzyme cascade suggest a recurring partnership. At the scale of blooms, such partnerships could suggest principles of domestication [69–72], whereby hosts favour particular microbial associates. Therefore, secreted diatom sulfated glycans can both nourish and constrain bacteria and mediate stable associations. These interactions influence how carbon is either remineralized or sequestered during phytoplankton blooms.

## Materials and methods

### Mannan PUL abundance in *Tara* Oceans dataset

Metagenome data of bacterioplankton during spring phytoplankton blooms was generated in previous studies [15, 21, 22, 73, 74]. For the global analysis, we used metagenome data from the *Tara* Oceans Expedition [47]. The surface water metagenomes (0.2 to 3 µm) from 45 different stations were included in the analysis. Mapping of PULs was done with Bowtie2 [75] in the SqueezeMeta v1.6.2 pipeline. PUL abundances as reads per kilobase per million mapped reads (RPKM) were calculated using the formula: (metagenome reads mapped to the PUL*10^6^)/(length of PUL in kbp × total reads in a metagenome). For correlation of PULs to diatom abundance, we extracted the read numbers from 25 surface water samples (5–20 µm) from the eukaryotic V9 metabarcoding data [60].

### Mannan purification

*C. weissflogii* was cultured non-axenic in NEPCC medium [76]. Diatom growth was monitored by cell counting using a Neubauer counting chamber (Marienfeld, Königshofen, Germany). Cells were separated from culture the supernatant using a continuous flow centrifuge (Thermo Fisher Scientific, Walthan, MA, USA) set at 150 mL min^−1^ and 6800 × *g*. The culture supernatant was filtered using a peristaltic pump through Whatman® glass microfiber filters (Grade GF/F, 0.70 μm). 160 L of *C. weissflogii*, cell and debris free, culture supernatant were loaded onto a preparative scale, self-assembled AEX column. Elution occurred with appropriate NaCl concentrations. Samples were dialyzed against MilliQ water (Merck) and freeze-dried prior to monosaccharide composition and sulfate analysis.

### Monosaccharide composition and sulfate content analyses

Purified mannan was dissolved in MilliQ water at 1 mg mL^−1^ and hydrolyzed by 1 M HCl (100 °C, 24 h) in in pre-combusted glass vials. HCl was removed by vacuum or diluted 1:1000 in MilliQ water (Merck) prior to further analyses. Monosaccharide measurements occurred as described previously using a Dionex ICS-5000 + system (Thermo Fisher Scientific) [77] and for sulfate measurements we used a Metrohm 761 compact ion chromatograph (Metrohm, Herisau, Switzerland). All samples for monosaccharide and sulfate composition were prepared in triplicates.

### Cloning of enzyme genes into plasmids, recombinant expression and purification

Genes were cloned using the USER cloning or Gibson assembly methodologies (New England Biolabs, Ipswich, NE, USA) with genomic DNA from *Polaribacter* sp. Hel1_33_78. Primer pairs are provided in supplementary table S2. USER cloning constructs were cloned into the pet28A vector [78] without signal peptides [79]. Clones were validated by DNA sequencing. Plasmids transformed into *E. coli* BL21 (DE3) were induced in 1 L lysogeny broth (LB) medium containing kanamycin and isopropyl-β-D-1-thiogalactopyranoside (IPTG). For sulfatase activation, the formylglycine generating enzyme plasmid (Addgene plasmid repository, Watertown, MA, USA) [80], which was co-transformed, was induced with L-arabinose and ampicillin added right before cooling to 12 °C. Protein purification was conducted using standard procedures [81].

### Enzyme activity and specificity assessment

Enzyme reactions were carried out in 10 mM MOPS pH 7.5 and 500 mM NaCl with 0.1% (w/v) of mannan and enzymes were added in excess (0.01–0.1 mg mL^−1^). 3.5% Sea Salts (Sigma Aldrich, Burlington, MA, USA) were also added. Enzyme reactions were monitored using fluorophore-assisted carbohydrate electrophoresis (FACE) with carbohydrates labeled with 2-aminoacridone as described in [82]. Reducing-end measurements were carried out using PAHBAH as detection reagent as described previously [83]. The release of sulfate and mannose from enzymatic reactions were measured using ion chromatography and HPAEC-PAD. Enzymatic assays were carried out as technical replicates in triplicates unless otherwise stated.

### Nuclear magnetic resonance and chemical synthesis of oligosaccharides

All chemicals used were reagent grade and used as supplied unless otherwise noted. A detailed description of the structural elucidation and glycan synthesis processes is provided in the supplementary information. The automated syntheses were performed on a custom-built synthesizer (Potsdam-Golm, Germany) [30, 31, 84, 85]. More synthesis and analysis details are provided in the Supplementary information.

### Crystallization, data collection, structure solution, refinement and AlphaFold2

Crystallization was performed in two drop 96-well crystallization plates in sitting drop format using commercial crystallization screens. Diffraction data was collected on DESY beamline P11. Measured reflections intensities were indexed, integrated and scaled using the autoproc pipeline available at the beamline facility. OpSulf1 structure was solved using molecular replacement with the AlphaFold2 predicted model as reference in PHASER [86]. For automatic model building we used BUCCANEER [87]. Refinement was done in REFMAC5 [88] and COOT [89]. Model and structure factors for OpSulf_1 were deposited in the Protein Data Bank (PDB) with accession 9FVT. Corresponding data-processing and refinement statistics are summarized in Table S4. The structural comparison of OpSulf with homologs were performed using the PyMOL v.2.3.2 (Schrödinger, New York, NY, USA). For enzymes that did not crystallize we used models generated with AlphaFold2 for structural analyzes [37].

### Liquid chromatography-mass spectrometry of mannose-sulfate and mannan digests

Standards and digests were diluted in 90% UHPLC-grade and 10% (v/v) MilliQ water (Merck) for LC–MS measurements. 5 µL were injected to an Accucore-150-amide HILIC column (Thermo Fisher Scientific) held at 60 °C on a Thermo Fisher Vanquish Horizon UHPLC coupled to a Thermo Fisher Q-Exactive Plus MS. Mobile phases A and B consisted of 10 mM ammonium formate at pH 5 and UHPLC-grade acetonitrile, respectively. A gradient started after 1 min at 90% B and decreased from 90 to 40% B over 40 min at a flow rate of 0.4 mL min^−1^. The column was equilibrated at 90% for 16 min at the end of each run. Mono- and oligosaccharides was detected in negative mode. The detailed MS conditions are provided in the Supplementary information.

### Bacteria cultivation

Growth experiments with *Polaribacter* spp. Hel1_33_49/78/96 and Hel1_88 was carried out in HaHa-medium supplemented with 0.1 mg mL^−1^ yeast extract and 2 mg mL^−1^ mannan from either *C. weissflogii* or *C.* *affinis* [90]. We used *Polaribacter* sp. Hel1_33_78 for in depth physiological experiments. Cells were harvested by centrifugation, the pellets chemically lysed. Lysed cells, enzymes were activity tested on *C*. *weissflogii* and *C.* *affinis* mannan. Digests occurred overnight using 3.5% Sea Salts (Sigma), 10 mM MOPS pH 7.5 and 0.5 mg mL^−1^ mannan and were analyzed using carbohydrate-polyacrylamide gel electrophoresis (C-PAGE). C-PAGE analysis was carried out as described in [91] with modifications described in the SI. *Polaribacter* sp. Hel1_33_49 was grown in 3.5% Sea Salts (Sigma) with supplements [90]. The mannan was used as limiting carbon source at a concentration of 1 mg mL^−1^. Growth curves (absorbance 600 nm) were plotted with a logistic growth function using the non-linear least squares method. Substrate consumption was verified qualitatively using C-PAGE.

### Enzyme assisted quantification of the mannan

Cultures of *Conticribra weissflogii* were grown in NEPCC medium as described above. Subsamples of 200 mL of the culture were filtered to remove cells and debris. The mannan in the filtrate was purified using AEX. After desalting the mannan was digested PbSulf1-GH195 and PbGH92_1. Undigested sample served as negative control. Detection occurred with the PAHBAH assay. Results were compared with a standard curve containing defined amounts of mannan. The recovery was determined 80 ± 5% (*n* = 3) by comparison to untreated mannan (Fig. S7C).

### Extraction of mannan from cellular, diatom biomass

After diatom cultivation the cell pellet was freeze-dried and sequentially extracted using MilliQ water (Merck), 0.4 M EDTA (pH 8) and 4 M NaOH with 0.1% NaBH4 [24]. For 1 g of biomass 20 ml of sample were used. In each extraction step the sample was sonicated and centrifuged at 14.000 × g for 10 min. NaOH extracts were neutralized to pH 7.5 using HCl and diluted in MilliQ to < 500 mM NaCl. The mannan content in all extracts was determined as described above.

### Biocatalytic detection of mannan in multiple diatom species

The diatom strains *Thalassiosira pseudonana*, *Chaetoceros affinis* and *Thalassiosira rotula* were cultivated as described above. 20 L of culture supernatant were concentrated after filtration using GF/F. The filtrate was concentrated by ultrafiltration and then dialyzed to remove salts. The dialyzed concentrate was purified by anionic exchange chromatography. The purified mannan was again dialyzed and then lyophilized. The dialyzed and freeze-dried samples were used as substrates for enzymatic assays using PbSulf1-GH195 and PbGH92_1. Digested samples were analyzed with FACE [77].

## Supplementary Information

Supplementary Material 1: Detailed materials and methods. Supplementary text. Figure S1. (A) PUL structures in *Polaribacter* sp. Hel1_33_49/78/96 and *Ochrovirga pacifica* S85. The genes displayed in green are subject of this study. (B) Synteny plot: The Helgoland Roads dataset, comprising approximately 90 metagenomes, was manually searched for mannan PULs. For the years 2010–2018, only Illumina-based metagenomes were available; due to the short-read sequencing technology, no complete PULs could be recovered, and only truncated subsets of the mannan PUL were detected. In contrast, PacBio metagenomes sequenced in 2020 enabled the identification of a complete homolog of the mannan PUL in a *Polaribacter* MAG (X_MB188), corresponding to the studied *Polaribacter* strain (Hel1_33_49, as shown in the figure). Both PULs displayed a high level of synteny (~ 100% identity), particularly across the three previously studied and characterized enzymes (highlighted in green in the figure). The corresponding genes also exhibited a high degree of sequence conservation across all other detected PULs. Figure S2. Purification of a sulfated mannan exuded by *Conticribra weissflogii*. (A,C) Anion exchange chromatography of culture supernatant of *C. weissflogii* with stepwise increase of NaCl concentration. Total carbohydrate signals of each fraction are displayed as black dots, and stepwise NaCl elution profiles as dashed lines. (A) Step size of 0.5 M NaCl (B) Relative monosaccharide composition after acid hydrolysis of eluted fractions from analytical replicates (n = 3) (C) Optimization of purification protocol with 0.1 M NaCl step size. A final window of 1.35–2.5 M NaCl was chosen for purification (D) Absolute monosaccharide and sulfate amount after acid hydrolysis of the optimized anion exchange protocol with elution steps of 1.35–2.5 M NaCl. The inset shows a photograph of the purified and freeze-dried mannan. Monosaccharide abbreviations: GlcA, glucuronic acid; GalA, galacturonic acid; Rha, rhamnose; Xyl, xylose; Glc, glucose; Gal, galactose; GlcN, glucosamine; Fuc, fucose; Man, mannose. Figure S3. *Polaribacter* sp. Hel1_33_49 grows on and degrades a sulfated α-mannan. The medium contained 3.5% Sea Salt as well as nitrogen, phosphate and other essential nutrients. Top: Growth curve as assessed by optical density measurements at 600 nm. Bottom: Carbohydrate-polyacrylamide gel electrophoresis of the mannan in the culture supernatant during growth stained with 0.02% Stains-all. Figure S4. The different *Polaribacter* strains remove the mannan depending on the presence of the PUL. Growth and glycan monosaccharide removal was tested with *Polaribacter* strains Hel1_33_49, _78, _96 and Hel1_88 on 0.5–5 M fractions of mannan-containing anionic exudates from *Chaetoceros affinis* (*n* = 3). After incubations the remaining glycans were acid hydrolyzed and analyzed by HPAEC-PAD. The control sample was not incubated with bacteria. The strains with the PUL metabolized the mannose of the mannan, while the Hel1_88 (negative control) without the PUL did not consume the mannose of the mannan. Figure S5. Growth and removal of the mannan by *Polaribacter* sp. Hel1_33_78. The strain was grown on the 0.5–5 M fraction of mannan-containing anionic exudates produced by *Chaetoceros affinis* and *Conticribra (Thalassiosira) weissflogii.* Supernatants were sampled during growth and the decrease of monosaccharides after acid hydrolysis was determined using HPAEC-PAD. Over time the strain removed the mannan from the culture as indicated by depletion of the detected mannose. Figure S6. Release of reducing end sugars by enzyme extracts of *Polaribacter* sp. Hel1_33_78 tested against 0.5–5 M fraction of mannan-containing anionic exudates (Man) produced by *Chaetoceros affinis* and *Conticribra (Thalassiosira) weissflogii.* Cell lysates were produced after different induction times, incubated with both substrates and analyzed using a reducing sugar assay (*n* = 3). Plot titles indicate “origin of assay substrate / origin of growth substrate.” Figure S7. Mass-spectrometry shows oligosaccharide products of PbSulf1-GH195. Digestion of the mannan using PbSulf1-GH195 analysed by Liquid Chromatography-Mass Spectrometry (LC–MS). Data show the extracted ion chromatograms of a subset of *m/z*-values corresponding to oligosaccharides found. Figure S8. Initial mannan breakdown with two endo-acting enzymes. PbSulf1-GH195 homologues OpSulf1 and OpGH195 are both required for mannan oligomerization. Reducing-end assay of enzymes from *O.* *pacifica* (*n* = 3). Figure S9. OpSulf1 crystal structure. (A) Cartoon representation of OpSulf1 with four molecules in the asymmetric unit. (B) Active site catalytic and binding residues of OpSulf1 (salmon) overlayed with PbSulf1-GH195 (turquoise) and carrageenan sulfatase from *Pseudoalteromonas* (light blue, PDB-ID: 6B0K, rmsd: 1.317 Ȧ). (C) Electrostatic surface charge representation of the active site region. Figure S10. Fragmentation patterns discriminate different positions of sulfate on mannose. Averaged and normalized fragmentation spectra from LC–MS/MS experiments with mannose-2-sulfate (A), mannose-3-sulfate (B), mannose-4-sulfate (C) and mannose-6-sulfate (D). Structures show observed cleavages, with annotations according to the nomenclature of Domon & Costello (1988). Figure S11. Comparison of fragmentation patterns confirms that digestion with PbSulf1-GH195 and GH92_1 yields mannose-6-sulfate. MS/MS spectra from fragmentation of mannose-6- sulfate (upper panels, yellow) and duplicate digests (lower panels, purple). Retention times of spectra used for averaging are in the corners. Figure S12. Quantification of sulfated α-mannan. (A) Product formation of PbSulf1-GH195 and GH92_1 follows a linear trend. Net reducing end signal is calculated as absorbance difference between digested and undigested samples. (B) *Conticribra weissflogii* biomass was extracted sequentially using hot water, EDTA or NaOH. Mannan content in dialyzed extracts were enzymatically quantified after AEX enrichment. ***Dry weight of dialyzed and dried EDTA extracts was below 0.1 mg. (C) Processing control to determine loss of mannan during the enrichment procedure with anion exchange chromatography (AEX). Defined amounts of mannan were loaded on three different AEX columns of the same type and recovery was calculated using reducing end signal after enzyme digestion (*n* = 3). FigureS13. Global abundance of the mannan polysaccharide utilization locus (PUL) from *Polaribacter sp*. Hel1_33_49 and selected reference PULs specific for other glycans in metagenome data from the *Tara* Oceans database. (A) The DNA sequence of the Mannan PUL was mapped against the raw metagenome reads of *Tara* Oceans surface water samples filtered at sizes ranging 0.2–3 μm across 45 different stations (62). Read abundances were calculated in terms of RPKM and normalized by length of the PUL and displayed as filled circles. Corresponding plots for the laminarin PUL from* Aurantivirga* MAG C_MB344 (38), the ulvan PUL from *Formosa* *agariphila* KMM 3901 T (68) and the porphyran PUL from *Zobellia galactanivorans* DsijT (63) can be found in SI. (B) Raw read abundance of the clade of *Bacillariophyta* within the V9 rDNA metabarcoding dataset from *Tara* Oceans surface water samples filtered at sizes ranging 5–20 μm across 35 stations. Please note: No diatom data were available for the remaining stations. (C-E) The sequences from the respective PUL were mapped against the raw reads from *Tara* Oceans at the respective stations. Counts were normalized by the length of the PUL and 24 are displayed as filled circles according to resulting RPKM. (C) Laminarin PUL from *Aurantivirga* MAG C_MB344. (D) Ulvan PUL from *Formosa agariphila* KMM 3901 T. (E) Porphyran PUL from *Zobellia galactanivorans* DsijT*.* Porphyran PUL was detected at 12 out of 45 stations with low read abundances. For better visibility these are displayed at lower scale with empty circles. Values ranged from 1–5 × 10–4 RPKM. Figure S14. 1H NMR of the mannan. Figure S15 1H-13C HSQC NMR of the mannan from *C. weissflogii*. Figure S16. 1H-13C HSQC NMR mannan from *C. weissflogii* displayed at higher intensity to show minor peaks. Figure S17. Stacked 1H NMR of *C. weissflogii* mannan (top) and synthetic α-1,3 6-*O*sulfated mannan (bottom). Figure S18. Superimposed 1H-13C HSQC NMRs of mannan from *C. weissflogii* (red blue) and synthetic α-1,3–6-*O*-sulfated mannan (greyscale). Figure S19. 1H-13C HSQC NMR of mannan from *C. weissflogii* with major mannose peaks labelled. Figure S20. 1H-13C HSQC NMR mannan with mannose from *C. weissflogii* (M) and possible xylose (X) branch peaks labelled. Figure S21. 1H-13C HSQC TOCSY of mannan from *C. weissflogii*. Highlighted are putative xylose cross-peaks. Figure S22. 1H-13C HSQC TOCSY of *C. weissflogii* mannan. Highlighted are mannose cross-peaks. Figure S23. 1H-1H COSY of *C. weissflogii* mannan. 4.42 ppm (H-1 xylose-like) vicinal coupling to 3.37 ppm (H-2 xylose-like). Figure S24. 1H-1H COSY of mannan. Mannose (cross-peak 4-*O*-sulfation) coupled a H-5,4.64 ppm to 3.89 ppm suggesting a minor quantity of 4-*O*-sulfation. Figure S25. 1H-13C HMBC of sulfated mannan. Figure S26. Superimposed 1H-13C HMBC (greyscale) and 1H-13C HSQC (red/blue) NMR spectra. Figure S27. Superimposed 1H-13C HMBC (greyscale) and 1H-13C HSQC (red/blue) spectra. Figure S28. Superimposed 1H-13C TOSCY (greyscale) and 1H-13C HSQC (red/blue) spectra. Figure S29. Automated assembly of mannan trisaccharide. (A) Crude NP-HPLC of automated assembly up to trisaccharide. Trace is UV 280 nm. HPLC Method 1 (20 to 55EA). (B) MALDI-TOF spectrum of mannotriose. Chemical Formula: C107H94KO26.Expected mass, 1833.5670, observed mass 1833.239. Figure S30. a NP-HPLC of automated assembly of hexasaccharide. (A) NP-HPLC of 6-mert-weiss with no-fmoc. Signal is UV 270 nm and HPLC method 2 (30 to 90EA). (B) MALDITOF of mannose-1,3-hexasacccaride. Note Fmoc protecting groups are removed. Chemical Formula: C122H124NaO38. Expected mass, 2219,7668, Observed, 2218.987. Figure S31. MALDI-TOF of mannose-1,3-hexasacccaride with ester protecting groups removed. Chemical Formula: C78H98NaO31. Expected mass, 1553,5990; Observed, 1553,223. Scheme S1. Automated glycan assembly of sulfated mannan oligosaccharide. Table S1. Comparison of the *C. weissflogii* mannan to a synthetic standard. *difference is *C. weissflogii* mannan minus synthetic glycan. Table S2. Polaribacter strains grown in HaHa 100 V medium with or without mannan containing anionic exudates from *T. w.* (0.5-5 M fraction after AEX). Table S3. Activity assays of enzymes on mannan containing anionic exudates from *T. w.* (0.5-5 M fraction after AEX), pure mannan or on synthetic substrates. n.d.: no activity detected. -/-: not tested. Table S4. Data collection and refinement statistics for OpSulf1. Table S5. Confirmation of sulfated mannose position based on retention time and MS/MS. Fragmentation ions generated from HCD fragmentation of 259.0129 m*/z* with a window of 0.4 Da. Alpha-mannan was digested with PbSUlf1-GH195 and PbGH92_1. Table S6. Primers used for cloning of PUL enzymes. Genes marked with an asterisk were cloned using Gibson-assembly into a regular pet28A vector. Data S1. Chemical Synthesis.

## Data Availability

All data are available in the manuscript or the supplementary materials.
