# Correction: Sulfated mannan of diatoms selects host-specific microbiota in the sunlit ocean

**DOI:** 10.1186/s40168-026-02451-4

**Published:** 2026-06-06

**Authors:** J. Krull, C. Sidhu, V. Solanki, M. Bligh, L. Rößler, R. K. Singh, G. Huang, C. S. Robb, H. Teeling, P. H. Seeberger, T. Schweder, C. J. Crawford, J.‑H. Hehemann

**Affiliations:** 1 Faculty of Chemistry & Biology, BIOM, University of Bremen, Bremen, Germany; 2 MARUM, University of Bremen, Bremen, Germany; 3 Max Planck Institute for Marine Microbiology, Bremen, Germany; 4 Max Planck Institute of Colloids and Interfaces, Potsdam, Germany; 5 Institute of Pharmacy, University of Greifswald, Greifswald, Germany; 6 Institute of Marine Biotechnology, Greifswald, Germany; 7 Institute for Chemistry and Biology of the Marine Environment (ICBM), School of Mathematics and Science, Carl Von Ossietzky Universität Oldenburg, Oldenburg, Germany; 8 School of Chemistry, Trinity Biomedical Sciences Institute, Trinity College Dublin, Dublin, Ireland

**Correction: Microbiome 14, 112 (2026)**

**https://doi.org/10.1186/s40168-026-02379-9**

Following publication of the original article [1], the author reported that there is a clerical error in Figure 1G. 2018 should be 2020.

The incorrect Figure is
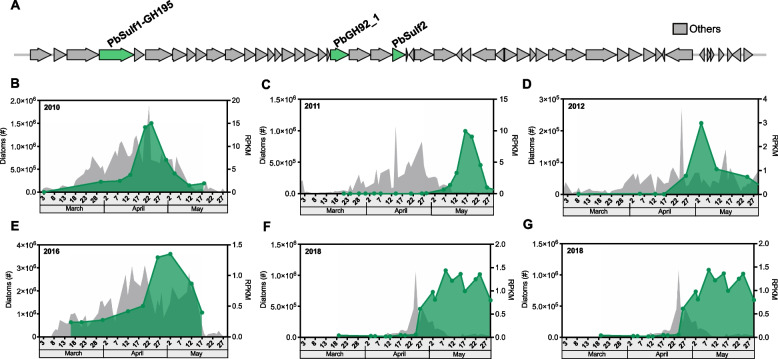


The correct Figure is
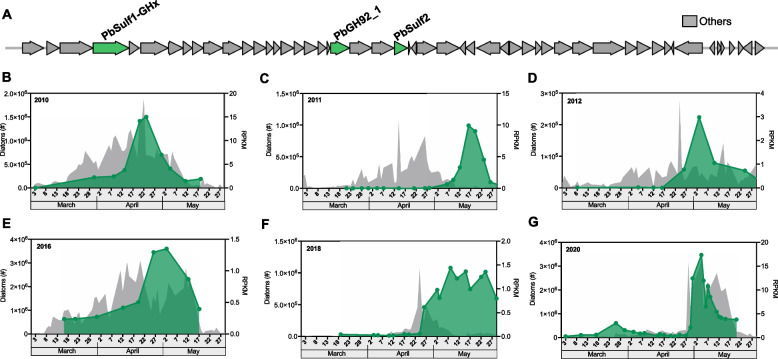


The original article has been updated.
