# Supplementary material for: Sulfated mannan of diatoms selects host-specific microbiota in the sunlit ocean
Source: Microbiome. 2026 Mar 26;14:112. doi: 10.1186/s40168-026-02379-9 (PMC13063451; doi:10.1186/s40168-026-02379-9)
Supplement: Supplementary file 2 — Supplementary Material 1: Detailed materials and methods. Supplementary text. Figure S1. (A) PUL structures in Polaribacter sp. Hel1_33_49/78/96 and Ochrovirga pacifica S85. The genes displayed in green are subject of this study. (B) Synteny plot: The Helgoland Roads dataset, comprising approximately 90 metagenomes, was manually searched for mannan PULs. For the years 2010–2018, only Illumina-based metagenomes were available; due to the short-read sequencing technology, no complete PULs could be recovered, and only truncated subsets of the mannan PUL were detected. In contrast, PacBio metagenomes sequenced in 2020 enabled the identification of a complete homolog of the mannan PUL in a Polaribacter MAG (X_MB188), corresponding to the studied Polaribacter strain (Hel1_33_49, as shown in the figure). Both PULs displayed a high level of synteny (~ 100% identity), particularly across the three previously studied and characterized enzymes (highlighted in green in the figure). The corresponding genes also exhibited a high degree of sequence conservation across all other detected PULs. Figure S2. Purification of a sulfated mannan exuded by Conticribra weissflogii. (A,C) Anion exchange chromatography of culture supernatant of C. weissflogii with stepwise increase of NaCl concentration. Total carbohydrate signals of each fraction are displayed as black dots, and stepwise NaCl elution profiles as dashed lines. (A) Step size of 0.5 M NaCl (B) Relative monosaccharide composition after acid hydrolysis of eluted fractions from analytical replicates (n = 3) (C) Optimization of purification protocol with 0.1 M NaCl step size. A final window of 1.35–2.5 M NaCl was chosen for purification (D) Absolute monosaccharide and sulfate amount after acid hydrolysis of the optimized anion exchange protocol with elution steps of 1.35–2.5 M NaCl. The inset shows a photograph of the purified and freeze-dried mannan. Monosaccharide abbreviations: GlcA, glucuronic acid; GalA, galacturonic acid; Rha, [file 40168_2026_2379_MOESM1_ESM.pdf]

Supplementary Materials for

Sulfated mannan of diatoms selects host-specific microbiota in the sunlit ocean

J. Krull[1,2,5,6], C. Sidhu[3,7], V. Solanki[3], M. Bligh[3], L. Rößler[2,3], R.K. Singh[4], G. Huang[2,3], C.S. Robb[2,3], H. Teeling[3], P.H. Seeberger[4], T. Schweder[5,6], C.J. Crawford[4,8]*, J-H. Hehemann[1,2,3]*

[1] BIOM, Faculty of Chemistry & Biology, University of Bremen, Bremen, Germany

[2] MARUM, University of Bremen, Bremen, Germany

[3] Max Planck Institute for Marine Microbiology, Bremen, Germany

[4] Max Planck Institute of Colloids and Interfaces, Potsdam, Germany

[5] Institute of Pharmacy, University of Greifswald, Greifswald, Germany

[6] Institute of Marine Biotechnology, Greifswald, Germany

[7] Institute for Chemistry and Biology of the Marine Environment (ICBM), School of Mathematics and Science, Carl von Ossietzky Universität Oldenburg, Oldenburg, Germany

[8] School of Chemistry, Trinity Biomedical Sciences Institute, Trinity College Dublin, Dublin, Ireland

Corresponding author: *conor.crawford@tcd.ie *jhhehemann@marum.de

The PDF file includes:

Detailed Materials and Methods
Supplementary Text
Figs. S1 to S31
Tables S1 to S6
Chemical Synthesis

Materials and Methods

Mannan PUL abundance in *TARA* Oceans dataset

Metagenome data of bacterioplankton during spring phytoplankton blooms was generated in previous studies from pre-filtered subsurface seawater (0.2-3 µm size fraction) sampled at the long-term ecological research site “Kabeltonne” (54° 11.3′ N, 7° 54.0′ E) at Helgoland Roads (North Sea) in the years 2010, 2011, 2012, 2016, 2018 and 2020 (Teeling *et al.*, 2016, Francis *et al.*, 2021, Wang *et al.,* 24, Sidhu et al., 2023, Reintjes 2020). For a global perspective, we used bacterioplankton metagenome data collected as part of *TARA* Oceans Expedition (TARA2017). Metagenomes from surface water samples collected across 45 different stations, filtered with sizes ranging from 0.2 to 3 µm, were used in the analysis. Mapping on the metagenomes was performed using Bowtie2 (Langmead 2012) as part of SqueezeMeta v1.6.2 pipeline (Tamames 2019). PUL abundances in terms of reads per kilobase per million mapped reads (RPKM) were calculated using the formula: (metagenome reads mapped to the PUL*10^6)/(length of PUL in kbp * total reads in a metagenome). Differences between PUL abundances were statistically assessed using the Friedmann-Test. For correlation analyses with diatom abundance, we extracted the read numbers from 25 surface water samples (5-20 µm) from eukaryotic V9 metabarcoding data (de Vargas). V9 read numbers were used as proxy for absolute diatom abundance as explained before (website reference). V9 read abundances were correlated with the corresponding PUL abundances at the respective sites. Outliers were identified for each analysis using studentized residuals and excluded prior to linear regression analyses.

Diatom culture

The centric diatom *Conticribra weissflogii*was provided by André Scheffel (Max Planck Institute for Molecular Plant Physiology, Potsdam-Golm, Germany). *C. weissflogii* was cultured in 8x20 L batch cultures with non-axenic NEPCC medium (Harrison, Waters, & Taylor, 1980) in 20 L Nalgene bottles at a constant temperature of 15 °C with a 16h/8 h light/dark cycle at an irradiance flux density of 140 μmol photons m^{-2} s^{-1}. Diatom growth was monitored by cell counting using a Neubauer counting chamber (Marienfeld, Königshofen, Germany). Cultures were inoculated using 1 L of preculture per 20 L bottle that were grown for ten days under the same conditions. Cells were separated from culture the supernatant using a continuous flow centrifuge (Thermo Fisher Scientific, Walthan, MA, USA) set at 150 mL min^{-1} and 6800 x g. The culture supernatant was filtered using a peristaltic pump through Whatman® glass microfiber filters (Grade GF/F, 0.70 µm) filters.

Preparative purification of mannan

For mannan purification, 160 L of *C. weissflogii* filtered culture supernatant were loaded onto a pre-equilibrated (50 mM Tris-HCl, 0.5 M NaCl, pH 7.5) 500 mL self-packed anion-exchange (AEX) column using ANX Sepharose 4 Fast Flow (High Sub) (Cytiva, Marlborough, MA, USA) as column material. After loading, the column was washed with 1 L buffer solution (50 mM Tris-HCl, 1.35 M NaCl, pH 7.5). Mannan was subsequently eluted with 50 mM Tris-HCl with 2.5 M NaCl, pH 7.5. The purification was carried out at a flow rate of 50 mL min^{-1}. To remove salt, eluted fractions were repeatedly concentrated and diluted in MilliQ water (Merck KGaA, Darmstadt, Germany) over 30 kDa molecular weight cutoff membranes using an Amicon ultrafiltration stirred cell (Merck Millipore, Burlington, MA; USA) until the theoretical NaCl concentration was below 1 µM. The desalted sample was freeze-dried. For fractionation of the anionic high molecular weight DOM the same method was applied but instead of 1.35 and 2.5 M NaCl, 0.5 and 5 M NaCl were used. The dried compounds were then resuspended and loaded on a pre-packed 5 mL ANX column using ANX Sepharose 4 Fast Flow (High Sub) resin as column material (Cytiva). The column was equilibrated with 50 mM Tris-HCl pH 7.5 and for elution a stepwise elution with 0.5 M NaCl increments was applied up to 5 M NaCl. Per step, 15 mL of sample were collected. The samples were exhaustively dialyzed against MilliQ water (Merck) and freeze-dried prior to monosaccharide composition and sulfate analysis.

Glycan monosaccharide composition and sulfate content analyses

Desalted freeze-dried mannan obtained after anion exchange chromatography was dissolved in MilliQ water (Merck) to a concentration of 1 mg mL^{-1} and hydrolyzed using 1 M HCl at 100 °C for 24 h in pre-combusted glass vials. To remove HCl, samples were either vacuum centrifuged or diluted 1:1000 in MilliQ water (Merck) prior to further analyses. For monosaccharide measurements, we adapted a protocol for determination of neutral, amino- and acidic sugars using high performance anion exchange chromatography (HPAEC) described previously (Engel & Händel, 2011). In short, a Dionex ICS-5000+ system (Thermo Fisher Scientific) with pulsed amperometric detection (PAD) was equipped with a CarboPac PA10 analytical column (2 × 250 mm) and a CarboPac PA10 guard column (2 × 50 mm). System control and data acquisition were performed with Chromeleon v7.2 software (Thermo Fisher Scientific). Neutral and amino-monosaccharides were separated by an isocratic flow of 18 mM NaOH for 20 min, followed by a gradient up to 200 mM sodium acetate over 10 min to separate acidic sugars. Monosaccharide standards were used to identify peaks by retention time and a standard mix ranging from 1–10 to 1000 µg L^{-1} was used to quantify the amount of monosaccharide (x-axis amount and y-axis peak area). The released sulfate from acid-hydrolyzed sulfated α-mannan was measured on a Metrohm 761 compact ion chromatograph (Metrohm, Herisau, Switzerland) with a Metrosep A Supp 5 column and suppressed conductivity detection with 0.5 M H_2SO_4. Ions were separated by an isocratic

flow of carbonate buffer (3.2 mM Na_2CO_3 and 1 mM $NaHCO_3$) and the duration of a run was 20 min, with sulfate eluting at 16 min. Chromatograms were analyzed with the instrument's software MagIC Net version 3.2. All samples for monosaccharide and sulfate composition were prepared in triplicates.

Nuclear magnetic resonance and chemical synthesis of oligosaccharides

All chemicals used were reagent grade and used as supplied unless otherwise noted. A detailed description of the structural elucidation and glycan synthesis processes is provided in extended data. The automated syntheses were performed on a custom-built synthesizer developed at the Max Planck Institute of Colloids and Interfaces (Potsdam-Golm, Germany). Analytical thin-layer chromatography (TLC) was performed on Merck silica gel 60 F254 plates (0.25 mm). Compounds were visualized by UV irradiation or dipping the plate in a 5% H_2SO_4 ethanol solution. Flash column chromatography was carried out on an automated Grace flash chromatography system (Grace, Columbia, MD, USA). Analysis and purification by normal and reverse phase HPLC were performed using an Agilent 1200 series instrument (Agilent Technologies, Santa Clara, CA, USA). Products were lyophilized using a Christ Alpha 2-4 LD plus freeze dryer (Martin Christ GmbH, Osterode am Harz, Germany). ^{1}H-, ^{13}C- and HSQC-NMR spectra were recorded on a Varian 400-MR (400 MHz), Varian 600MR (600 MHz), or Bruker Biospin AVANCE700 (700 MHz) spectrometer (Varian, Palo Alto, CA, USA and Bruker Corp., Billerica, MA, USA). Signals were reported in terms of chemical shift [δ in parts per million (ppm)] relative to tetramethylsilane (TMS) or in D_2O using the solvent as the internal standard in ^{1}H-NMR (D_2O: 4.79 ppm ^{1}H) as the internal standard. NMR data is presented as follows: Chemical shift, multiplicity (s = singlet, d = doublet, t = triplet, dd = doublet of doublet, m = multiplet and/or multiple resonances), coupling constant in Hertz (Hz), integration. All NMR signals were assigned on the basis of ^{1}H-NMR, ^{13}C-NMR, COSY, TOCSY and HSQC experiments. ‡Carbon assignments were extracted from HSQC experiments. High resolution mass spectra were obtained using a 6210 ESI-TOF mass spectrometer (Agilent) and a MALDI-TOF autoflex instrument (Bruker). MALDI and ESI mass spectra were run on IonSpec Ultima instruments (IonSpec, Lake Forest, CA, USA). The matrix used for MALDI-MS was 2,5-dihydroxy-benzoic acid (DHB).

Cloning of plasmids

Genes were cloned using the USER cloning or Gibson assembly methodologies (New England Biolabs, Ipswich, NE, USA) with genomic DNA from *Polaribacter* sp. Hel1_33_78 (strain provided by Jens Harder, Max Planck Institute for Marine Microbiology, Bremen, Germany). All primer pairs are provided in supplementary table S2. USER cloning constructs were cloned into a modified version of the pet28A vector promoter sequence as suggested by (Shilling 2020). Cloning of the modified vector backbone was achieved by ligating two PCR reactions of the empty pet28A Vector using the primer pairs

pet28a_P1 and pet28a_P2 via the USER cloning method. Generally, all signal peptides were predicted using SignalP5.0 (Armenteros 2019) and removed before cloning. PCR reactions were done with 30 cycles (denaturation: 95 °C, annealing: 55 °C, elongation: 72 °C) using 0.25 U of enzyme in a total reaction mix of 25 µl. DNA template was digested with DpnI and PCR products were purified using a commercially available PCR purification kit (Qiagen, Hilden, Germany). The USER reaction was carried out in a one pot reaction using 0.25 U of USER enzyme and a backbone:insert ratio of 0.015 pmol : 0.15 pmol in CutSmart buffer (New England Biolabs) in a total volume of 10 µL. The reaction was incubated at 37 °C for 20 minutes followed by 20 minutes at 25 °C and directly used for transformation into chemocompetent *E. coli* NEB5α (New England Biolabs). Plasmids from transformed colonies were isolated using a commercial kit for plasmid preparation (Qiagen). All clones were verified by DNA sequencing. As the isolated *Polaribacter* spp. Hel1_33_49/78/96 does not grow on plates and were kept in cultivation since their initial isolation (Hahnke), we re-sequenced their genomes. The obtained constructs either aligned with the original or the re-sequenced genomic data of *Polaribacter* sp. Hel1_33_78. Re-sequenced genomic data of *Polaribacter* sp. Hel1_33_49 indicated highest purity of this strain. Owing to the genome identity of all strains (ANI > 98%), we used this strain for physiological experiments.

Recombinant enzyme expression and purification

Plasmids were transformed into *E. coli* BL21 (DE3) and expressed in 1 L lysogeny broth (LB) medium containing 50 µg mL^{-1} Kanamycin. The culture was grown at 37 °C under shaking until early exponential phase (OD_{600nm} 0.3-0.5). Recombinant expression was induced after cooling to 12 °C using 0.5 mM isopropyl-β-D-1-thiogalactopyranoside (IPTG) and further incubation at 12 °C for ~40 h. In the case of sulfatases, the formylglycine generating enzyme plasmid (Addgene plasmid repository, Watertown, MA, USA) (Carrico 2007) was co-transformed into the same expression host and expression was carried out as explained above except that cultures were additionally supplemented with 100 µg mL^{-1} Ampicillin and 0.02% L-arabinose was added right before cooling to 12 °C. Cells were collected by centrifugation (10,000 g, 4 °C, 45 min) and the cell pellets were stored at −20 °C. Cell lysis and purification were carried out as described previously (Solanki 2022) with the following exceptions: PbSulf1-GH195, PbGH92_1 and OpSulf1 were concentrated to 5 mg mL^{-1}, 4 mg mL^{-1} and 3.16 mg mL^{-1}, respectively. PbSulf2 was purified using a Talon column (Cytiva) without subsequent size exclusion and concentrated to 5 mg mL^{-1}. OpGH195 was only purified with IMAC and concentrated to 0.5 mg mL^{-1}. For preliminary enzyme screening experiments one spatula of cell pellet were lyzed using BugBuster (Merck) according to manufacturer's instructions and cell debris was removed by centrifugation. Cell lysates were directly used for activity testing.

Enzyme activity

All digestions were carried out in 10 mM MOPS pH 7.5 and 500 mM NaCl with 0.1% (w/v) of mannan and enzymes were added in excess (0.01-0.1 mg mL^{-1}). In preliminary screening experiments 3.5% Sea Salts (Sigma Aldrich, Burlington, MA, USA) were used instead of NaCl and lysates were added at 10% (v/v). In screening experiments with para-nitrophenylsubstrates, 1 mM per substrate were added. After overnight digestion, assays were heat inactivated at 95 °C for 5 min and centrifuged to remove precipitated proteins or cell debris. Supernatants were analyzed using fluorophore-assisted carbohydrate electrophoresis (FACE) with carbohydrates labeled with 2-aminoacridone as described in (Hehemann et al., 2010). Reducing-end measurements were carried out using PAHBAH as detection reagent as described previously (Stefan Becker & Hehemann, 2018). The release of sulfate and mannose from enzymatic reactions were measured using ion chromatography and HPAEC-PAD as described above, respectively. All enzymatic assays were carried out as technical replicates in triplicates unless otherwise stated.

Crystallization, data collection, structure solution, refinement and AlphaFold2

OpSulf1 solution of 3.16 mg/mL was used for initial crystallization attempts. Crystallization was performed in two drop 96-well crystallization plates in sitting drop format using commercial crystallization screens. Plates were incubated at 16 ºC. Start of the crystal formation was observed within 2-3 weeks. Diffraction quality crystals were obtained using 20% (w/v) polyethylene glycol 3,350, 200 mM potassium chloride. Crystals of the OpSulf1 required cryo-protection in crystallization solution supplemented with 20% glycerol prior to flash cooling in a liquid nitrogen at 100 K. Diffraction data was collected on DESY beamline P11. A total of 3600 images were collected (exposure: 0.1 s; oscillation: 0.1º). Measured reflections intensities were indexed, integrated and scaled using the autoproc pipeline available at the beamline facility. The OpSulf1 structure was solved to 1.9 Å using molecular replacement with the AlphaFold2 predicted model as reference in PHASER (McCoy 2007). For automatic model building, BUCCANEER (Cowtan 2006) was used. Refinement of the initial models was done in REFMAC5 (Murshudov 2011) and further rounds of manual model building were carried out using COOT (Emsley 2010). Both models and structure factors for OpSulf_1 were deposited in the Protein Data Bank (PDB) with accession 9FVT. Corresponding data-processing and refinement statistics are summarized in Table S4. The structural comparison of OpSulf with homologs were performed using the PyMOL v.2.3.2 (Schrödinger, New York, NY, USA). Refinement data can be found in Table S4. For enzymes that did not crystallize we predicted using AlphaFold2 (Jumper 2021). All structures had predicted Local Distance Distance Test Values (pLDDT) of: PbSulf1-GH195_1 (pIDDT=91.2), PbGH92_1 (pIDDT=94.9), PbSulf2 (pIDDT=93.6) and OpGH195 (pIDDT=94.2).

LC-MS of mannose-sulfate and mannan digests

Standards and digests were diluted in 90% UHPLC-grade and 10% (v/v) MilliQ water (Merck) for LC-MS measurements. 5 µL were injected to an Accucore-150-amide HILIC

column (Thermo Fisher Scientific) held at 60 °C on a Thermo Fisher Vanquish Horizon UHPLC coupled to a Thermo Fisher Q-Exactive Plus MS. Mobile phases A and B consisted of 10 mM ammonium formate at pH 5 and UHPLC-grade acetonitrile, respectively. A gradient started after 1 min at 90% B and decreased from 90% to 40% B over 40 min at a flow rate of 0.4 mL min^{-1}. The column was equilibrated at 90% for 16 min at the end of each run. Mono- and oligosaccharides was detected in negative mode with the following MS conditions: spray voltage, 2.5 kV; sheath, auxiliary and sweep gas flow rates, 50, 13 and 3 respectively; capillary temperature, 260 °C; S-lens RF level, 60; auxiliary gas heater temperature, 425 °C; resolution, 70,000 at 200 *m/z*; scan range, 175 to 1,400 *m/z*, maximum IT, 100 ms; AGC target, 1E6. Mannose standards (synthetic or commercial) sulfated at various positions (2, 3, 4, 6) were also analyzed with LC-MS/MS with data-dependent acquisition. The *m/z* value of the $[M-H]^-$ ion (259.013) was provided in an inclusion list. MS/MS settings: resolution, 17,500; AGC target, 1E5; maximum IT, 50 ms; loop count, 3; isolation window, 0.4 *m/z*; normalized collision energy (NCE), 40. The intensity threshold was 1E5 and dynamic exclusion was applied for 10 s. A targeted selected ion monitoring (tSIM) method was used for oligosaccharides yielded by GH99 digestion of alpha-mannan. Target ions identified in preliminary analyses were provided as a timed inclusion list: 259.0131 *m/z*, 1-4 min; 421.0658 *m/z*, 8-10 min; 250.0078 *m/z*, 11-12 min; 377.0853 *m/z*, 6-11 min; 583.1192 *m/z*, 11-13 min; 331.0337 *m/z*, 12.5-14 min; 452.0390 *m/z*, 16-18 min; 573.0444 *m/z*, 19-21 min; 694.0494 *m/z*, 21-23 min; 543.0338 *m/z*, 23-25 min. SIM settings were as follows: resolution, 35,000 at 200 *m/z*; AGC target 5E4; maximum IT, 100 ms; isolation window, 0.4 *m/z*. Thermo Fisher RAW files converted to mzML format using the ProteoWizard 'msconvert' version 3.0.20239 with a 'peakPicking' filter (Chambers et al., 2012). ‘XCMS’ (v3.16.1) was used to import data into R (R core team, 2021) and extract ion chromatograms (Smith et al., 2006).

Bacterial growth

Initial growth experiments with *Polaribacter* spp. Hel1_33_49/78/96 and Hel1_88 were carried out in HaHa-medium supplemented with 0.1 mg mL^{-1} yeast extract and 2 mg mL^{-1} unpurified mannan eluted from the AEX column between 0.5-5M NaCl from either *C. weissflogii* or *C. affinis* (Hahnke & Harder 2013). Growth was assessed using by measuring the optical density at 600 nm. Additionally, monosaccharide compositions after acid hydrolysis of the culture supernatant were analyzed as described above. *Polaribacter* sp. Hel1_33_78 grown on both substrates were harvested by centrifugation and the pellets were lysed using BugBuster (Merck) according to manufacturer’s protocol on glucose. Lysates were tested for cross-reactivity on substrates from both, *C. weissflogii* and *C. affinis* mannan. Digests were incubated overnight using 3.5% Sea Salts (Sigma), 10 mM MOPS pH 7.5 and 0.5 mg mL^{-1} mannan (0.5-5 M NaCl fraction) and analyzed using carbohydrate-polyacrylamide gel electrophoresis (C-PAGE). C-PAGE analysis was carried out as described in (Robb 2017) except that stacking and resolving gels were prepared with 10% and 25% acrylamide:bisacrylamide (19:1 v/v), respectively. *Polaribacter* sp. Hel1_33_49 was grown in 3.5% Sea Salts (Sigma) supplemented with 0.03% casaminoacids, 1x 7-vitamin and 1x trace elements solutions and 26 mg L^{-1} of ammonium ferric citrate. Trace elements and 7-vitamin solutions were prepared as 1000x

stocks as described elsewhere (Hahnke & Harder, 2013). The mannan was used as limiting carbon source at a concentration of 1 mg mL^{-1}. Growth was determined by measuring the absorbance at 600 nm. Growth curves were plotted with a logistic growth function using the non-linear least squares method. Substrate consumption was verified qualitatively using C-PAGE.

Enzymatic mannan quantification

Cultures of *Conticribra weissflogii*were grown in NEPCC medium as described above. Subsamples of 200 mL of culture were filtered using GF/F filters and loaded on equilibrated pre-packed 1 mL ANX columns using ANX Sepharose 4 Fast Flow (High Sub) (Cytiva). Columns were equilibrated with wash buffer containing 500 mM NaCl and 50 mM Tris-HCl pH7.5. After loading, the columns were washed with the same buffer buffer. Elution was done using a syringe containing 1.7 mL of elution buffer (5 M NaCl, 50 mM Tris-HCl pH 7.5). After syringe mounting, 200 µL of eluate were discarded and the following 1.5 mL were collected and desalted in 2 mL MilliQ water (Merck) using a desalting column according to manufacturer's instructions (Cytiva). Desalted samples were split in halves and dried using a vacuum centrifuge at 30 °C. One of each sample was digested in 20 µL using PbSulf1-GH195 and PbGH92_1 as described above but with 3.5% Sea Salts (Sigma Aldrich). The second sample served as negative control. The digestion volume was 20 µL which were subsequently analyzed using PAHBAH. If necessary, PAHBAH reactions were diluted tenfold before measurement. For data analysis, negative control absorbances were subtracted from digestions. Results were compared with a standard curve containing defined amounts of mannan. The procedure recovery was determined to be at 80 ± 5% (n=3) compared to untreated mannan **(fig S7C)**.

Extraction of mannan from cellular biomass

After diatom cultivation the cell pellet was freeze-dried and sequentially extracted using MilliQ water (Merck), 0.4 M EDTA (pH 8) and 4 M NaOH with 0.1% NaBH4 (Vidal-Melgosa 2021). For 1 g of biomass 20 ml of sample were used. In each extraction step the sample was sonicated and centrifuged at 14.000 x g for 10 minutes. NaOH extracts were neutralized to pH 7.5 using HCl and diluted in MilliQ to < 500 mM NaCl. The mannan content in all extracts was determined as described above.

Detection of mannan from other diatoms

The diatom strains *Thalassiosira pseudonana*, *Chaetoceros affinis* and *Thalassiosira rotula* were cultivated as described above. 20 L of culture supernatant were concentrated after filtration using GF/F. For concentration, tangential ultrafiltration (Sartorius, Göttingen, Germany) with three cassettes of 30 kDa cutoff membranes (3051465901E–SG, Sartocon Slice PESU Cassettes, Sartorius Stedim) filters was applied resulting in

0.5 L concentrate. For dialysis, 20 L MilliQ water (Merck) were applied after sample loading. The dialyzed concentrate was loaded on a 500 mL anion exchange column as described above. The washing and elution buffers had 50 mM Tris-HCl NaCl pH 7.5 and contained 0.5 M and 5 M NaCl, respectively. The dialyzed and freeze-dried samples were used as substrates for enzymatic assays using PbSulf1-GH195_1 and PbGH92_1 as described above but instead of NaCl, 35 mg mL^{-1} Sea Salts (Sigma Aldrich) were used in the digestion. Digested samples were analyzed with FACE as described above.

Supplementary text

NMR analysis of *C. weissflogii* mannan

To establish the *C. weissflogii* mannan polysaccharide primary structure and establish connectivity, ^{1}H **(Fig. S12)**, ^{1}H-^{13}C HSQC **(Fig. S15-16** and **S19-20)**, ^{1}H-^{1}H COSY **(Fig. S23-24)**, ^{1}H-^{13}C HMBC **(Fig. S25-27)** and HSQC-TOCSY (SI Figure 21 and 22) at 700 MHz were collected at 20 °C. Monosaccharide composition and NMR analysis indicated the presence of two monosaccharides **(Fig. S15-16)**. The major component was an α-1,3-D-mannan with the majority of the C6 carbon de-shielded to 67.5ppm, suggesting 6-*O*-sulfation **(Fig. S15)**. The second less abundant monosaccharide appears to be β-D-xylose due to the characteristic splitting of the C-5 axial and equatorial protons (SI Figure 20). A single peak in the glycan region of the NMR at 5.46 and 99.47 ppm was not assigned to be part of the polysaccharides structure (SI Figure 4 and 7). This single peak in ^{1}H-^{1}H COSY had no vicinal coupling to other protons in the spectra, additionally, it did appear to be a part of any spin system, with no cross-peaks appearing in the HSQC-TOCSY experiment **(Fig. S22)**. Inter-residue linkage connectivity was determined via a HMBC with a correlation peak between the possible anomeric xylose proton and a C6 carbon at 4.43 and 68.1 ppm **(Fig. S26)**. Suggesting, that xylose residues could exist as β-1,6 branches to the α-mannan backbone. A minor peak at 4.62 and 75.9 ppm was characteristic of 4-*O*-sulfation **(Fig. S24)**, and using ^{1}H-^{1}H COSY it was found to be connected to a non-sulfated 6-OH group **(Fig. S20)**. In summary, the major component of the *C. weissflogii* polysaccharide was hypothesized to be an α-1,3-mannan with a high degree of 6-*O*-sulfation. To verify this proposed structure, we synthesised defined standards of the structure, one sulfated and one non-sulfated α-1,3-mannan oligosaccharide.

Automated synthesis of sulfated mannan oligosaccharides

The automated synthesis of the sulfated α-1,3 mannan backbone required a single building block. Initially an ethyl 2-*O*-benzoyl-4-*O*-benzyl-3-*O*-(9-fluorenylmethoxycarbonyl)-6-*O*-levulinoyl-1-thio-α-D-mannopyranoside building block was tested. The choice of the 9-fluorenylmethoxycarbonyl (Fmoc) as a temporary protecting group at the 3-OH would allow for fast extension of the mannan backbone,

while the 6-*O*-levulinoyl (Lev) ester would allow for chemoselective 6-OH sulfation. However, automated glycan assembly (AGA) with this building block led to unsuccessful syntheses with deletion sequences and *m/z* that could not be accurately assigned to any particular structure. These altered *m/z* can be observed when target oligosaccharides contain Lev esters (Zhu 2021). These altered *m/z* are hypothesised to originate from unwanted side-reactions of the Lev ester. To circumvent this issue, we swapped the positions of the 6-OH and 3-OH temporary protecting groups, so that the Lev esters would be now employed for backbone assembly and Fmoc groups were used to mask hydroxyl groups destined for sulfation.

This ethyl 2-*O*-benzoyl-4-*O*-benzyl-6-*O*-(9-fluorenylmethoxycarbonyl)-3-*O*-levulinoyl-1-thio-α-D-mannopyranoside (**4**) building block was allowed AGA up to 3-mers **(Fig. S29)**. However, larger oligosaccharide sequences resulted in deletion sequences as analyzed by high-pressure liquid chromatography (HPLC) and matrix-assisted laser ionization time-of-flight (MALDI-TOF) mass spectrometry (MS). Even double glycosylation cycles with thioglycoside building block **4** did not improve coupling efficiency. The low nucleophilicity of the 3-OH mannose acceptor or non-ideal temperature selection of the glycosylation module could be the origin of this poor efficiency (Tuck 2022, van Hengst 2023). The thioglycoside donor **4** was therefore converted into a dibutyl phosphate donor **1** (Plante 1999). The dibutyl phosphate block allowed the automated assembly of the desired target hexasaccharides but required the use of double cycles after the 3-mer stage **(Fig. S30)**. On resin sulfation was performed using a sulfur trioxide pyridine complex in a sealed microwave vial (SO_3.Py, 10eq. per OH, DMF, 50 °C, 16 h) with the sulfation stage tracked by Q-TOF MS analysis of microcleavage samples (Crawford 2024). On resin methanolysis (5% solution of 0.5 M NaOMe in THF) removed the remaining *O*-ester protecting groups with incubation times of 72 hours required for the sulfated α-mannan, and 16 hours for the non-sulfated mannan **(Fig. S31)**. The resin bound glycans were released from the solid-support using a LED lamp (385 nm, DMF, 16 h) with the crude photocleavage products subjected to palladium-catalyzed hydrogenolysis. Purification by reverse phase HPLC produced sulfated oligosaccharide **2** (0.7 mg) and non-sulfated oligosaccharide **3** (0.9 mg), in yields of 2.3% and 4.5% respectively.

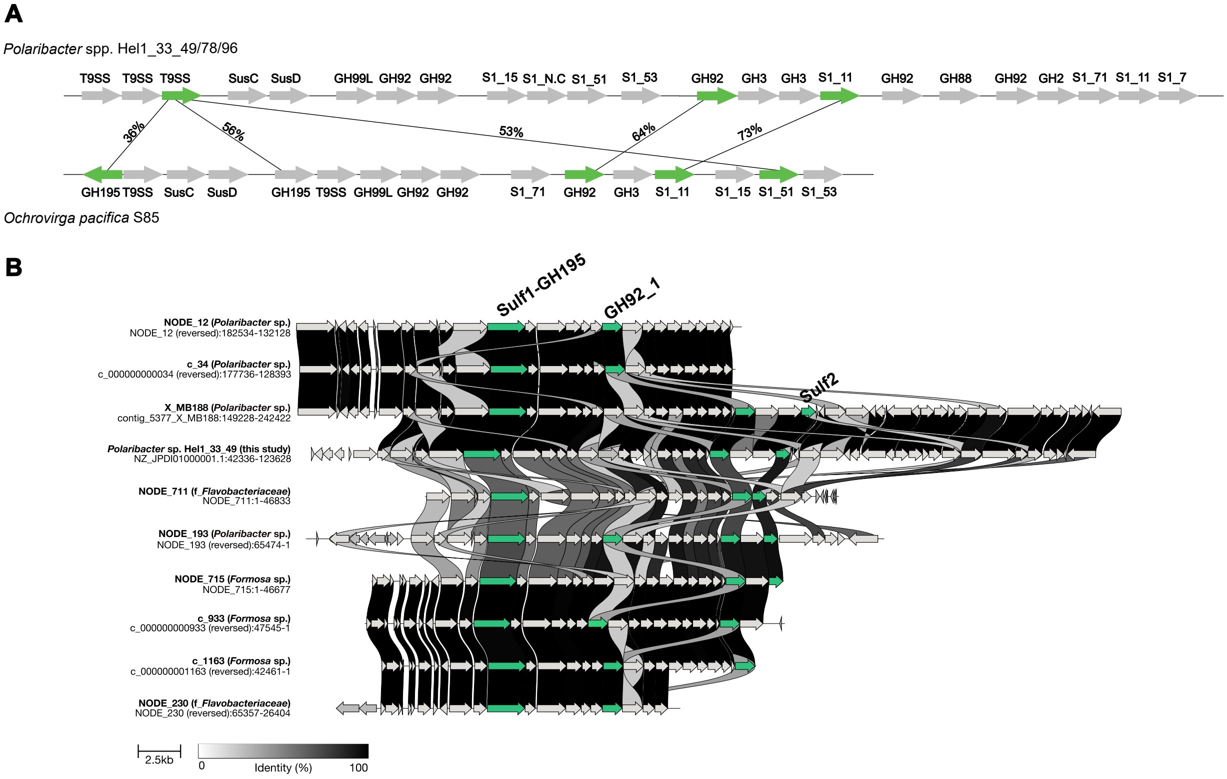

Fig. S1.

(**A**) PUL structures in *Polaribacter* sp. Hel1_33_49/78/96 and *Ochrovirga pacifica* S85. The genes displayed in green are subject of this study. (**B**) Synteny plot: The Helgoland Roads dataset, comprising approximately 90 metagenomes, was manually searched for mannan PULs. For the years 2010-2018, only Illumina-based metagenomes were available; due to the short-read sequencing technology, no complete PULs could be recovered, and only truncated subsets of the mannan PUL were detected. In contrast, PacBio metagenomes sequenced in 2020 enabled the identification of a complete homolog of the mannan PUL in a Polaribacter MAG (X_MB188), corresponding to the studied *Polaribacter* strain (Hel1_33_49, as shown in the figure). Both PULs displayed a high level of synteny (~100% identity), particularly across the three previously studied and characterized enzymes (highlighted in green in the figure). The corresponding genes also exhibited a high degree of sequence conservation across all other detected PULs.

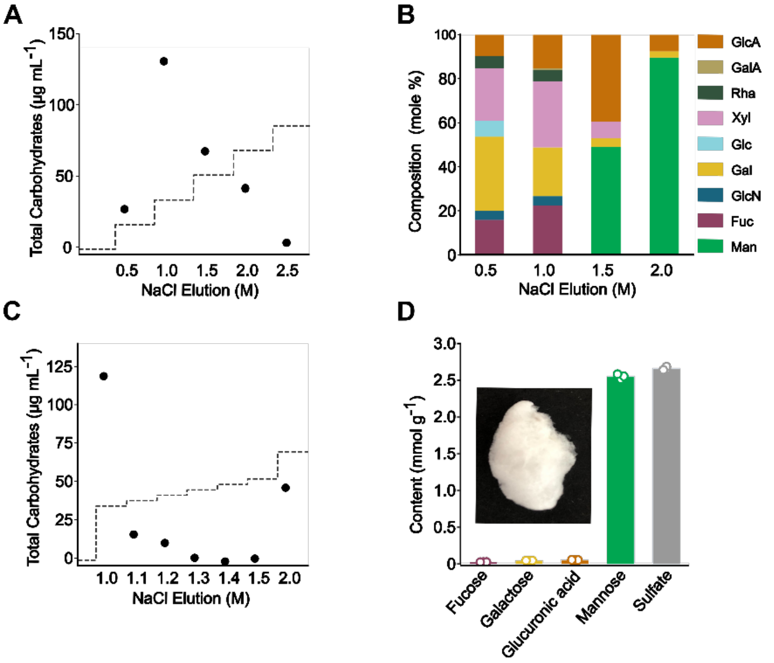

Fig. S2.

Purification of a sulfated mannan exuded by *Conticribra weissflogii***.** (**A**,**C**) Anion exchange chromatography of culture supernatant of *C. weissflogii* with stepwise increase of NaCl concentration. Total carbohydrate signals of each fraction are displayed as black dots, and stepwise NaCl elution profiles as dashed lines. (**A**) Step size of 0.5 M NaCl (**B**) Relative monosaccharide composition after acid hydrolysis of eluted fractions from analytical replicates (n=3) (**C**) Optimization of purification protocol with 0.1 M NaCl step size. A final window of 1.35-2.5 M NaCl was chosen for purification (**D**) Absolute monosaccharide and sulfate amount after acid hydrolysis of the optimized anion exchange protocol with elution steps of 1.35-2.5 M NaCl. The inset shows a photograph of the purified and freeze-dried mannan. Monosaccharide abbreviations: GlcA, glucuronic acid; GalA, galacturonic acid; Rha, rhamnose; Xyl, xylose; Glc, glucose; Gal, galactose; GlcN, glucosamine; Fuc, fucose; Man, mannose.

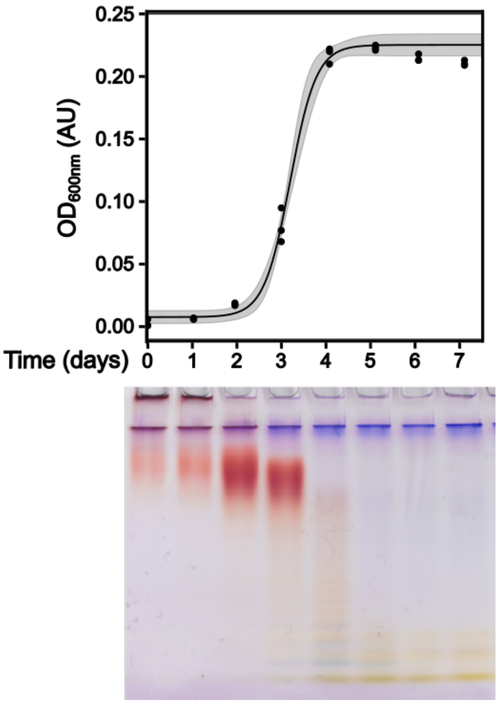

Fig. S3.

Polaribacter sp. Hel1_33_49 grows on and degrades a sulfated α-mannan. The medium contained 3.5% Sea Salt as well as nitrogen, phosphate and other essential nutrients. Top: Growth curve as assessed by optical density measurements at 600 nm. Bottom: Carbohydrate-polyacrylamide gel electrophoresis of the mannan in the culture supernatant during growth stained with 0.02% Stains-all.

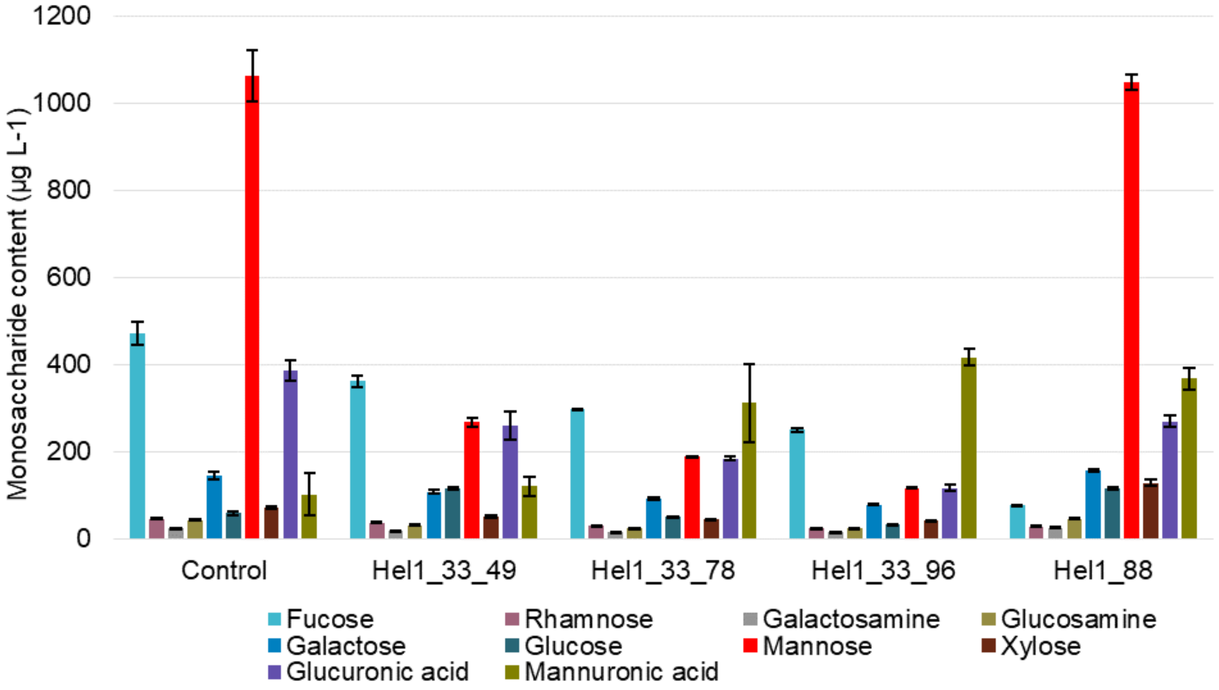

Fig. S4.

The different Polaribacter strains remove the mannan depending on the presence of the PUL. Growth and glycan, monosaccharide removal was tested with *Polaribacter* strains Hel1_33_49, _78, _96 and Hel1_88 on 0.5-5 M fractions of mannan-containing anionic exudates from *Chaetoceros affinis* (n=3). After incubations the remaining glycans were acid hydrolyzed and analyzed by HPAEC-PAD. The control sample was not incubated with bacteria. The strains with the PUL metabolized the mannose of the mannan, while the Hel1_88 (negative control) without the PUL did not consume the mannose of the mannan.

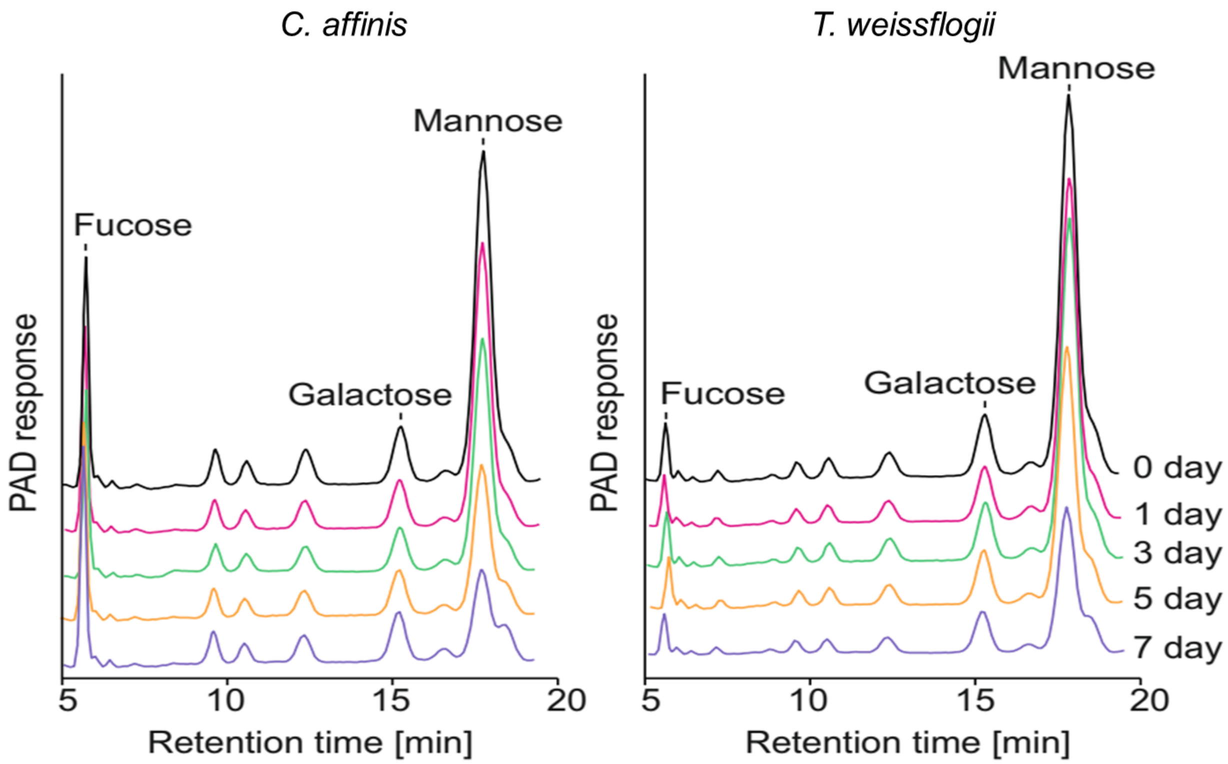

Fig. S5.

Growth and removal of the mannan by *Polaribacter* sp. Hel1_33_78. The strain was grown on the 0.5-5 M fraction of mannan-containing anionic exudates produced by *Chaetoceros affinis* and *Conticribra (Thalassiosira) weissflogii.* Supernatants were sampled during growth and the decrease of monosaccharides after acid hydrolysis was determined using HPAEC-PAD. Over time the strain removed the mannan from the culture as indicated by depletion of the detected mannose.

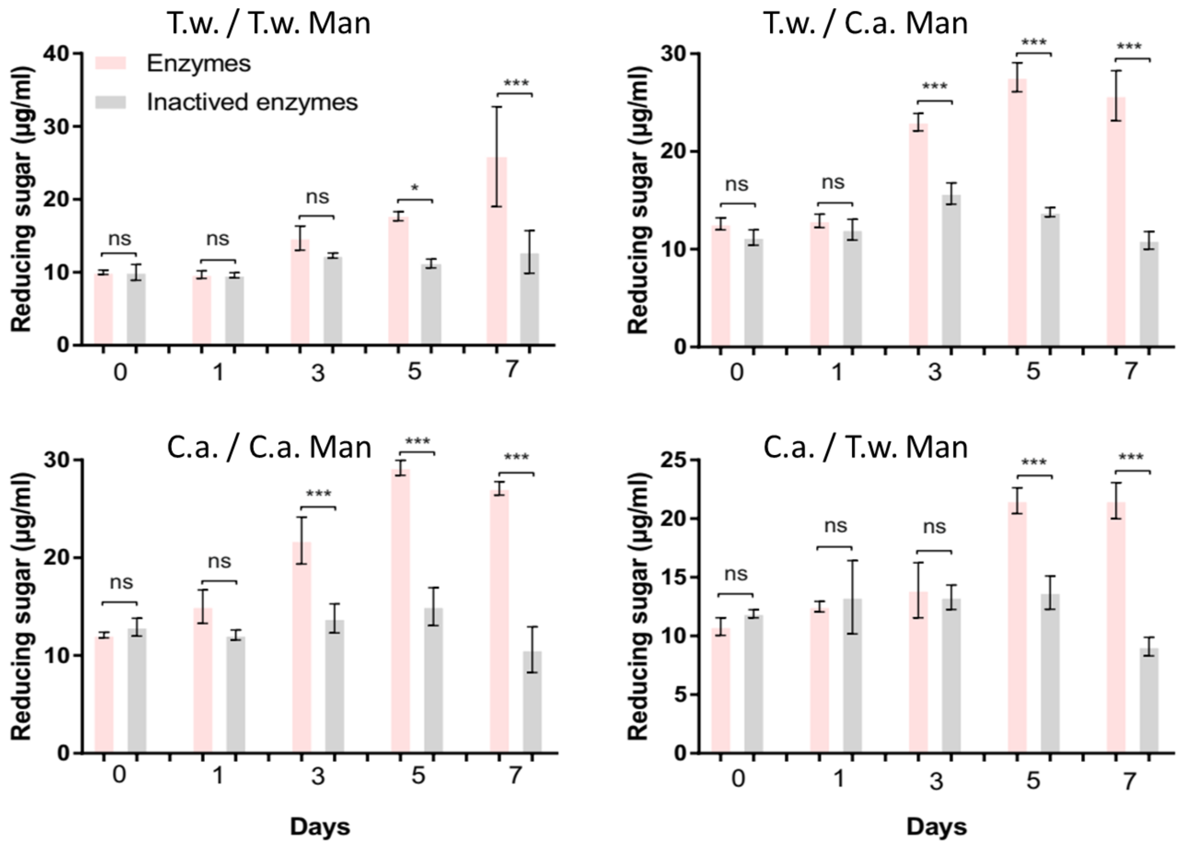

Fig. S6.

Release of reducing end sugars by enzyme extracts of *Polaribacter* sp. Hel1_33_78 tested against 0.5-5 M fraction of mannan-containing anionic exudates (Man) produced by *Chaetoceros affinis* and *Conticribra (Thalassiosira) weissflogii.* Cell lysates were produced after different induction times, incubated with both substrates and analyzed using a reducing sugar assay (n=3). Plot titles indicate “origin of assay substrate / origin of growth substrate.”

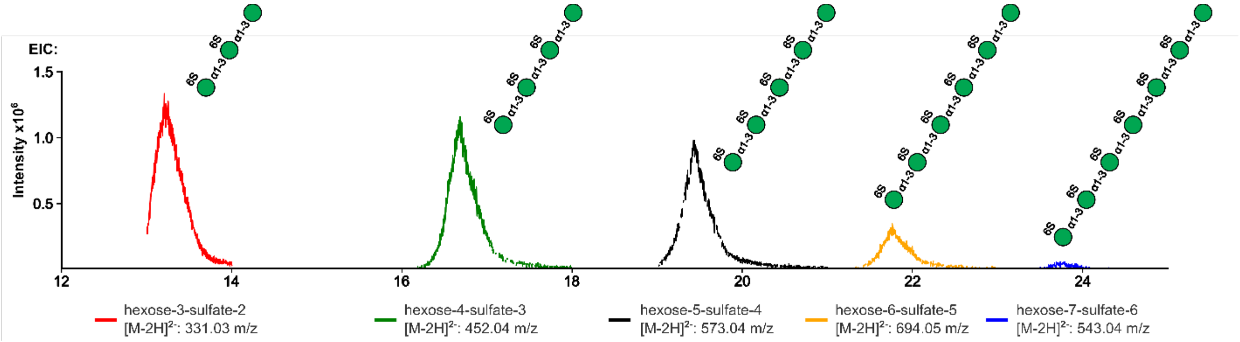

Fig. S7.

Mass-spectrometry shows oligosaccharide products of PbSulf1-GH195**.** Digestion of the mannan using PbSulf1-GH195 analysed by Liquid Chromatography-Mass Spectrometry (LC-MS). Data show the extracted ion chromatograms of a subset of *m/z*-values corresponding to oligosaccharides found.

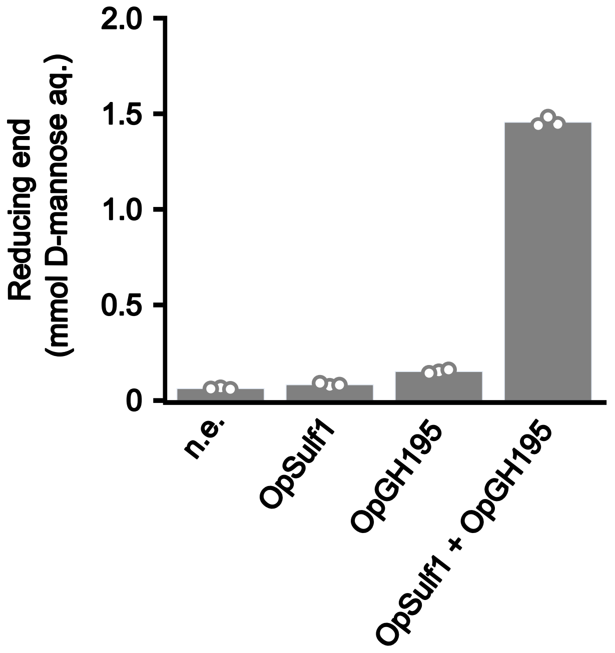

Fig. S8.

Initial mannan breakdown with two endo-acting enzymes. PbSulf1-GH195 homologues OpSulf1 and OpGH195 are both required for mannan oligomerization. Reducing-end assay of enzymes from *O. pacifica* (n=3).

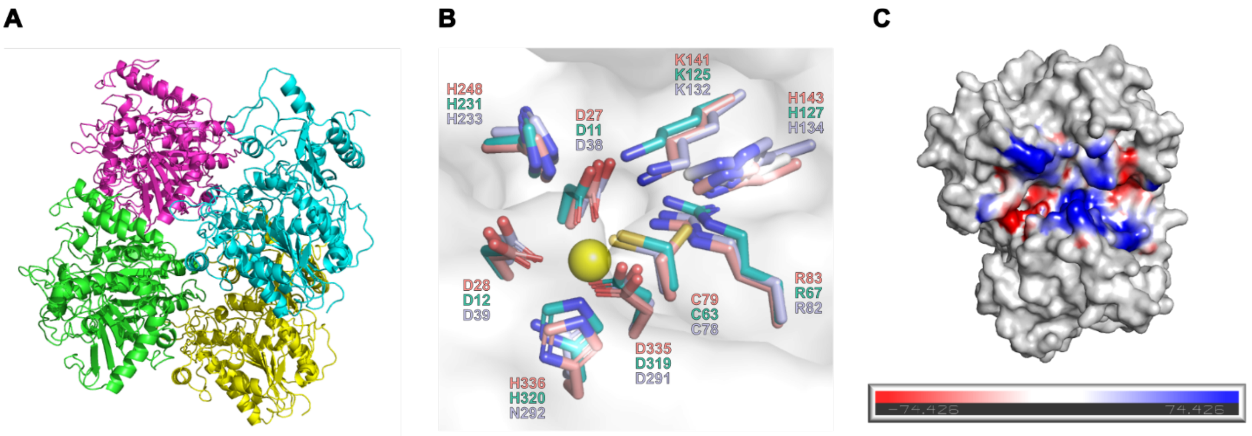

Fig. S9.

OpSulf1 crystal structure**.** (**A**) Cartoon representation of OpSulf1 with four molecules in the asymmetric unit. (**B**) Active site catalytic and binding residues of OpSulf1 (salmon) overlayed with PbSulf1-GH195 (turquoise) and carrageenan sulfatase from *Pseudoalteromonas* (light blue, PDB-ID: 6B0K, rmsd: 1.317 Å). (**C**) Electrostatic surface charge representation of the active site region.

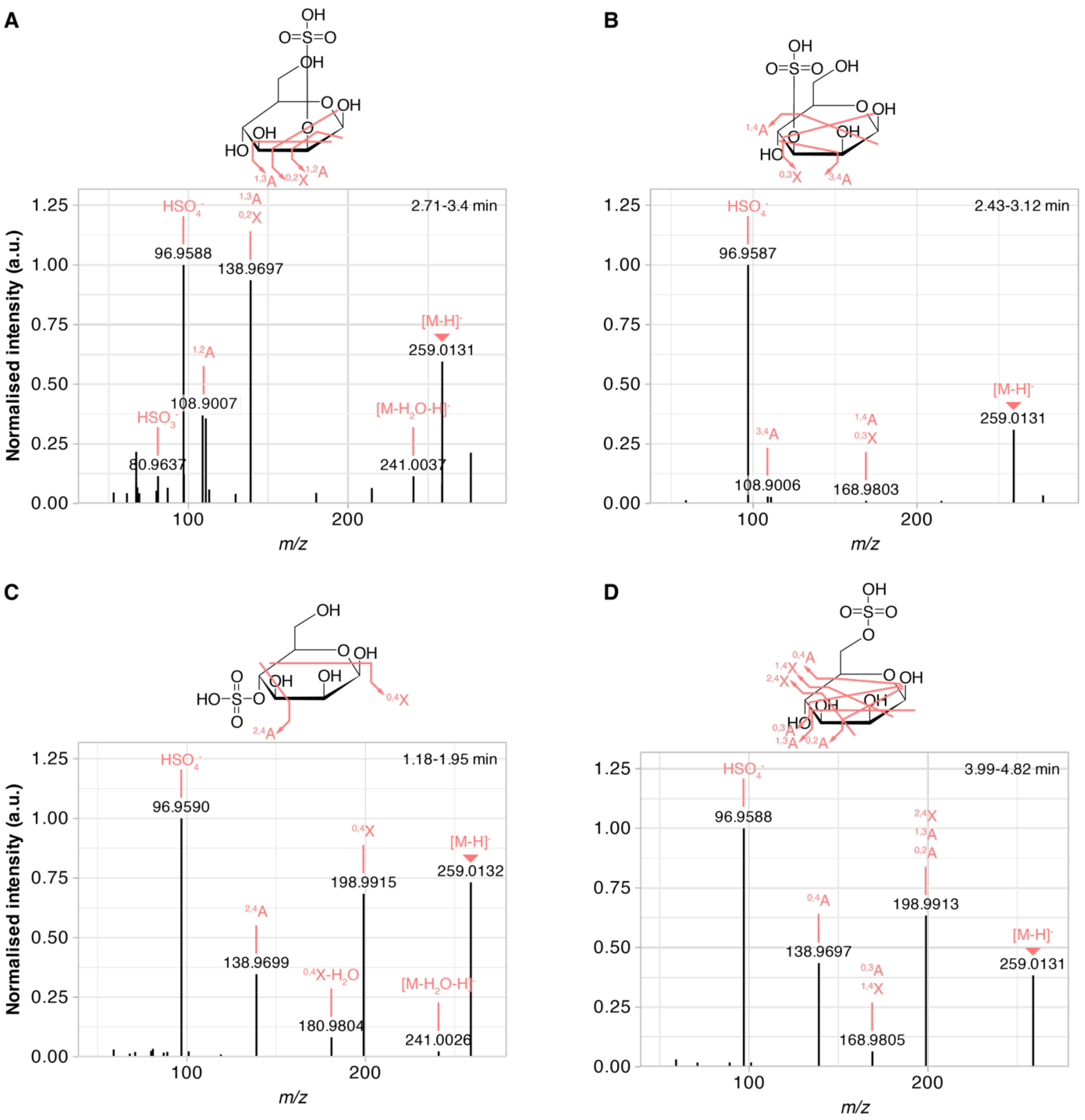

Fig. S10.

Fragmentation patterns discriminate different positions of sulfate on mannose. Averaged and normalized fragmentation spectra from LC-MS/MS experiments with mannose-2-sulfate (**A**), mannose-3-sulfate (**B**), mannose-4-sulfate (**C**) and mannose-6-sulfate (**D**). Structures show observed cleavages, with annotations according to the nomenclature of Domon & Costello (1988).

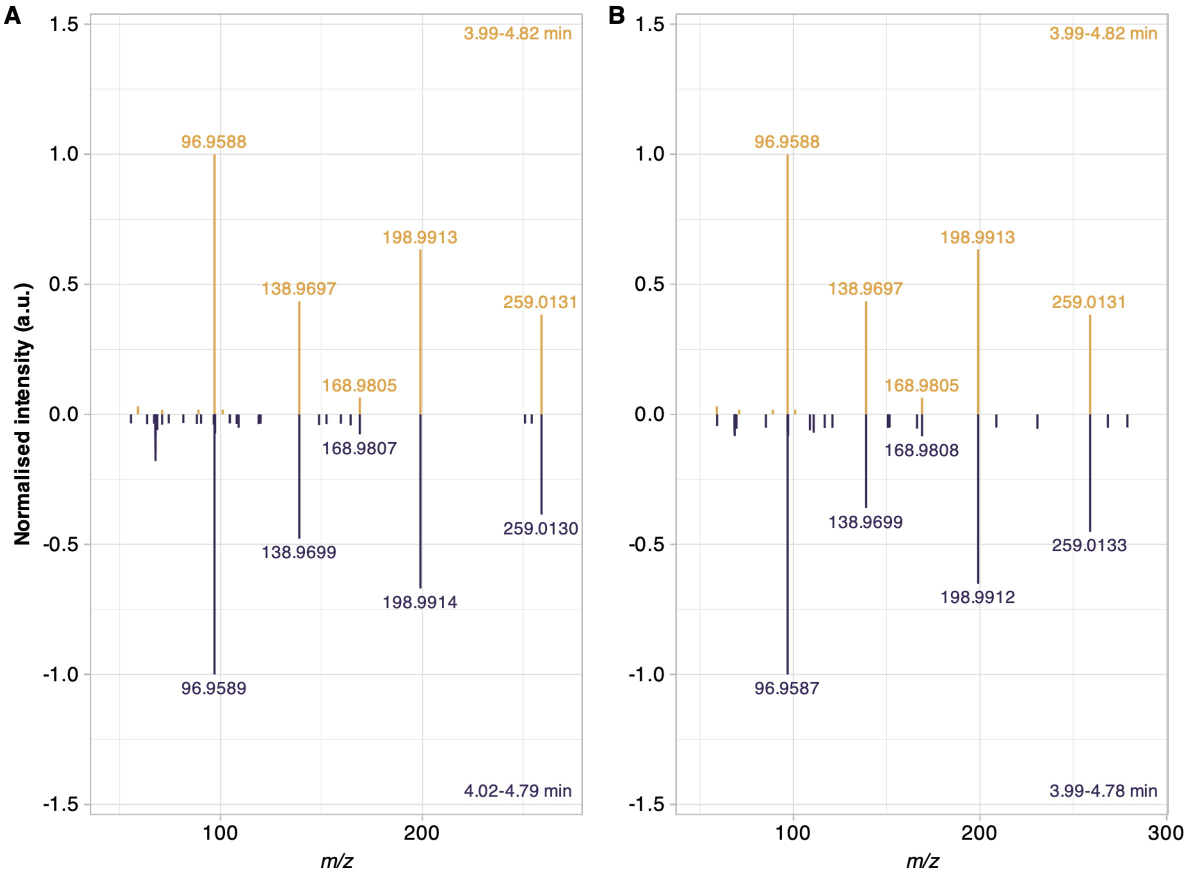

Fig. S11.
Comparison of fragmentation patterns confirms that digestion with PbSulf1-GH195 and GH92_1 yields mannose-6-sulfate. MS/MS spectra from fragmentation of mannose-6-sulfate (upper panels, yellow) and duplicate digests (lower panels, purple). Retention times of spectra used for averaging are in the corners.

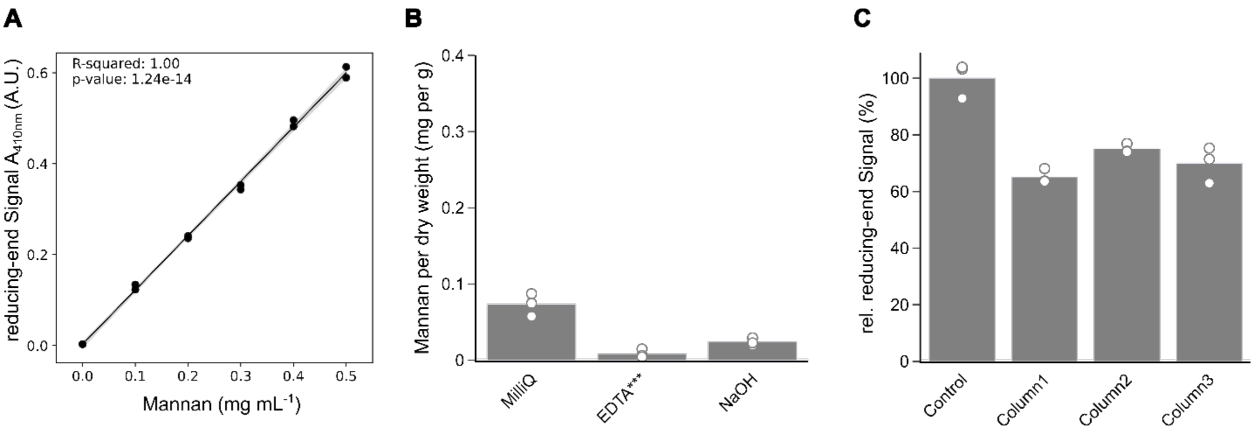

Fig. S12.

Quantification of sulfated α-mannan. (**A**) Product formation of PbSulf1-GH195 and GH92_1 follows a linear trend. Net reducing end signal is calculated as absorbance difference between digested and undigested samples. (**B**) *Conticribra weissflogii* biomass was extracted sequentially using hot water, EDTA or NaOH. Mannan content in dialyzed extracts were enzymatically quantified after AEX enrichment. ***Dry weight of dialyzed and dried EDTA extracts was below 0.1 mg. (**C**) Processing control to determine loss of mannan during the enrichment procedure with anion exchange chromatography (AEX). Defined amounts of mannan were loaded on three different AEX columns of the same type and recovery was calculated using reducing end signal after enzyme digestion (n=3).

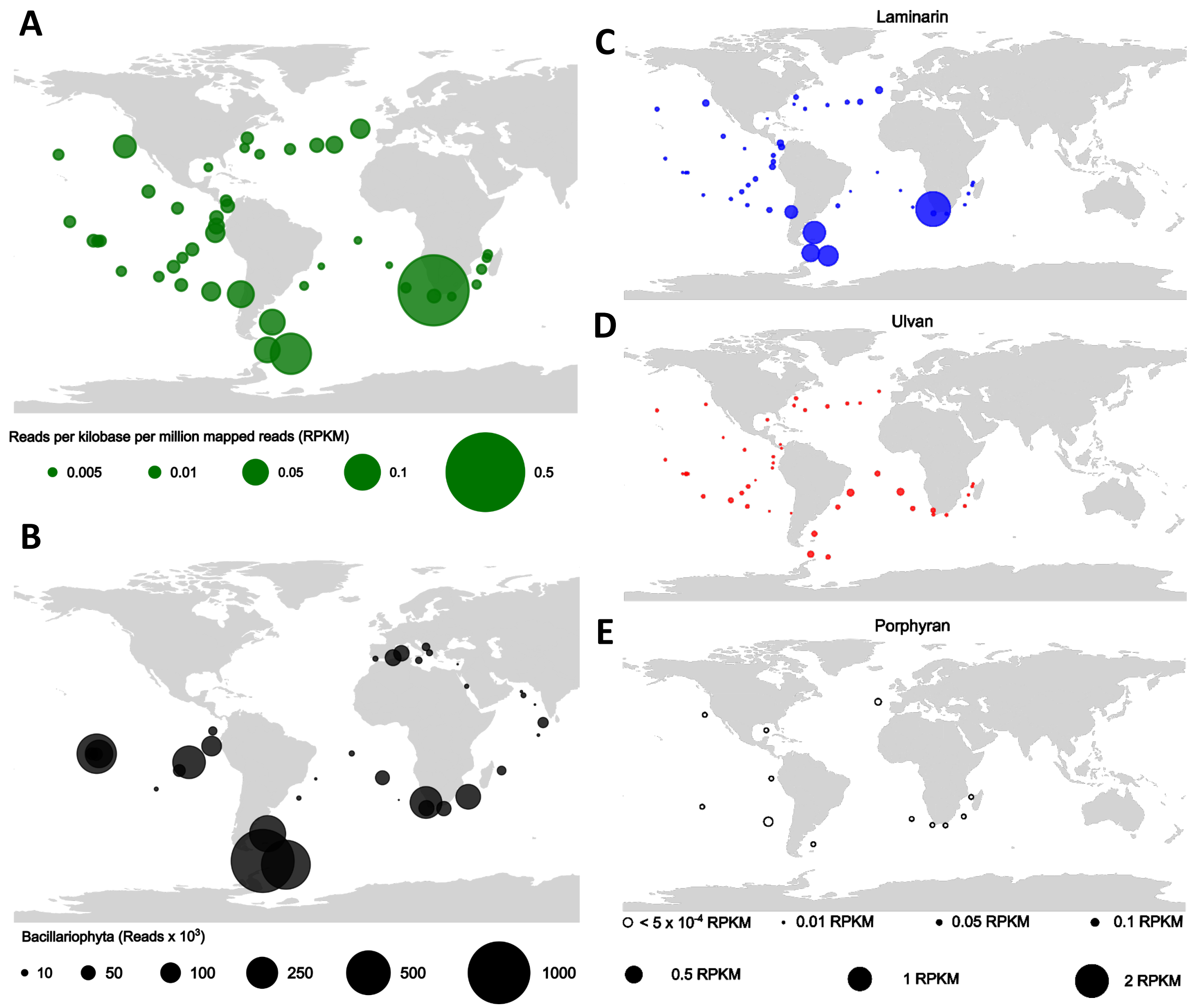

Fig. S13. Global abundance of the mannan polysaccharide utilization locus (PUL) from *Polaribacter sp*. Hel1_33_49 and selected reference PULs specific for other glycans in metagenome data from the TARA Oceans database. (**A**) The DNA sequence of the Mannan PUL was mapped against the raw metagenome reads of Tara Oceans surface water samples filtered at sizes ranging 0.2-3 µm across 45 different stations (62). Read abundances were calculated in terms of RPKM and normalized by length of the PUL and displayed as filled circles. Corresponding plots for the laminarin PUL from *Aurantivirga* MAG C_MB344 (38), the ulvan PUL from *Formosa agariphila* KMM 3901T (68) and the porphyran PUL from *Zobellia galactanivorans* DsijT (63) can be found in SI. (**B**) Raw read abundance of the clade of *Bacillariophyta* within the V9 rDNA metabarcoding dataset from Tara Oceans surface water samples filtered at sizes ranging 5-20 µm across 35 stations. Please note: No diatom data were available for the remaining stations. (**C-E**) The sequences from the respective PUL were mapped against the raw reads from *TARA* Oceans at the respective stations. Counts were normalized by the length of the PUL and

are displayed as filled circles according to resulting RPKM. (**C**) Laminarin PUL from *Aurantivirga* MAG C_MB344. (**D**) Ulvan PUL from *Formosa agariphila* KMM 3901^{T}. (**E**) Porphyran PUL from *Zobellia galactanivorans* Dsij^{T}*.* Porphyran PUL was detected at 12 out of 45 stations with low read abundances. For better visibility these are displayed at lower scale with empty circles. Values ranged from 1-5 × 10^{-4} RPKM.

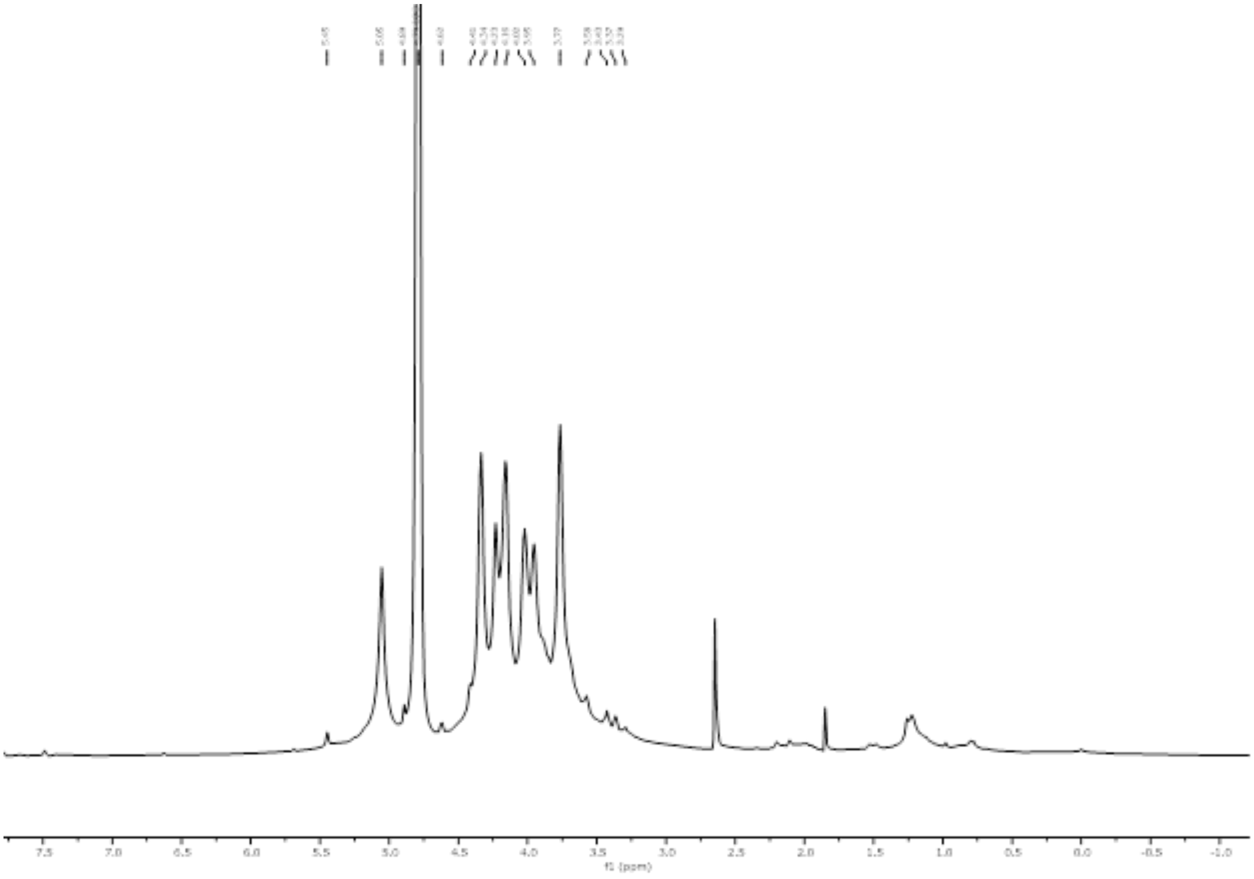

Fig. S14. ^{1}H NMR of the mannan*.*

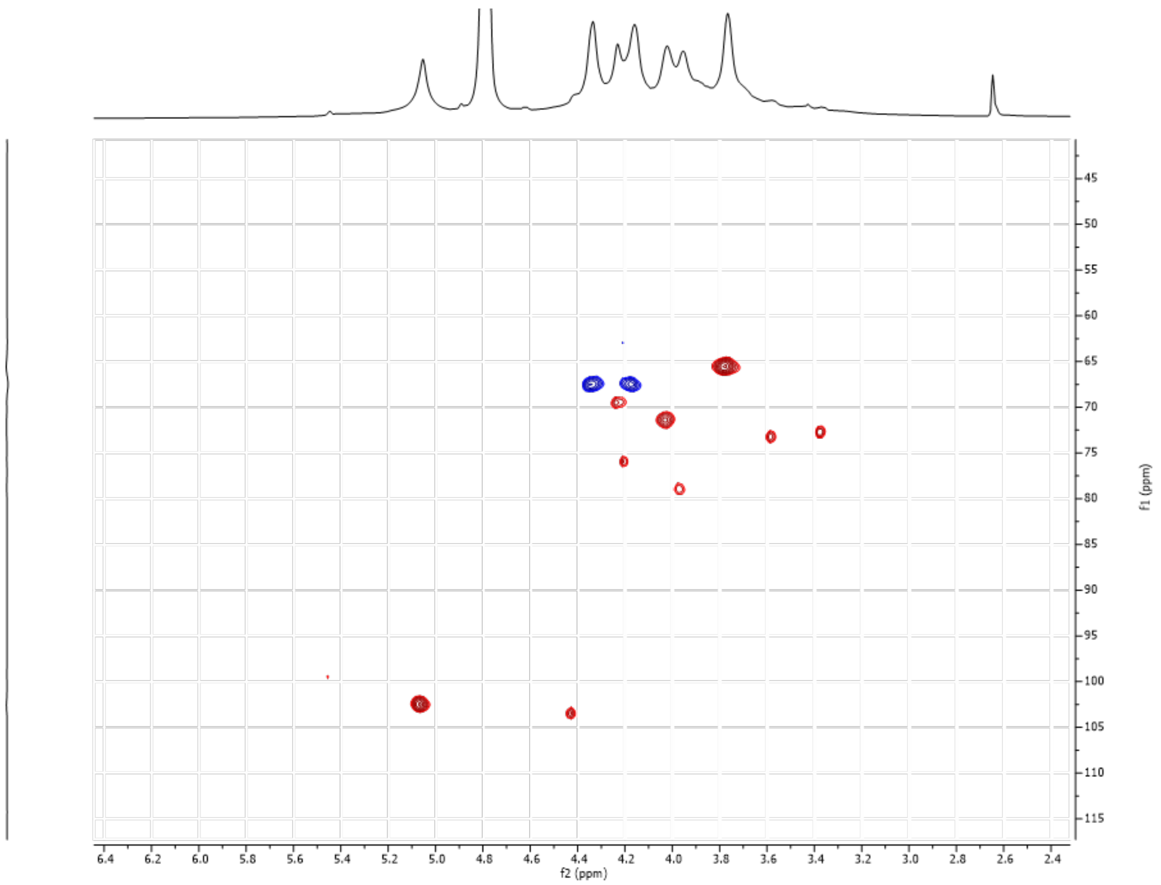

Fig. S15 ^{1}H-^{13}C HSQC NMR of the mannan from *C. weissflogii*.

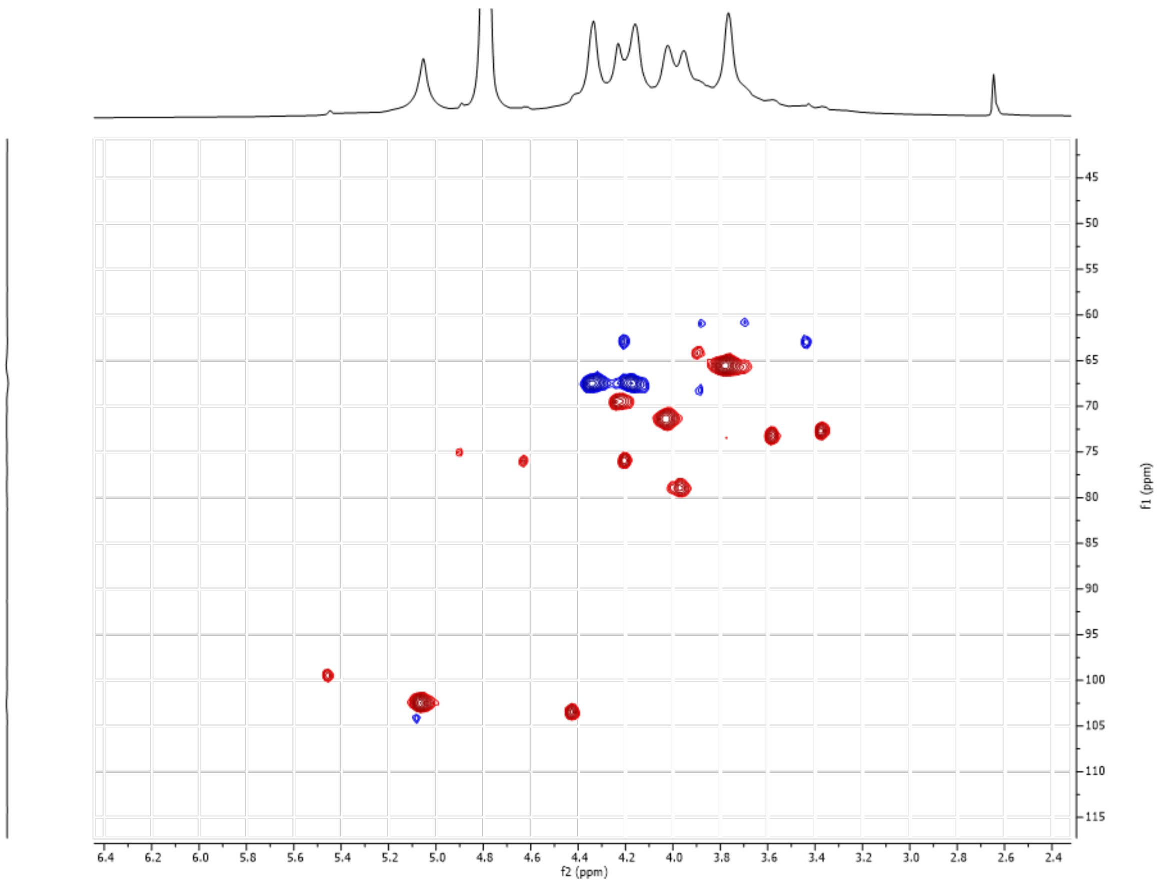

Fig. S16. ^{1}H-^{13}C HSQC NMR mannan from *C. weissflogii* displayed at higher intensity to show minor peaks.

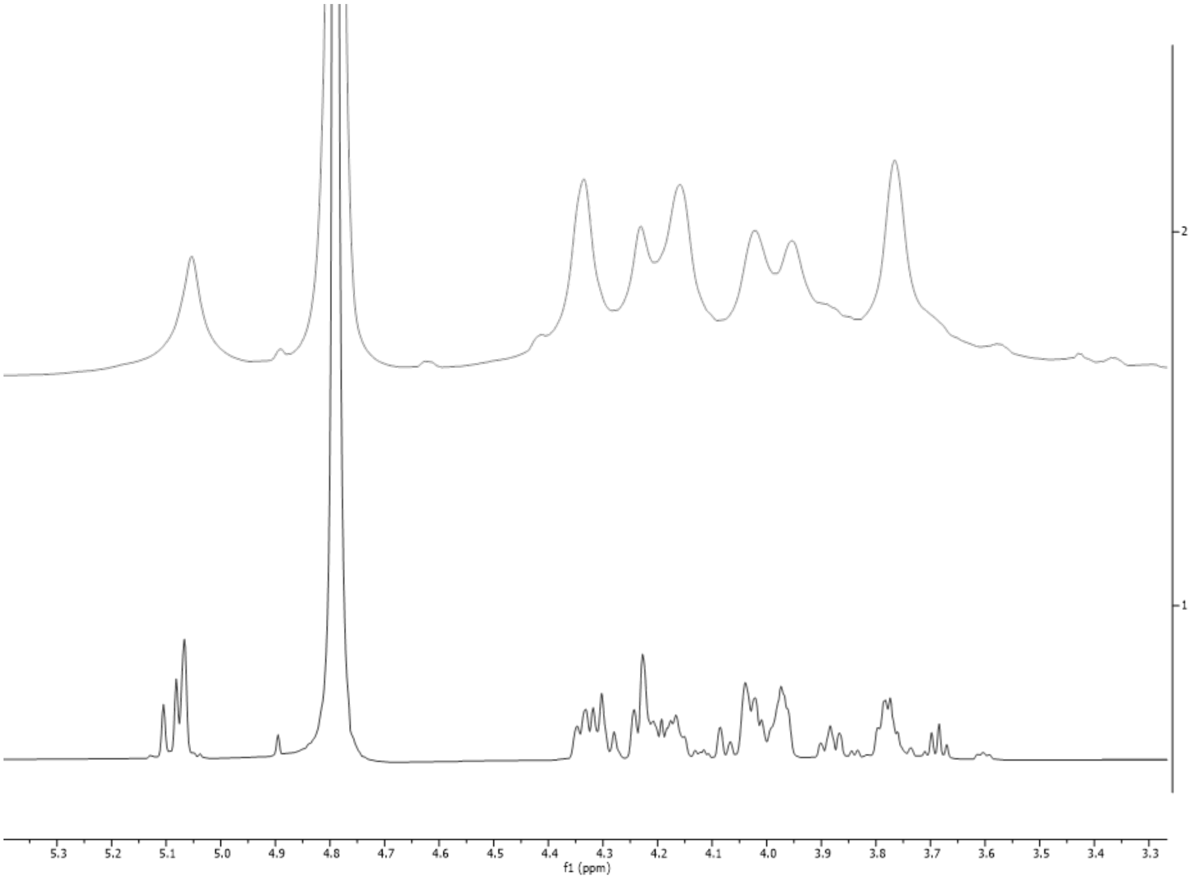

Fig. S17. Stacked ^{1}H NMR of *C. weissflogii* mannan (top) and synthetic α-1,3 6-*O*-sulfated mannan (bottom).

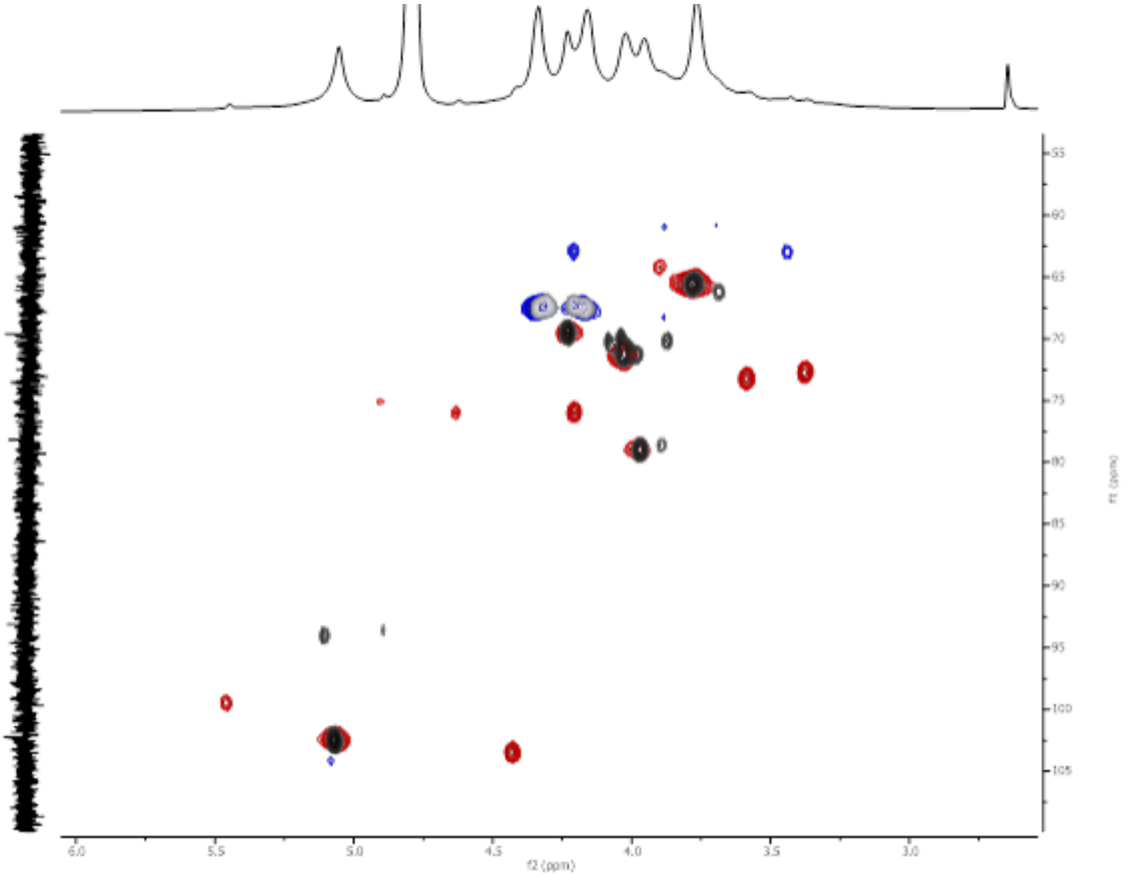

Fig. S18. Superimposed ^{1}H-^{13}C HSQC NMRs of mannan from *C. weissflogii* (red blue) and synthetic α-1,3-6-*O*-sulfated mannan (greyscale).

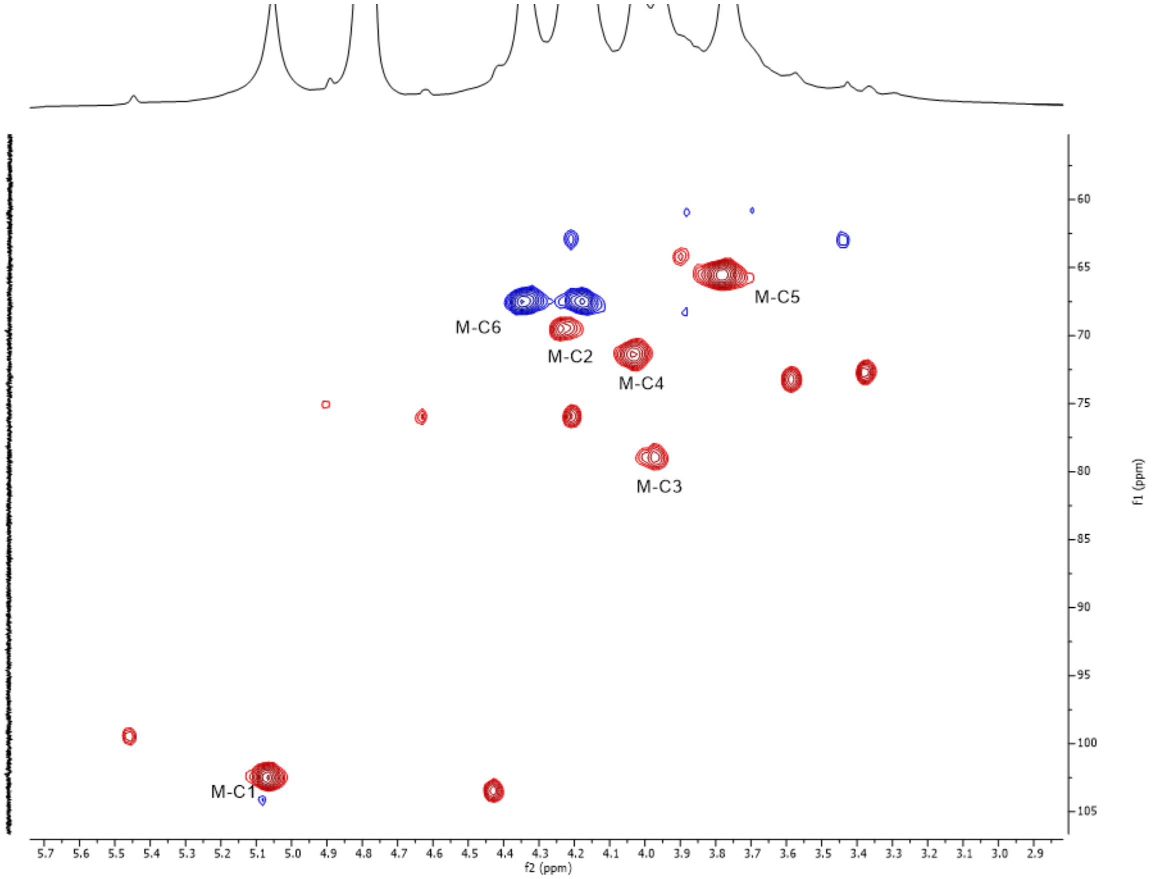

Fig. S19. ^{1}H-^{13}C HSQC NMR of mannan from *C. weissflogii* with major mannose peaks labelled.

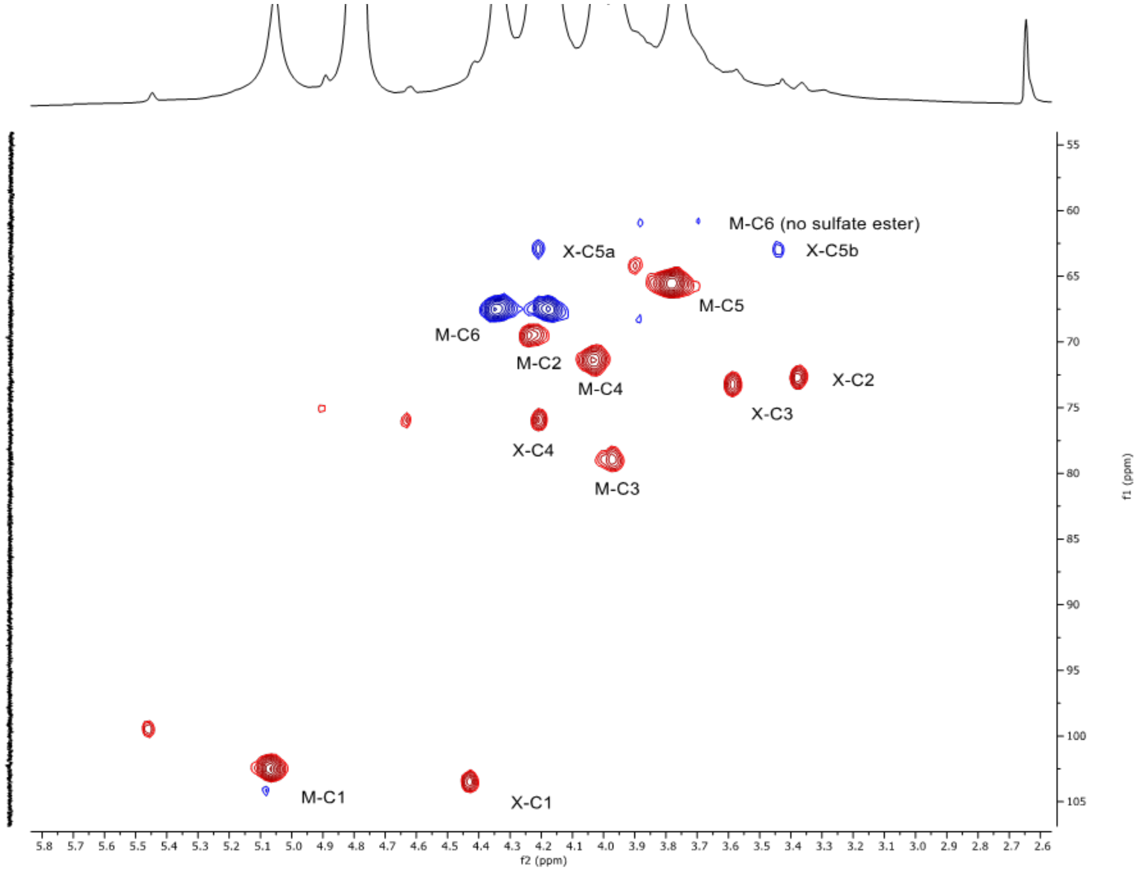

Fig. S20. ^{1}H-^{13}C HSQC NMR mannan with mannose from *C. weissflogii* (M) and possible xylose (X) branch peaks labelled.

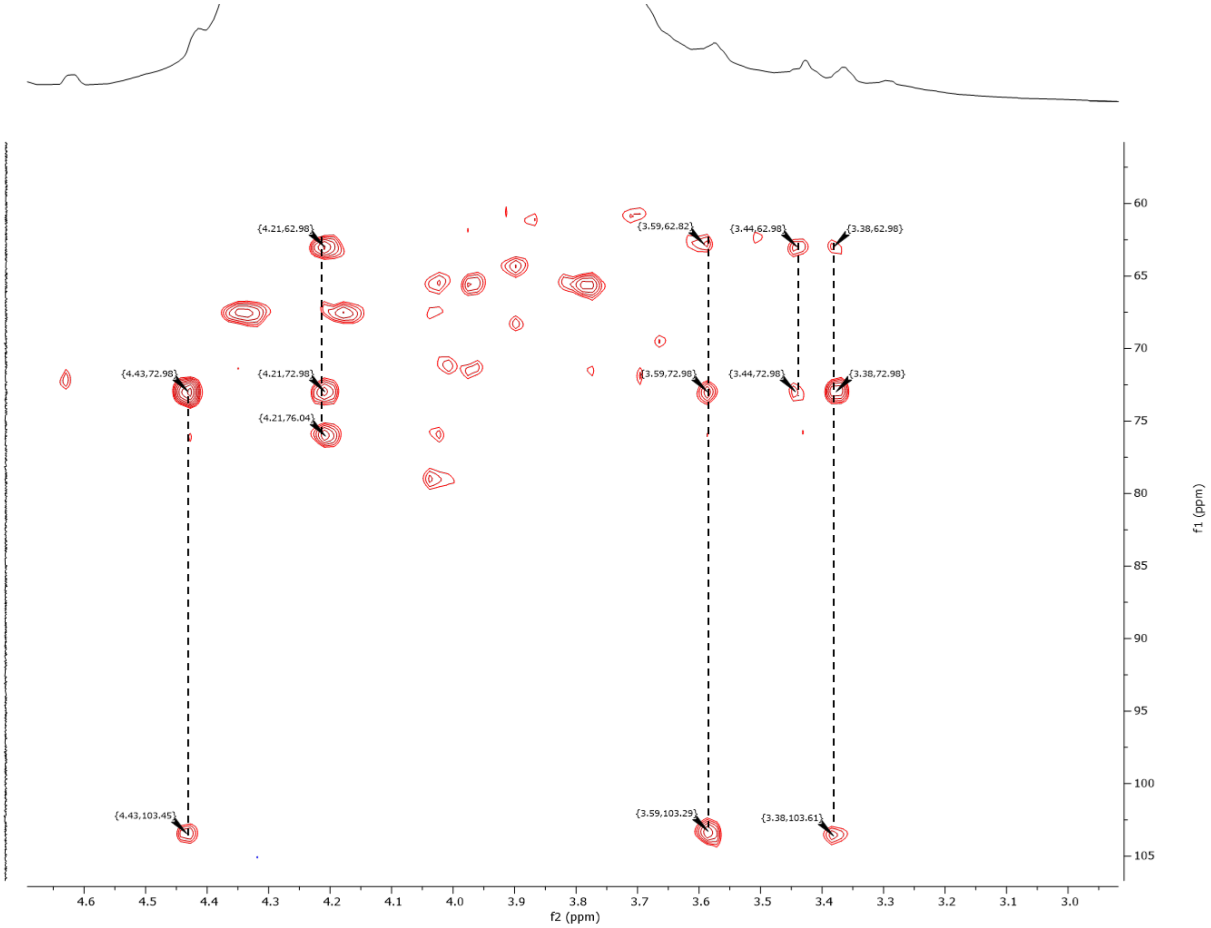

Fig. S21. ^{1}H-^{13}C HSQC TOCSY of mannan from *C. weissflogii*. Highlighted are putative xylose cross-peaks

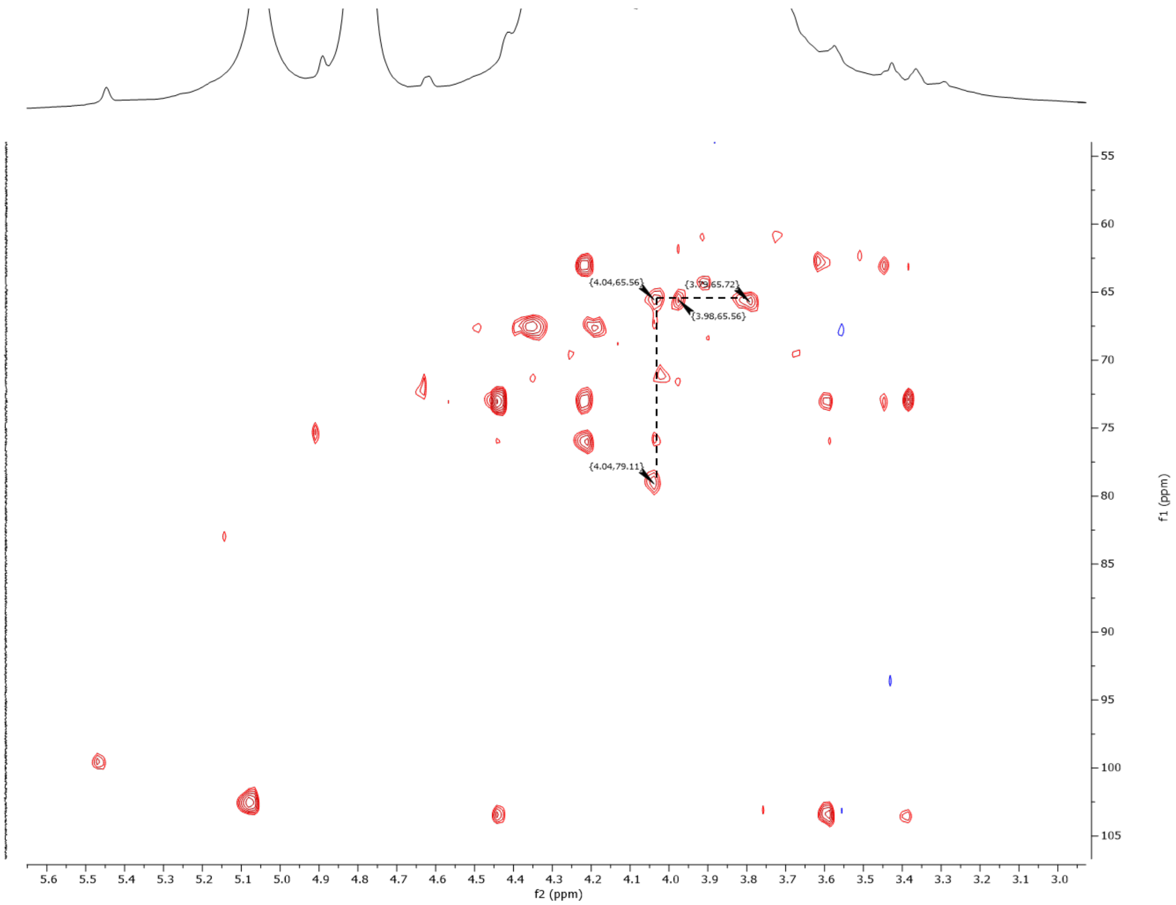

Fig. S22. ^{1}H-^{13}C HSQC TOCSY of *C. weissflogii* mannan. Highlighted are mannose cross-peaks

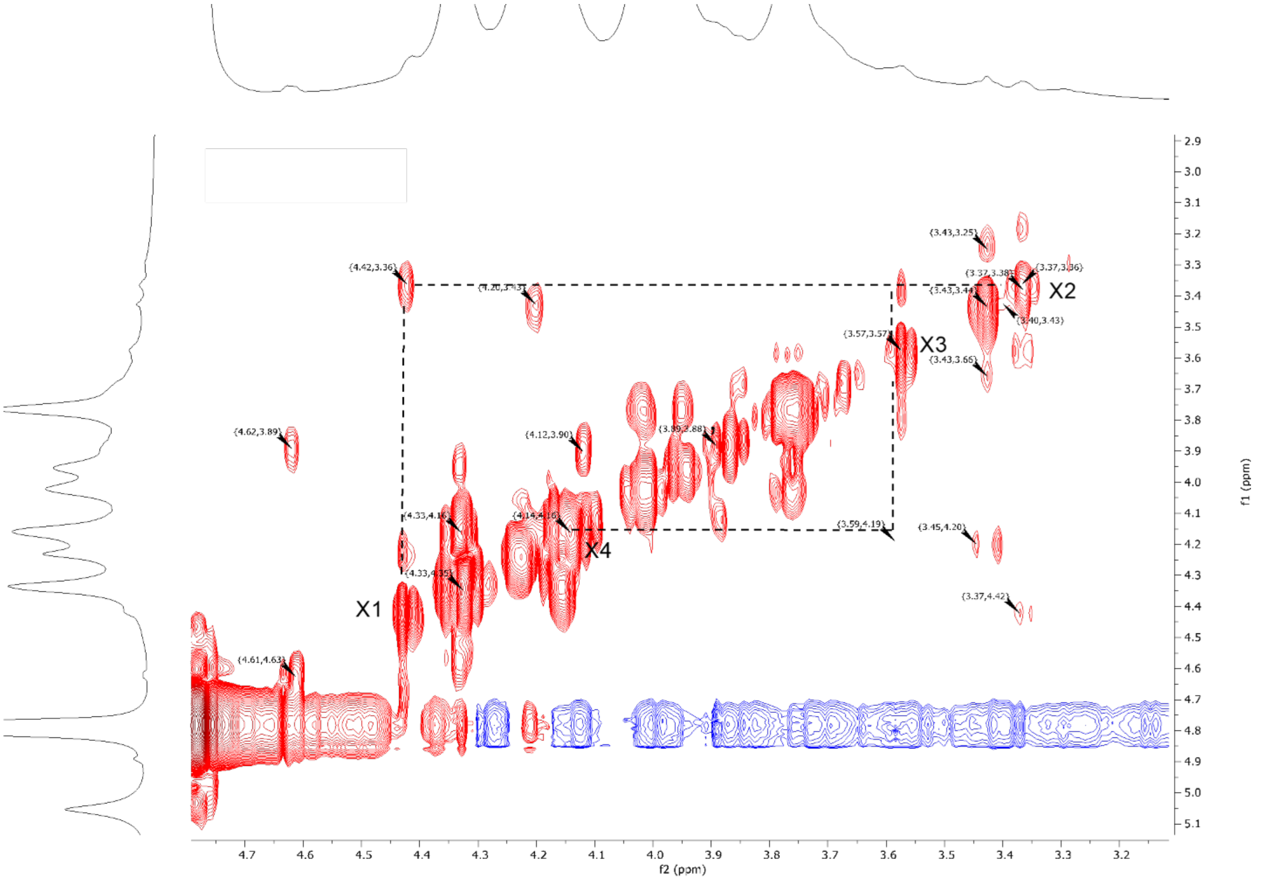

Fig. S23. ^{1}H-^{1}H COSY of *C. weissflogii* mannan. 4.42 ppm (H-1 xylose-like) vicinal coupling to 3.37 ppm (H-2 xylose-like).

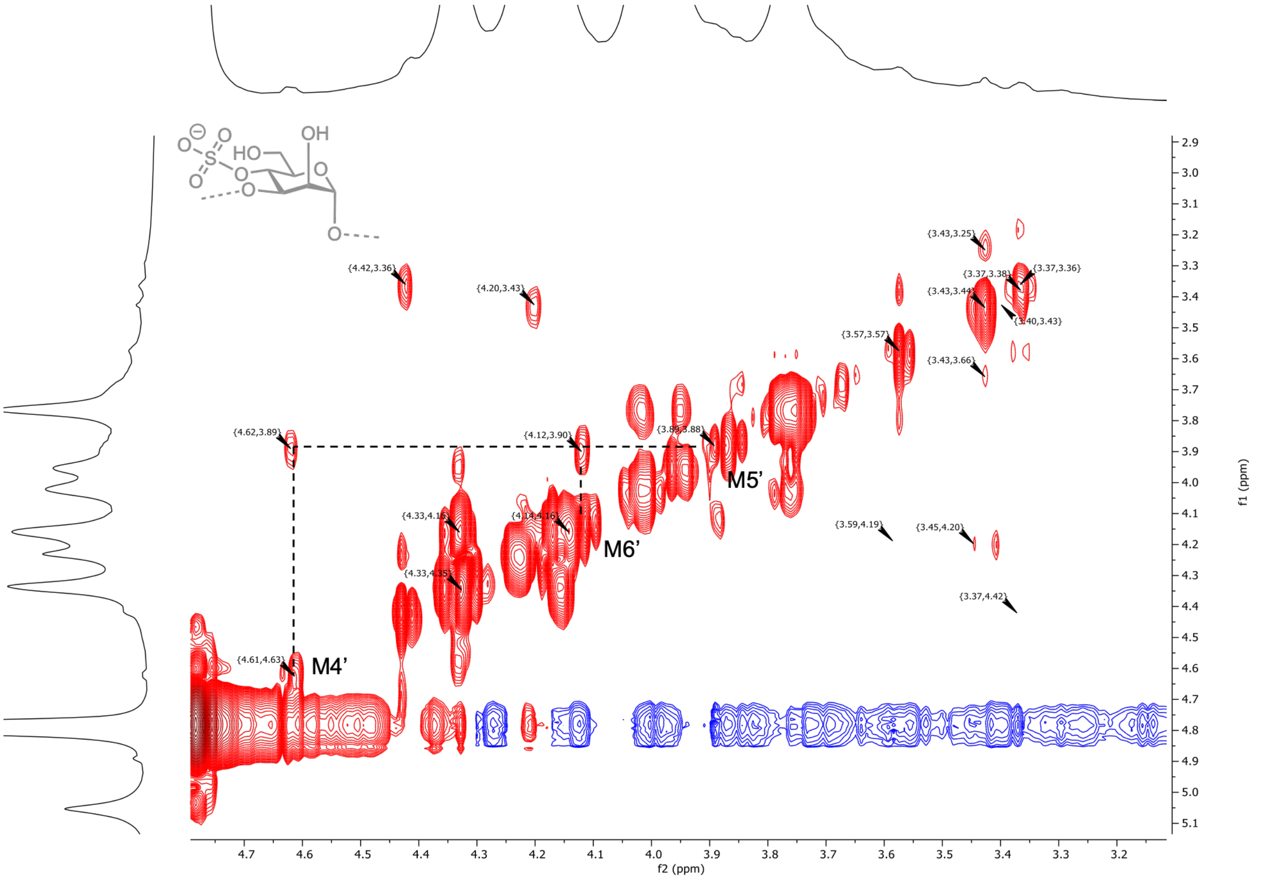

Fig. S24. ^{1}H-^{1}H COSY of mannan. Mannose (cross-peak 4-*O*-sulfation) coupled a H-5, 4.64 ppm to 3.89 ppm suggesting a minor quantity of 4-*O*-sulfation.

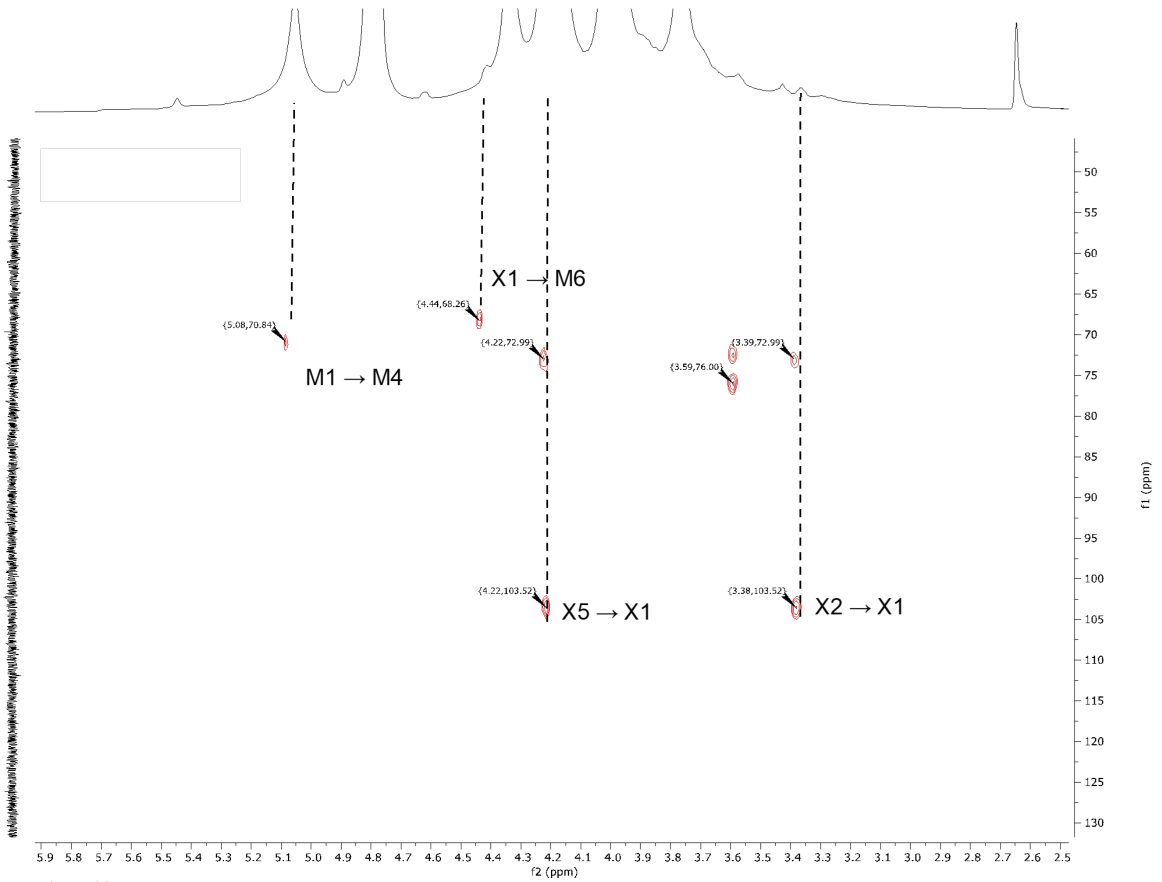

Fig. S25. ^{1}H-^{13}C HMBC of sulfated mannan.

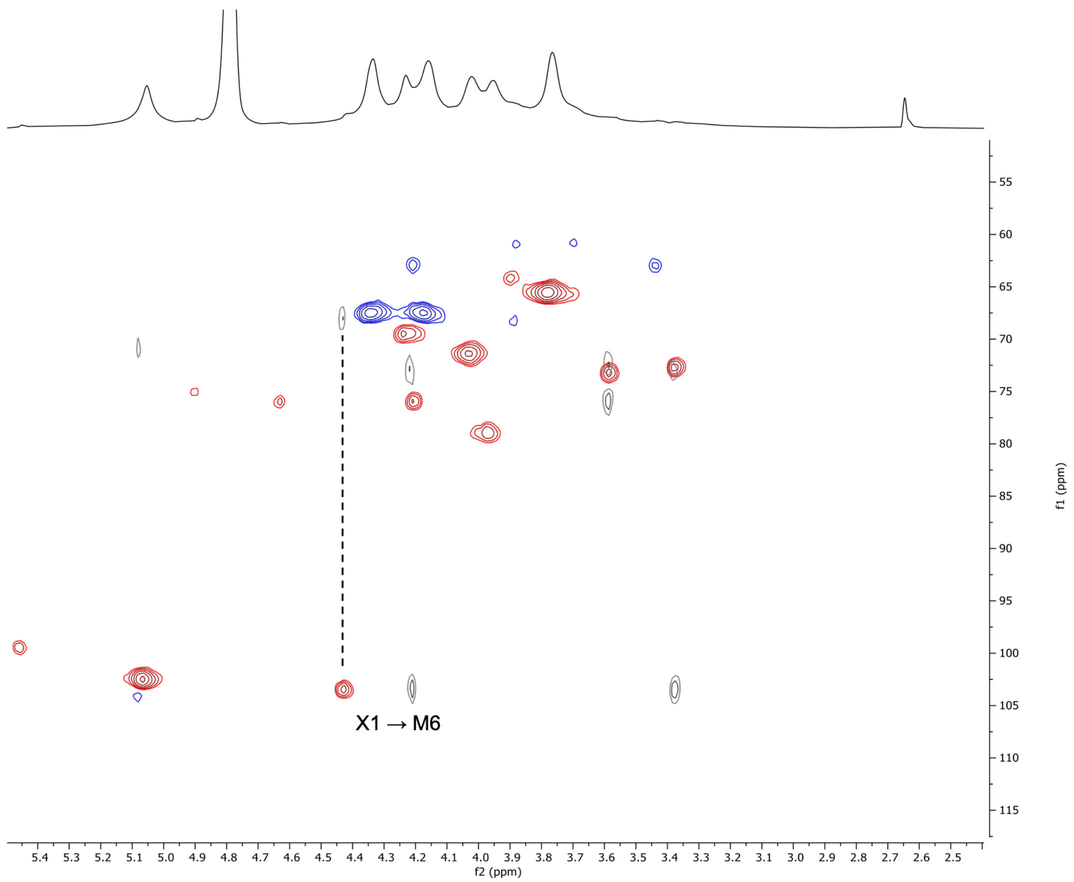

Fig. S26. Superimposed ^{1}H-^{13}C HMBC (greyscale) and ^{1}H-^{13}C HSQC (red/blue) NMR spectra.

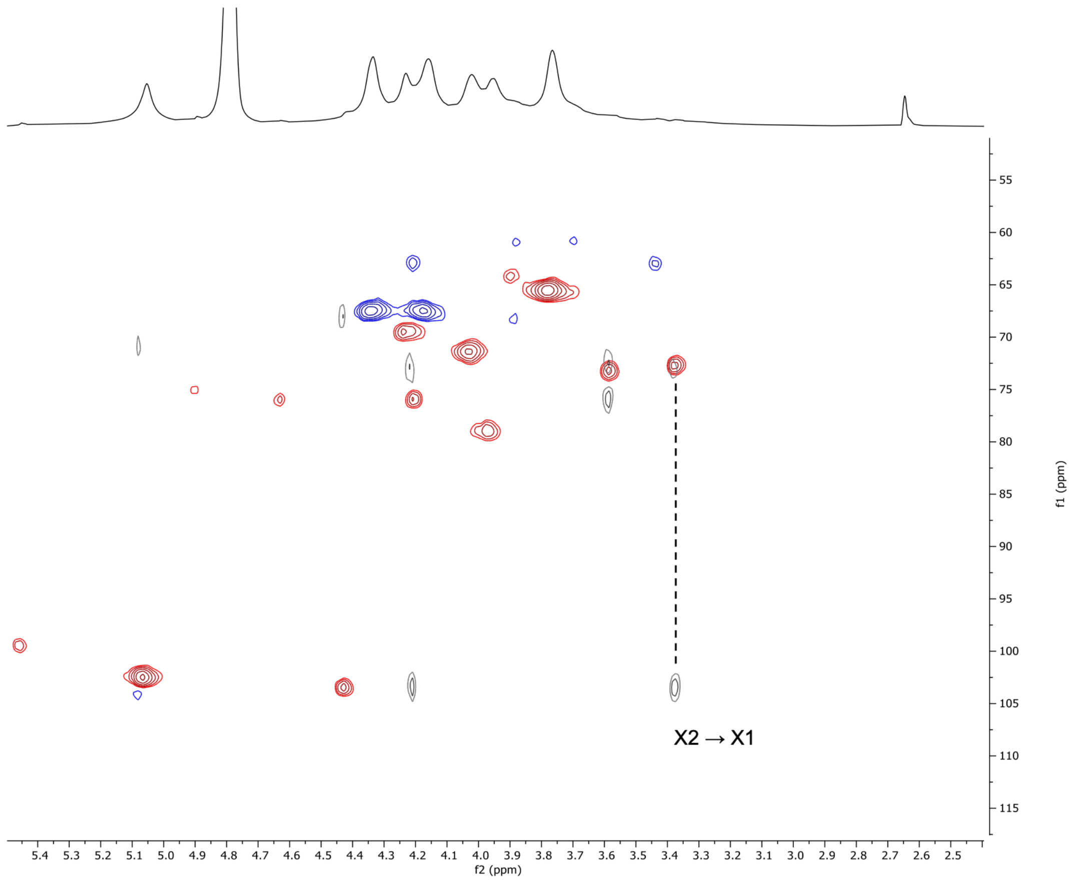

Fig. S27. Superimposed ^{1}H-^{13}C HMBC (greyscale) and ^{1}H-^{13}C HSQC (red/blue) spectra.

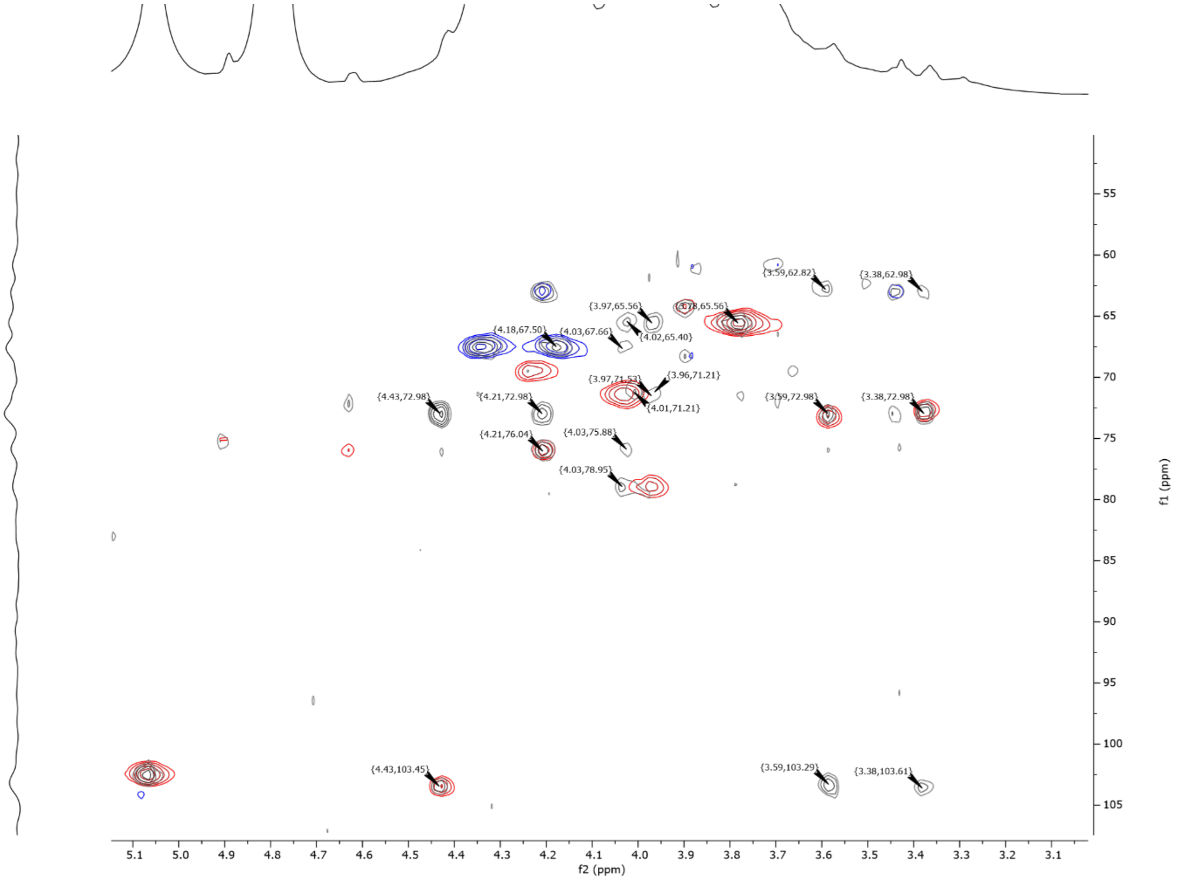

Fig. S28. Superimposed ^{1}H-^{13}C TOSCY (greyscale) and ^{1}H-^{13}C HSQC (red/blue) spectra.

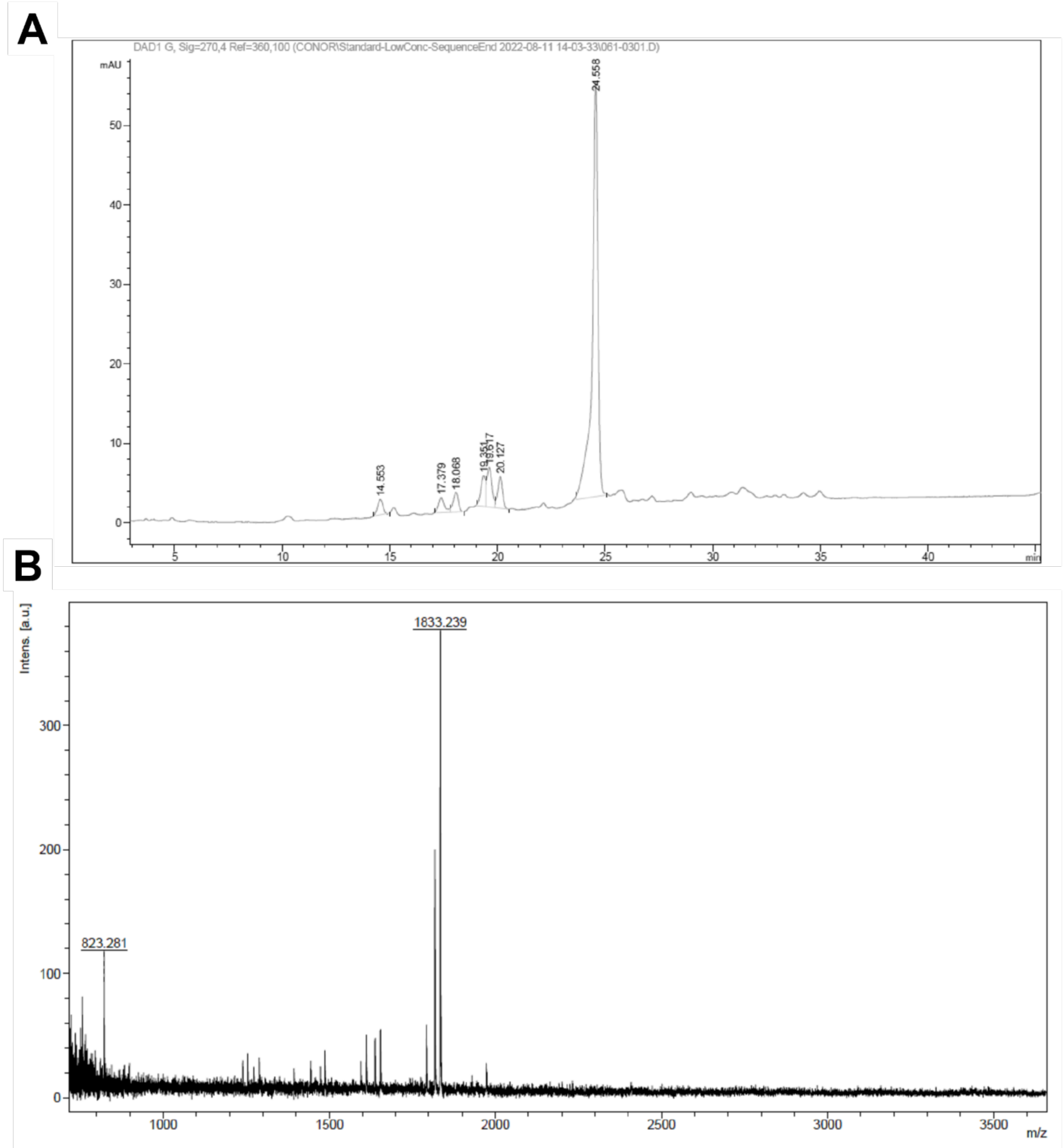

Fig. S29. Automated assembly of mannan trisaccharide. (**A**) Crude NP-HPLC of automated assembly up to trisaccharide. Trace is UV 280nm. HPLC Method 1 (20 to 55EA). (**B**) MALDI-TOF spectrum of mannotriose. Chemical Formula: $C_{107}H_{94}KO_{26}$. Expected mass, 1833.5670, observed mass 1833.239.

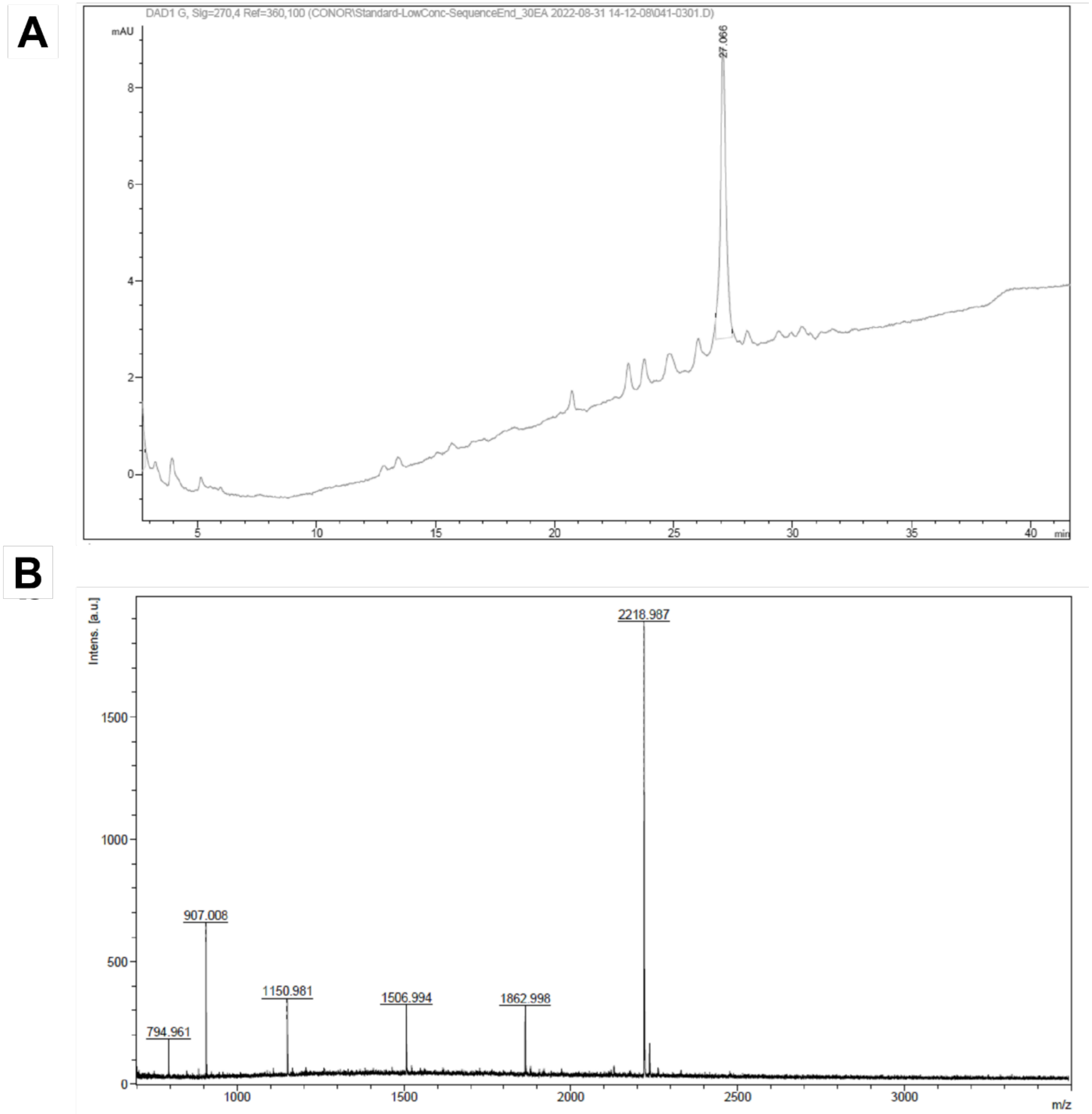

Fig. S30. a NP-HPLC of automated assembly of hexasaccharide**.** (**A**) NP-HPLC of 6-mer-t-weiss with no-fmoc. Signal is UV 270nm and HPLC method 2 (30 to 90EA). (**B**) MALDI-TOF of mannose-1,3-hexasacccaride. Note Fmoc protecting groups are removed. Chemical Formula: $C_{122}H_{124}NaO_{38}$. Expected mass, 2219,7668, Observed, 2218.987.

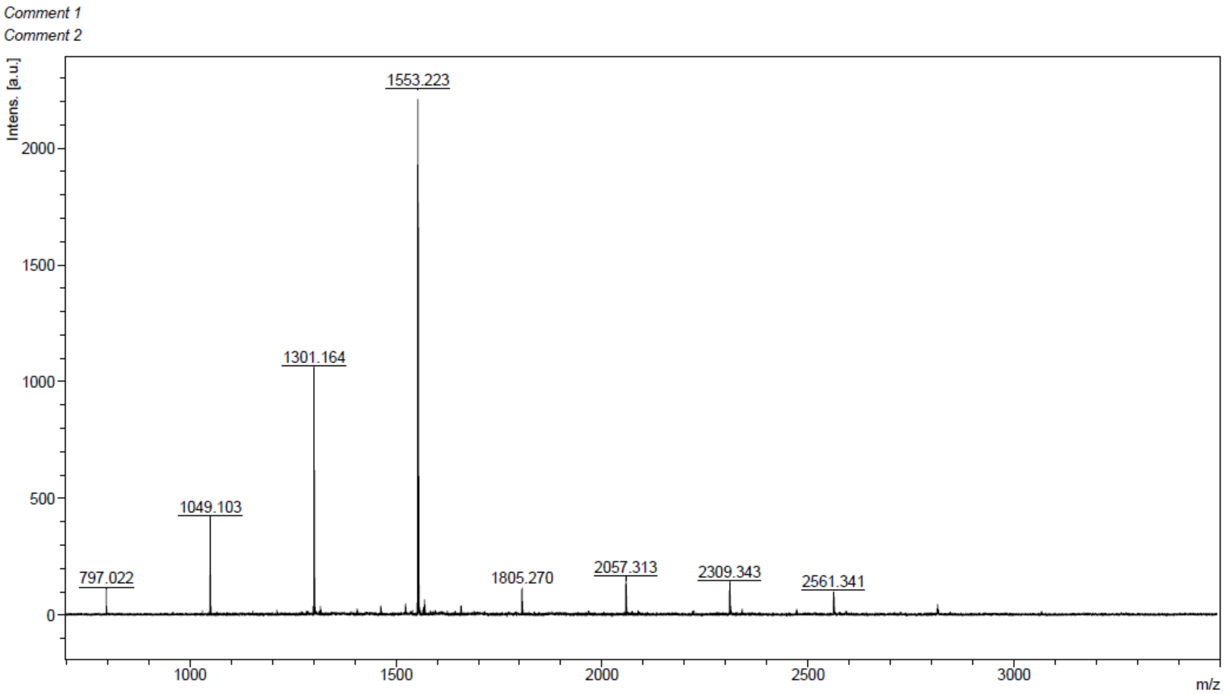

Fig. S31. MALDI-TOF of mannose-1,3-hexasacccaride with ester protecting groups removed. Chemical Formula: $C_{78}H_{98}NaO_{31}$. Expected mass, 1553,5990; Observed, 1553,223.

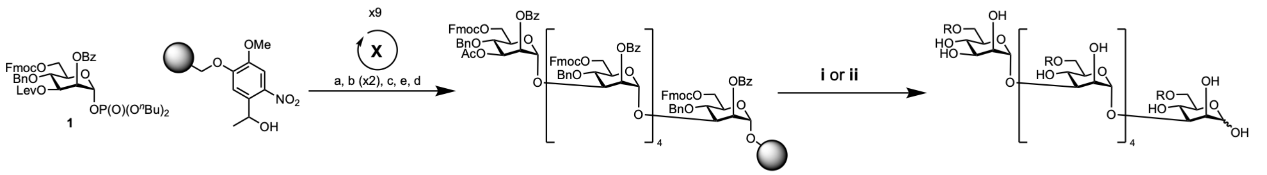

i = modules g, f, and h. **2**, 0.7 mg 2.3% R = SO_3H
ii = modules f and h. **3**, 0.9 mg 4.5 % R = H,

Scheme S1. Automated glycan assembly of sulfated mannan oligosaccharide.

Table S1.

Comparison of the *C. weissflogii* mannan to a synthetic standard. *difference is *C. weissflogii* mannan minus synthetic glycan.

**Mannan (Synthetic)**	^{1}H	^{13}C	**1H Δδ (ppm)**	**13C Δδ (ppm)**
**H-1**	5.07 (5.07)	102.49 (102.49)	-	-
**H-2**	4.24 (4.23)	69.43 (69.43)	0.01ppm	-
**H-3**	3.97 (3.97)	78.95 (78.85)	-	0.1 ppm
**H-4**	4.03 (4.03)	71.37 (71.21)	-	0.16 ppm
**H-5**	3.78 (3.78)	65.56 (65.56)	-	-
**H-6a**	4.34 (4.31)	67.50 (67.50)	0.03 ppm	-
**H-6b**	4.18 (4.17)	67.50 (67.50)	0.01 ppm	-

Table S2.

Polaribacter strains grown in HaHa 100V medium with or without mannan containing anionic exudates from *T. w.* (0.5-5M fraction after AEX).

**Growth of strain (OD_{600nm})**	Medium	+ 0.5-5 M Mannan
Polaribacter sp. Hel1_33_49	0.168	0.410
Polaribacter sp. Hel1_33_78	0.187	0.411
Polaribacter sp. Hel1_33_96	0.149	0.383
Polaribacter sp. Hel1_88	0.109	0.212

Table S3. Activity assays of enzymes on mannan containing anionic exudates from *T. w.* (0.5-5M fraction after AEX), pure mannan or on synthetic substrates. n.d.: no activity detected. -/-: not tested.

Source Organism	Locus Tag	Family	Predicted localization	Activity on 0.5-5M Mannan containing fraction	Activity on pure mannan	Activity on pnp-substrate
Polaribacter sp. Hel1_33_78	Pb1068	S1_15	SPI (75%)		n.t.	n.t.
	Pb1069	S1_N.C.	SPII (96%)		n.t.	n.t.
	Pb1070	S1_51	SPI (89%)		n.t.	n.t.
	Pb1072	S1_53	SPII (99%)		n.t.	n.t.
	Pb1095	S1_71	SPII (99%)		n.t.	n.t.
	Pb1096	S1_11	SPII (99%)		n.t.	n.t.
	Pb1097	S1_71	SPII (85%)		n.t.	n.t.
	PbSulf2 (Pb1078)	S1_11	SPII (99%)	✓	✓	n.t.
	PbSulf1-GH195 (Pb1059)	S1_51 GH195	T9SS	✓	✓	n.t.
	Pb1065	GH92	SPII (95%)		n.t.	
	Pb1066	GH92	none		n.t.	pnp-α-D-Mannose
	PbGH92_1 (Pb1075)	GH92	SPI (94%)	✓	□	n.t.
	Pb1076	GH3	SPI (96%)		n.t.	pnp-β-D-Galactose
	Pb1077	GH3	SPI (89%)		n.t.	
	Pb1081	GH92	SPI (93%)		n.t.	pnp-α-D-Mannose
	Pb1091	GH88	none		n.t.	
	Pb1093	GH92	SPII (99%)		n.t.	
	Pb1094	GH2	SPI (85%)		n.t.	n.d
	Pb1063	GH99-like	none		n.t.	n.t.
	Pb1064	GH99-like	none		n.t.	
	Pb1071	CE	SPI (88%)		n.t.	n.t.
	Pb1100	PLx	T9SS	✓		n.t.
	Pb1053	Hypothetical	SPI (91%)		n.t.	n.t.
	Pb1054	Hypothetical	SPII (81%)		n.t.	n.t.
	Pb1055	Hypothetical	SPI (97%)		n.t.	n.t.
	Pb1057	Hypothetical	SPI (83%)		n.t.	n.t.
	Pb1058	Hypothetical	T9SS		n.t.	n.t.
Ochrovirga pacifica S85	OpSulf1	S1_51	SPI (50%) SPII (50%)	✓	✓	n.t.
	OpGH195	GH195	SPI (75%) SPII (25%)	✓	✓	n.t.

Table S4. Data collection and refinement statistics for OpSulf1.

**X-ray source**	DESY P11
**Wavelength (Å)**	1.0332
**Space group**	P 43
**Unit cell**	
**a, b, c (Å)**	115.27 115.27 169.83
**α, β, γ (º)**	90 90 90
**Resolution range, (Å)**	44.07 - 1.489 (1.51 - 1.49)
**R-merge**	0.08265 (1.815)
**Completeness (%)**	99.9 (98.06)
**Multiplicity**	6.9 (6.6)
**Mean I/sigma(I)**	11.53 (0.99)
**No. of reflections**	4948642 (156063)
**No. of unique reflections**	715080 (23500)
**Mosaicity**	0.060
Refinement	
**R_{work}/R_{free}**	0.1646/0.1927
**Number of non-hydrogen atoms**	18834
**Macromolecules**	16844
**Water**	1778
**Ligands**	5
**Protein residues**	2077
**B factors**	
**Overall**	23.90
**Protein**	23.02
**Water**	31.38
**Ligands**	22.05
**R.m.s deviations**	
**Bond lengths (Å)**	0.018
**Bond angles (º)**	1.84
**Ramachandran statistics (%)**	
**Favored**	96.52
**Allowed**	3.48
**Outliers**	0.0
**Rotamer outliers (%)**	0.61
**Clashscore**	3.06
**PDB accession code**	9FVT

Statistics for the highest-resolution shell are shown in parentheses.

Table S5. Confirmation of sulfated mannose position based on retention time and MS/MS. Fragmentation ions generated from HCD fragmentation of 259.0129 *m/z* with a window of 0.4 Da. Alpha-mannan was digested with PbSUlf1-GH195 and PbGH92_1.

Sample	Retention time (min)	*m/z*	Relative abundance	Ion formula	Annotation
Synthetic mannose-2-sulfate	3.0514	80.9637	0.11	HSO_3	HSO_3
		96.9588	1.00	HSO_4	HSO_4
		108.9007	0.37	$CHSO_4$	$^{1,2}A$
		138.9697	0.94	$C_2H_3O_5S$	$^{1,3}A$, $^{0,2}X$
		241.0037	0.11	$C_6H_9O_8S$	$[M-H_2O-H]^-$
		259.0131	0.59	$C_6H_{11}O_9S$	$[M-H]^-$
Synthetic mannose-3-sulfate	2.5346	96.9587	1.00	HSO_4	HSO_4
		108.9006	0.03	$CHSO_4$	$^{3,4}A$
		168.9803	0.01	$C_3H_5O_6S$	$^{0,3}X$, $^{1,4}A$
		259.0131	0.31	$C_6H_{11}O_9S$	[M-H]-
Synthetic mannose-4-sulfate	1.3593	96.959	1.00	HSO_4	HSO_4
		138.9699	0.35	$C_2H_3O_5S$	$^{2,4}A$
		180.9804	0.08	$C_4H_5O_6S$	$^{0,4}X-H_2O$
		198.9915	0.68	$C_4H_7O_7S$	$^{0,4}X$
		241.0026	0.02	$C_6H_9O_8S$	$[M-H_2O-H]^-$
		259.0132	0.73	$C_6H_{11}O_9S$	[M-H]-
Synthetic mannose-6-sulfate	4.138	96.9588	1.00	HSO_4	HSO_4
		138.9697	0.43	$C_2H_3O_5S$	$^{0,4}A$
		168.9805	0.06	$C_3H_5O_6S$	$^{0,3}A$, $^{1,4}X$
		198.9913	0.63	$C_4H_7O_7S$	$^{0,2}X$, $^{1,3}A$, $^{2,4}X$
		259.0131	0.38	$C_6H_{11}O_9S$	[M-H]-
Digest replicate 1	4.2803	96.9587	1.00	HSO_4	HSO_4
		138.9699	0.36	$C_2H_3O_5S$	$^{0,4}A$
		168.9808	0.08	$C_3H_5O_6S$	$^{0,3}A$, $^{1,4}X$
		198.9912	0.65	$C_4H_7O_7S$	$^{0,2}X$, $^{1,3}A$, $^{2,4}X$
		259.0133	0.45	$C_6H_{11}O_9S$	[M-H]-
Digest replicate 1	4.2105	96.9589	1.00	HSO_4	HSO_4
		138.9699	0.48	$C_2H_3O_5S$	$^{0,4}A$
		168.9807	0.08	$C_3H_5O_6S$	$^{0,3}A$, $^{1,4}X$
		198.9914	0.67	$C_4H_7O_7S$	$^{0,2}X$, $^{1,3}A$, $^{2,4}X$
		259.013	0.39	$C_6H_{11}O_9S$	[M-H]-

Table S6. Primers used for cloning of PUL enzymes. Genes marked with an asterisk were cloned using Gibson-assembly into a regular pet28A vector.

Locus Tag	Primer Forward (5‘ → 3‘)	Primer Reverse (5‘ → 3‘)
**pet28A_P1**	AGCGTTAAUGTCTGGCTTCTGATAAAG	ATTCCTCTCCCUATAGTGAGTCGTATTAATTTC
**pet28A_P2**	AGGGAGAGGAAUTGTGAGCGGATAAC	ATTAACGCUTCTGGAGAAACTCAACG
**pet28A**	ATCCGAAUTCGAGCTCCGTCG	ACCGCTGCUGTGATGATGATGATGATGG
**Pb1053**	AGCAGCGGUTGTTTAATCTCCTCTCTTG	ATTCGGAUTTACTTATTTTTATTTTCTAAG
**Pb1054**	AGCAGCGGUTCTAAACACGCTAAAACTAG	ATTCGGAUTTATTTTCGATTATTTACTTTTC
**Pb1055**	AGCAGCGGUCAACAAACAGTTTATGTTTC	ATTCGGAUTTAACTAATGTTTTCTGACAC
**Pb1057**	AGCAGCGGUCAAACTACCGTTTATGTTTC	ATTCGGAUTTAAACAGAAAGTTTAGTTGC
**Pb1058**	AGCAGCGGUATGAAAACAATCCAATTAATATC	ATTCGGAUTTACATACTAATGTTTTCTAAGAC
**Pb1059**	AGCAGCGGUCAAACCCAAAAACC	ATTCGGAUTTAGCTAAATTTCTCATTTG
**Pb1060**	AGCAGCGGUCAAGAAACTATAAAAGTAGAGGG	ATTCGGAUCAGTAAAGCAACTTAGATTGC
**Pb1062**	AGCAGCGGUTGCACAAACAAATTTGAAG	ATTCGGAUCTGAAGTTACAGATACAATAGGG
**Pb1063**	AGCAGCGGUATGATATCTTTTTCATGTAC	ATTCGGAUGAGATTGATTTGATTTTC
**Pb1064**	AGCAGCGGUATGCTAACGCTTTTC	ATTCGGAUGGATTTTTATTTCGTTG
**Pb1065**	AGCAGCGGUTGTAGTAATTCAGAAAACAG	ATTCGGAUTTTCATAGGTAATCTGTTG
**Pb1066**	AGCAGCGGUAAAATCACAAAAACAAC	ATTCGGAUTGTTACTCTTTAATTATTTAGG
**Pb1068***	CTGGTGCCGCGCGGCAGCCATATGGCTAGCAAAA AACCAAATGTCATTGTAT	ATCTCAGTGGTGGTGGTGGTGGTGCTCGAGTTAAT TATTTTTCCAAATTTC
**Pb1069***	CTGGTGCCGCGCGGCAGCCATATGGCTAGC AAGTCTAAAAACGAACTTGTA	ATCTCAGTGGTGGTGGTGGTGGTGCTCGAGTTA TTTTTTTGTTTTATTTTG
**Pb1070***	CTGGTGCCGCGCGGCAGCCATATGGCTAGC CAAAACACTAAAAAGCCAAAC	ATCTCAGTGGTGGTGGTGGTGGTGCTCGAGTTACT TTACAAACTCTGCGTAAG
**Pb1071**	AGCAGCGGUCAAAAAACAATAGATACAATAG	ATTCGGAUGAATGATTTTTAATAGATGC
**Pb1072***	CTGGTGCCGCGCGGCAGCCATATGGCTAGCACAC AAGACACTGCTAAAAAG	ATCTCAGTGGTGGTGGTGGTGGTGCTCGAGTTAAA TTAATTTTCGTTTTGA
**Pb1075**	AGCAGCGGUCAAGAAACTGATGACAACAC	ATTCGGAUTTATTTTGGTATTACTGC
**Pb1076**	AGCAGCGGUCAAGAAAAATTACCGTATC	ATTCGGAUTGTTAACATACGCATCTC
**Pb1077**	AGCAGCGGUCAAGAAAAATTACCGTATC	ATTCGGAUGGCTTTTGAGAAATGG
**Pb1078***	CTGGTGCCGCGCGGCAGCCATATGGCTAGCAAAA CTGAAAAAAAAGTGGAA	ATCTCAGTGGTGGTGGTGGTGGTGCTCGAGTTAAT TCGCTGGCTCAACTCT
**Pb1081**	AGCAGCGGUCAAAATAAATTAACCGATAAAG	ATTCGGAUTTAATTTACTTTACCGTTTGC
**Pb1082**	AGCAGCGGUCAAGGAATTATTGAAAATTCG	ATTCGGAUGCTTCAACCATTAGAAAAATAG
**Pb1086**	AGCAGCGGUCAAAACTCATTTACCACAAG	ATTCGGAUTTATTCTACAGCGTGCAC
**Pb1091**	AGCAGCGGUATGATACTAAGTGATATAAATATTC	ATTCGGAUTTAATGAATCATATCAAAG
**Pb1093**	AGCAGCGGUTGTAATACTCAAGGACAAAC	ATTCGGAUCTATTTGTATTTTTTTAGACTC
**Pb1094**	AGCAGCGGUCAAGATAGGTTACTCTCTAAAG	ATTCGGAUCCATTTTATAAATGCAAG
**Pb1095**	AGCAGCGGUTGCTTTTCATCGAAGTC	ATTCGGAUCGGGTTTTGGTTTAGC

**Pb1096**	AGCAGCGGUTGTTCTAGTGCTAAACC	ATTCGGAUTTAAGATTTACTATCCCAATAC
**Pb1097***	CTGGTGCCGCGCGGCAGCCATATGGCTAGCCAAA AAACACAAGTCACTCAA	ATCTCAGTGGTGGTGGTGGTGGTGCTCGAGTTAAT TTAATCTAGTATTTAA
**Pb1098**	AGCAGCGGUCAAACAAAAATAATTGCGC	ATTCGGAUCGCCTTATTTAGTTGCGT
**OpSulf1**	AGCAGCGGUCAAAATAATAAAAAACCAAATATTG	ATTCGGAUCTATAGTTAATAGGAAAAAAATTG
**OpGH195**	AGCAGCGGUCAAGACCTATTAAATCATGTAC	ATTCGGAUTTATTTTAATGGAAGTTTTTG

Chemical Synthesis

Automated glycan assembly

Solvents were taken from an anhydrous solvent system (JC Meyer-solvent systems) to prepare activator, acid wash (TMSOTf), and capping solutions. The building blocks were co-evaporated once with toluene and dried under a high vacuum before use. All solutions were freshly prepared and kept under argon during the automation run. Final yields were calculated based on the resin loading. Resin loading was determined by performing a double glycosylation followed by DBU-promoted Fmoc-cleavage and determination of dibenzofulvene formation by measuring its UV absorbance.

Preparation of reagent solutions

Building block solution: Building block (0.09 mmol) was dissolved in DCM (1 mL).

Fmoc deprotection solution: Either a solution of 20% piperidine in DMF (v/v) (module E1) or a solution of 20% triethylamine in DMF (v/v) was prepared (module E2).

Acid wash solution/phosphate donor activator: TMSOTf (0.45 mL, 2.49 mmol) was dissolved in DCM (40 mL).

Capping solution: A 50 mL solution of 10% acetic anhydride and 2% methanesulfonic acid in DCM (v/v) was prepared.

Lev deprotection solution: A solution of hydrazine acetate (725 mg) in the mixture of pyridine (40 mL), acetic acid (10 mL) and water (2.5 mL) was prepared.

Modules for Automated Solid-Phase Synthesis

Resin preparation for synthesis

The traceless linker resin (50 mg, resin loading 0.4 mmol/g) was placed in the reaction vessel and swollen in DCM for 20 min at room temperature prior to synthesis. During this time, all reagent lines needed for the synthesis were washed and primed. Before the first glycosylation, the resin was washed with the DMF, THF, and DCM (three times each with 2 mL for 25 s).

TMSOTf acidic wash solution (Module a): The resin was swollen in DCM (2 mL) and the temperature of the reaction vessel was adjusted to -30 °C. Upon reaching the low temperature, TMSOTf solution (1 mL, 0.06 mmol) was added dropwise to the reaction vessel. After bubbling for 3 min, the acidic solution was drained and the resin was washed with DCM (2 mL) for 25 s.

Action	Cycles	Solution	Amount	T (°C)	Incubation time
Cooling	-	-	-	-30	-
Deliver	1	DCM	2 mL	-30	-
Deliver	1	TMSOTf solution	1 mL	-30	3 min
Wash	1	DCM	1 mL	-30	25 s

Phosphate glycosylation (Module b): The building block solution (0.1 mmol of BB in 1 mL of DCM per glycosylation) was delivered to the reaction vessel. After the set temperature was reached, the reaction was started by dropwise addition of the activator solution (1.0 mL, excess). After completion of the reaction, the solution is drained and the resin was washed with DCM, DCM/dioxane (1:2, v/v, 2 mL for 20 s), and DCM (twice, each with 2 mL for 25 s). The temperature of the reaction vessel is increased to 25°C for the next module.

Action	Cycles	Solution	Amount	T (°C)	Incubation time
Cooling	-	-	-	-30	-
Deliver	1	BB solution	1 mL	-30	-
Deliver	1	activator solution	1 mL	-30	-
Reaction time	1			-30<br>to -10	10 min<br>30 min
Wash	1	DCM	2 mL	0	25 sec
Wash	1	DCM:Dioxane	2 mL	0	20 sec
Heating	-	-	-	25	-
Wash	1	DCM	2 mL	>0	25 sec

Capping (Module c): The resin was washed twice with DMF (2 mL, 25 s) and the temperature of the reaction vessel was adjusted to 25 °C. Pyridine solution (2 mL, 10% in DMF) was delivered into the reaction vessel. After 1 min, the reaction solution was drained and the resin was washed with DCM (three times with 3 mL for 25 s). Capping solution (4 mL) was delivered into the reaction vessel. After 20 min, the reaction solution was drained and the resin was washed with DCM (three times with 3 mL for 25 s).

Action	Cycles	Solution	Amount	T (°C)	Incubation time
Heating	-	-	-	25	-
Wash	2	DMF	2 mL	25	25 s
Deliver	1	10% Py./DMF	2 mL	25	1 min
Wash	3	DCM	2 mL	25	25 s
Deliver	1	Capping solution	4 mL	25	20 min
Wash	3	DCM	2 mL	-20	25 s

Fmoc deprotection with NEt_3 (Module d): The resin was washed with DMF (three times with 2 mL for 25 s) and the temperature of the reaction vessel was adjusted to 25 °C. Fmoc deprotection solution was delivered to the reaction vessel and kept under Ar bubbling (three times with 2 mL). After 5 min, the reaction solution was drained and the resin was washed with DMF (three times with 2 mL for 25 s) and DCM (five times each with 2 mL for 25 s). The temperature of the reaction vessel was decreased to -20 °C for the next module.

Action	Cycles	Solution	Amount	T (°C)	Incubation time
Wash	3	DMF	2 mL	25	25 s
Deliver	3	Fmoc depr.	2 mL	25	5 min
Wash	3	DMF	2 mL	25	25 s
Wash	5	DCM	2 mL	25	25 s
Cooling	1	-	-	-20	-

Levulinoyl ester deprotection (Module e): The resin was washed with DCM (three times with 2 mL for 25 s) and the temperature of the reaction vessel was adjusted to 25 °C. 2 mL of Levulinoyl ester deprotection solution was delivered to the reaction vessel and kept under Ar bubbling. After 30 min, the reaction solution was drained and the resin was washed with DMF, THF and DCM (six times each with 2 mL for 25 s).

Action	Cycles	Solution	Amount	T (°C)	Incubation time
Heating	-	-	-	25	-
Wash	3	DCM	2 mL	25	25 s
Deliver	1	Lev solution	1 mL	25	30 min
Wash	6	DCM	2 mL	25	25 s
Deliver	1	Lev solution	1 mL	25	30 min
Wash	9	DCM	2 mL	25	25 s
Deliver	1	Lev solution	1 mL	25	30 min
Wash	3	DCM	2 mL	-20	25 s
Wash	6	DMF	2mL	<25	25 s
Wash	6	THF	2mL	<25	25 s
Wash	6	DCM	2mL	<25	25 s

Solid-phase synthesis

Methanolysis (Module f): The resin was suspended in anhydrous THF (4.8 mL). Then, 0.2 mL of a solution of NaOMe in MeOH (0.5 M) was added and the resin was shaken at room temperature for 16 h. The resin was then washed successively with THF, DCM, methanol, and DCM.

Sulfation (Module g): The resin was suspended in a silanized glass microwave vial in 1 mL DMF. Then, a solution of Py·SO_3 (20 eq. per OH) in DMF:Py (4 mL, 80:20 v/v) was added. The microwave vial was sealed and the temperature was raised to 50 °C with gentle stirring for 16 h. Thereafter, the reaction was cooled, and the resin was washed successively with DMF, CH_2Cl_2, methanol, and CH_2Cl_2.

Photocleavage from the solid support (Module h): Glycans were cleaved from the solid support using a batch-flow photoreactor. The resin-bound glycan (~40 mg) was suspended in DMF (4 mL) under the irradiation of an LED lamp (370nm), with stirring for 24 hours. The solution was separated from the resin using a fritted syringe and concentrated under vacuum.

Solution-phase synthesis

Hydrogenolysis (Module i1), oligosaccharides: The crude compound was dissolved in 4 mL of THF: *t*BuOH: H_2O (60:10:30). 5% Pd-C (200 mg) was added and the reaction was stirred under H_2 atmosphere for 12 h. The reaction was filtered through a pad of celite and washed with *t*BuOH and H_2O. The filtrates were concentrated *in vacuum*, and dissolved in 3.0 mL of water for RP-HPLC purification.

Hydrogenolysis (Module i2), sulfated oligosaccharides: The crude compound was dissolved in 4 mL of THF: *t*BuOH: H_2O (50:20:30). 5% Pd-C (200 mg) was added and the reaction was stirred under H_2 atmosphere for 12 h. The reaction was filtered through a pad of celite and washed with *t*BuOH and H_2O. The filtrates were concentrated *in vacuum*, and dissolved in 3.0 mL of water for RP-HPLC purification.

HPLC analysis and purification

Analytical traces of crude and pure compounds were collected using an analytic RP-HPLC Agilent 1200 Series (**Methods 1, 3, 5, 6, 7**). Purification of the crudes was conducted using a preparative RP-HPLC Agilent 1200 Series (**Methods 2, 4**).

Method 1, analytic NP-HPLC: (YMC-Diol-300 column, 150 x 4.6 mm) flow rate of 1.0 mL / min with Hex – 20% EtOAc as eluent [isocratic 20% EtOAc (5 min), linear gradient to 55% EtOAc (35 min), linear gradient to 100% EtOAc (5 min)].

Method 2 analytic NP-HPLC: (YMC-Diol-300 column, 150 x 4.6 mm) flow rate of 1.0 mL / min with Hex – 30% EtOAc as eluent [isocratic 30% EtOAc (5 min), linear gradient to 90% EtOAc (35 min), linear gradient to 100% EtOAc (5 min)].

Method 3, analytic RP-HPLC (non-sulfated oligosaccharide): (Hypercarb column, 150 x 4.6 mm, 3 µm) flow rate of 0.7 mL/min with ACN/H_2O (0.1% formic acid) as eluents [isocratic 100 % H_2O (0.1% formic acid) (5 min), linear gradient to 100% ACN (30 min)].

Method 4, prep RP-HPLC (non-sulfated oligosaccharide): (Hypercarb column, 150 x 10 mm, 5 µm), flow rate of 3.5 mL /min with H_2O (0.1% formic acid) as eluents [isocratic 100 % H_2O (0.1% formic acid) (5 min), linear gradient to 100% ACN (30 min)].

Method 5 analytic RP-HPLC (sulfated oligosaccharide): (Hypercarb column, 150 x 4.6 mm, 3 µm) flow rate of 0.7 mL/min with ACN/H_2O (0.1mM $(NH_4)_2CO_3$) as eluents [isocratic 100 % H_2O (0.1mM $(NH_4)_2CO_3$) (5 min), linear gradient to 100% ACN (30 min)].

Method 6 prep RP-HPLC (sulfated oligosaccharide): (Hypercarb column, 150 x 10 mm, 5 µm), flow rate of 3.5 mL /min with ACN/H_2O (0.1mM $(NH_4)_2CO_3$) as eluents [isocratic 100 % H_2O (0.1mM $(NH_4)_2CO_3$) (5 min), linear gradient to 100% ACN (30 min)].

Following purification, all products were lyophilized on a Christ Alpha 2-4 LD plus freeze dryer before characterization.

Compound characterisation

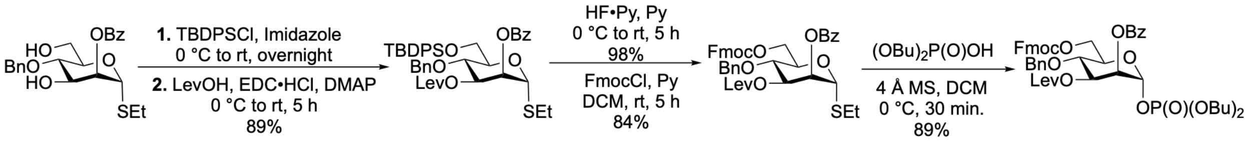

SI Scheme 1. Ethyl 2-*O*-benzoyl-4-*O*-benzyl-1-thio-α-D-mannopyranoside was prepared following literature procedures.[6]

Ethyl 2-*O*-benzoyl-4-*O*-benzyl-6-*tert*-butyl diphenylsilyl-3-*O*-levulinoyl-1-thio-α-D-mannopyranoside (4)

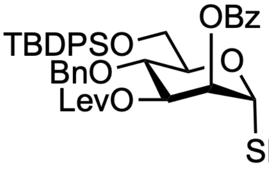

Ethyl 2-*O*-benzoyl-4-*O*-benzyl-1-thio-α-D-mannopyranoside (3.42 g, 8.18 mmol) was dissolved in anhydrous DMF (25 mL). Subsequently, imidazole (1.67 mL, 24.55 mmol, 3 eq.) and TBDPSCl (6.37 mL, 24.55 mmol, 3 eq.) were added at room temperature. The reaction was monitored by TLC and once completed was quenched by the addition of 10 % aqueous citric acid solution. The aqueous layers were extracted with CH_2Cl_2, and then the combined organic layers were dried over Na_2SO_4, filtered, and concentrated *in vacuo*. The crude material was passed through silica plug, concentrated and dried under vacuum. The intermediate was dissolved in CH_2Cl_2 (30 mL) and 4-dimethylaminopyridine (199 mg, 1.64 mmol, 0.2 eq.), levulinic acid (1.67 mL, 16.36 mmol, 2 eq.) and EDC•HCl (3.14 g, 16.36 mmol, 2 eq.) were added. Once the reaction reached completion (16 h), the organic layer was extracted with saturated aqueous $NaHCO_3$ and brine. The organic layer was dried over Na_2SO_4, filtered and concentrated. TLC. The material was purified by flash chromatography (SiO_2, Hexane/EtOAc) to give compound **4** (5.49 g, 89% over two steps) as a white solid.

^{1}H NMR (400 MHz, $CDCl_3$) δ 8.20 – 8.12 (m, 2H), 7.78 (ddt, *J* = 17.3, 6.7, 1.5 Hz, 4H), 7.69 – 7.58 (m, 1H), 7.50 – 7.43 (m, 4H), 7.38 (ddd, *J* = 8.2, 6.6, 2.9 Hz, 4H), 7.34 – 7.24 (m, 5H), 5.69 – 5.63 (m, 1H), 5.50 – 5.42 (m, 2H), 4.82 – 4.68 (m, 2H), 4.34 (t, *J* = 9.7 Hz, 1H), 4.23 (ddd, *J* = 9.7, 3.5, 1.7 Hz, 1H), 4.12 (dd, *J* = 11.4, 3.4 Hz, 1H), 3.96 (dd, *J* = 11.4, 1.7 Hz, 1H), 2.84 – 2.40 (m, 6H), 2.13 (s, 3H), 1.31 (t, *J* = 7.4 Hz, 3H), 1.16 (s, 9H). **^{13}C NMR** (101 MHz, $CDCl_3$) δ 206.3, 171.8, 165.6, 138.1, 136.0, 135.6, 135.6, 133.5, 133.4, 133.0, 130.0, 129.8, 129.7, 129.7, 128.6, 128.5, 128.4, 127.9, 127.8, 127.8, 127.7, 127.6, 81.9, 77.4, 77.3, 77.1, 76.8, 75.1, 73.1, 73.0, 72.8, 72.3, 62.6, 37.9, 29.8, 28.0, 26.9, 25.2, 19.4, 14.8. **HRMS** QTOF-MS: calcd. $C_{43}H_{51}O_8SSi$ for $[M+H]^+$ 755.3074, found 755.3098.

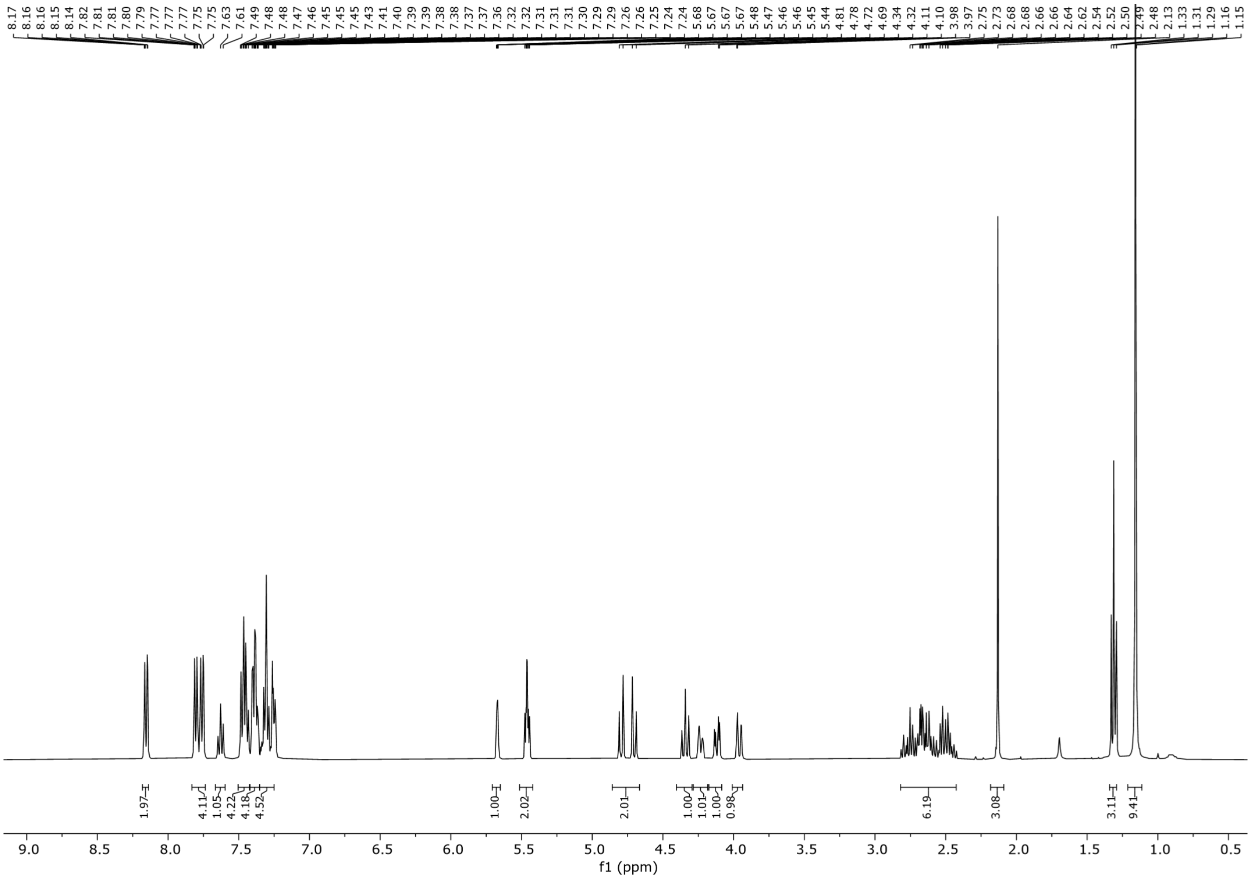

^{1}H NMR

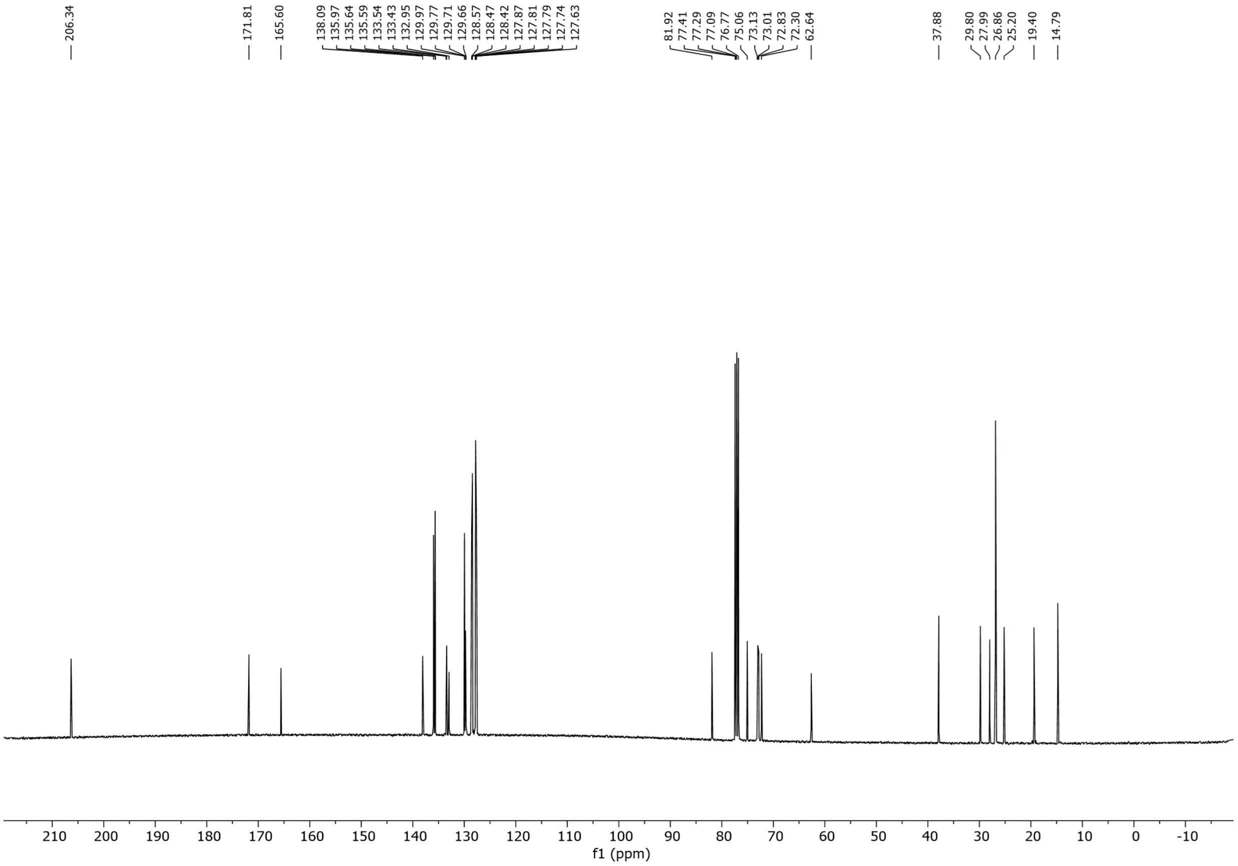
206.34
171.81
165.60
138.09
135.97
135.64
135.59
133.54
133.43
132.95
129.97
129.77
129.71
129.66
128.57
128.47
128.42
127.87
127.81
127.79
127.74
127.63
81.92
77.41
77.29
77.09
76.77
75.06
73.13
73.01
72.83
72.30
62.64
37.88
29.80
27.99
26.86
25.20
19.40
14.79
210 200 190 180 170 160 150 140 130 120 110 100 90 80 70 60 50 40 30 20 10 0 -10
f1 (ppm)

^{13}C NMR

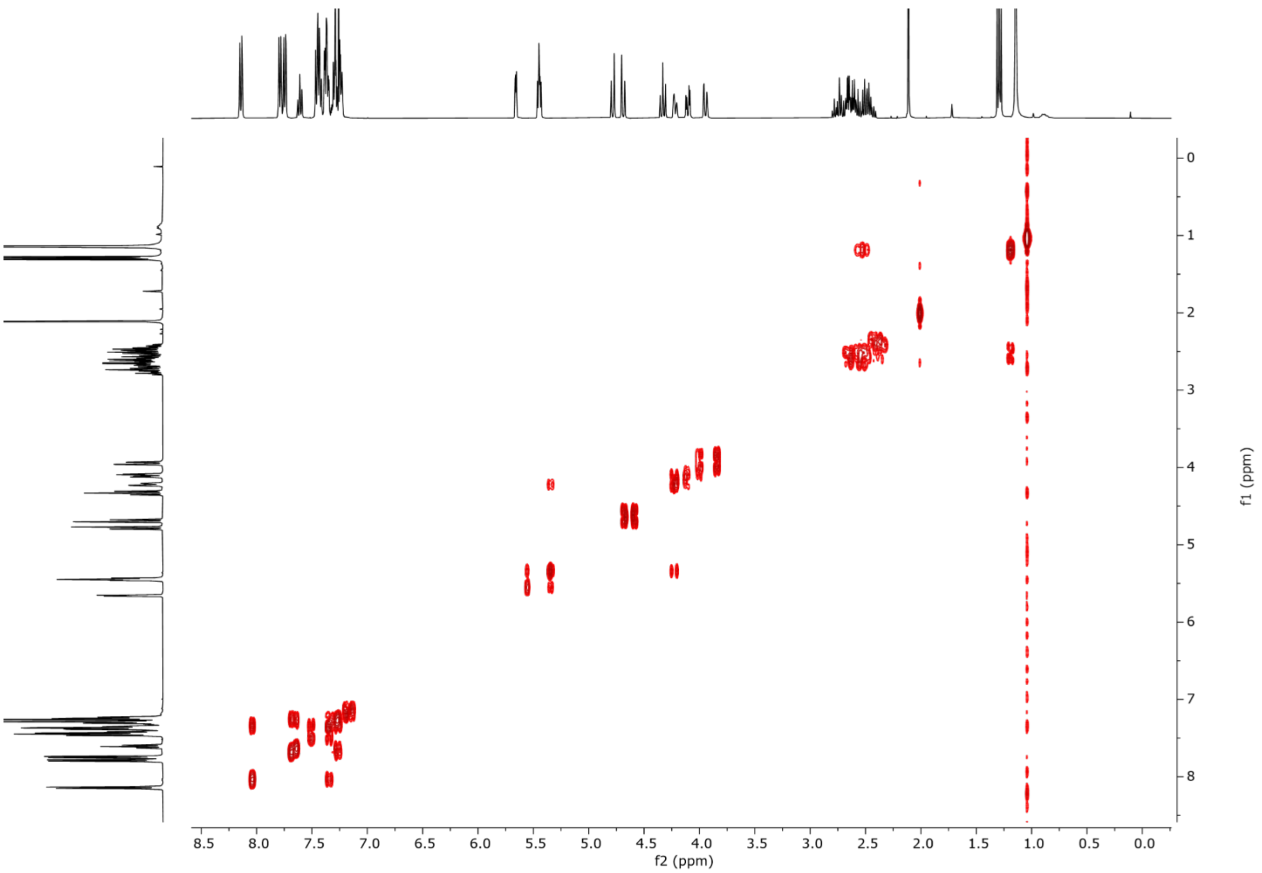

^{1}H-^{1}H COSY NMR

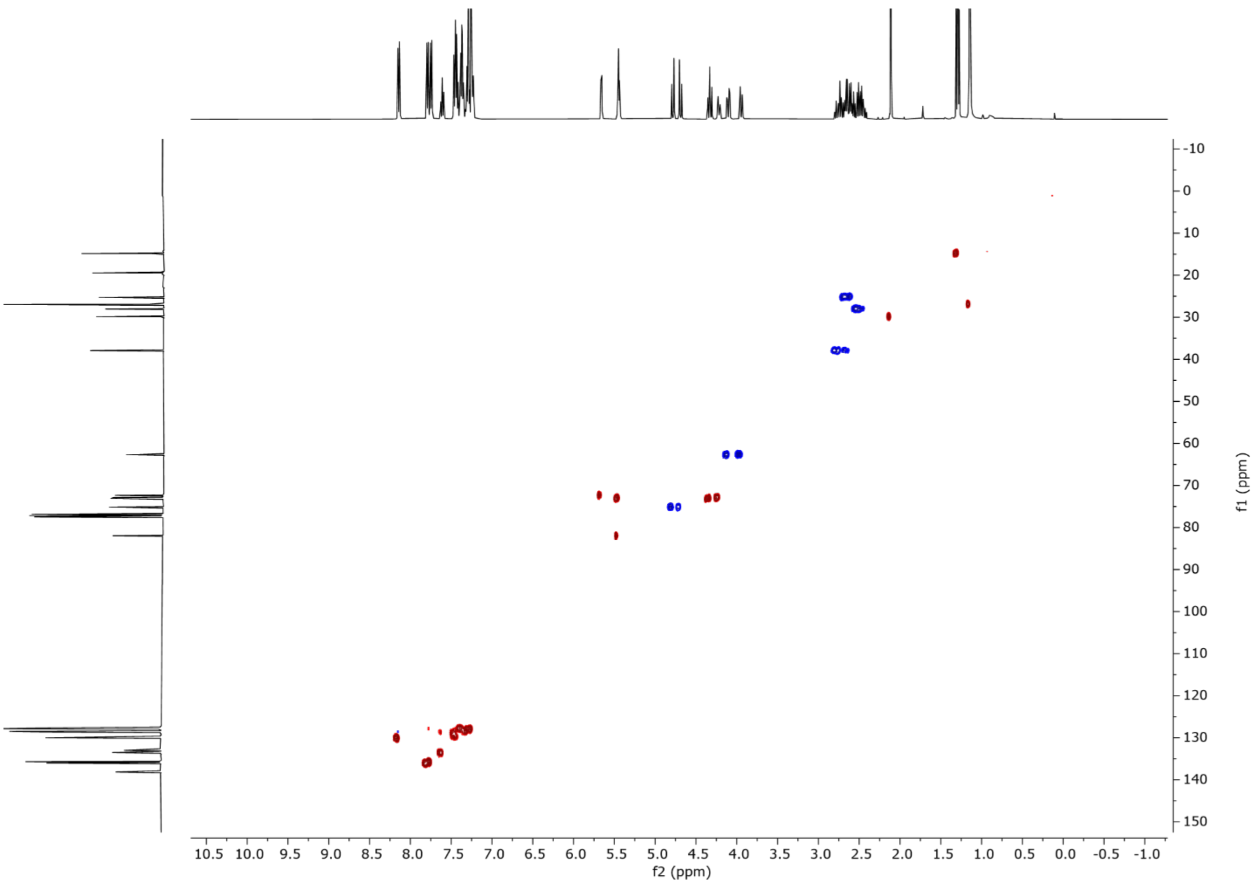

^{1}H-^{13}C HSQC NMR

Ethyl 2-*O*-benzoyl-4-*O*-benzyl-6-*O*-(9-fluorenylmethoxycarbonyl)-3-*O*-levulinoyl-1-thio-α-D-mannopyranoside (5)

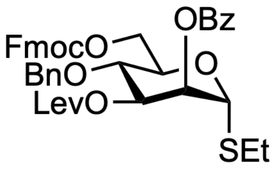

Ethyl 2-*O*-benzoyl-4-*O*-benzyl-6-*tert*-butyl diphenylsilyl-3-*O*-levulinoyl-1-thio-α-D-mannopyranoside (10.97 g, 14.53 mmol) was dissolved in pyridine (70 mL) and HF•Py (70% HF, 9.3 mL, 72.65 mmol, 5eq) was added. Once TLC analysis indicated the reaction completion (4 hours), the reaction was quenched with Et_3N (2 mL). The mixture was concentrated, dissolved in ethyl acetate and subsequently washed with saturated aqueous $NaHCO_3$ and brine. The aqueous layers were extracted with EtOAc and the combined organic layers were dried over Na_2SO_4, filtered, and concentrated *in vacuo*. The crude was passed through silica, concentrated and dried under vacuum. The intermediate residue was dissolved in anhydrous CH_2Cl_2 (75 mL), pyridine (11 mL,) and fluorenylmethoxycarbonylchloride (Fmoc-Cl) (5.64 g, 21.78 mmol, 1.5 eq) was added. The reaction was tracked by TLC analysis and once completed (16h) was quenched by the addition of 10 % aqueous citric acid solution. The aqueous layers were then extracted with CH_2Cl_2, combined organic layers were dried over Na_2SO_4, filtered and concentrated *in vacuo*. The residue was purified by flash chromatography (SiO_2, Hexane/EtOAc) to yield compound **5** (9.00 g, 84% over two steps).

^{1}H NMR (400 MHz, $CDCl_3$) δ 8.14 – 8.07 (m, 2H), 7.82 – 7.75 (m, 2H), 7.68 – 7.52 (m, 3H), 7.48 – 7.37 (m, 4H), 7.35 – 7.24 (m, 7H), 5.62 (dd, *J* = 3.2, 1.7 Hz, 1H), 5.45 – 5.37 (m, 2H), 4.77 (d, *J* = 11.2 Hz, 1H), 4.62 (d, *J* = 11.2 Hz, 1H), 4.49 (d, *J* = 3.4 Hz, 2H), 4.44 – 4.39 (m, 3H), 4.28 (d, *J* = 7.5 Hz, 1H), 4.13 – 4.08 (m, 1H), 2.83 – 2.57 (m, 4H), 2.57 – 2.37 (m, 2H), 2.11 (s, 3H), 1.29 (t, 3H). **^{13}C NMR** (101 MHz, $CDCl_3$) δ 206.3, 171.7, 165.4, 155.1, 143.4, 143.2, 141.3, 137.5, 133.6, 129.9, 129.5, 128.6, 128.5, 128.2, 128.0, 128.0, 127.2, 127.2, 125.2, 125.2, 120.1, 82.2, 77.4, 77.3, 77.1, 76.7, 74.9, 72.9, 72.7, 71.9, 70.1, 70.1, 66.5, 60.5, 46.8, 37.8, 29.8, 27.9, 25.5, 21.1, 14.9, 14.2. **HRMS** QTOF-MS: calcd. $C_{42}H_{42}NaO_{10}S$ for $[M+Na]^+$ 761.2396, found 761.2390.

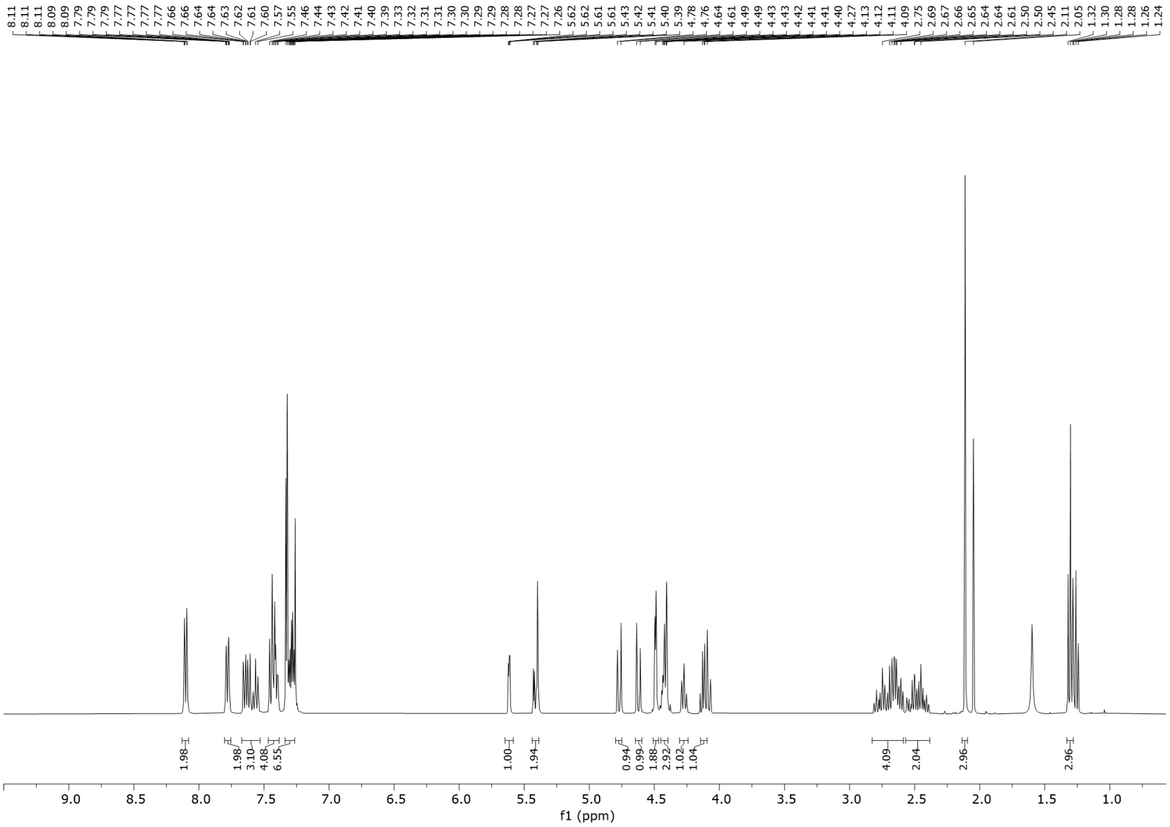

^{1}H NMR

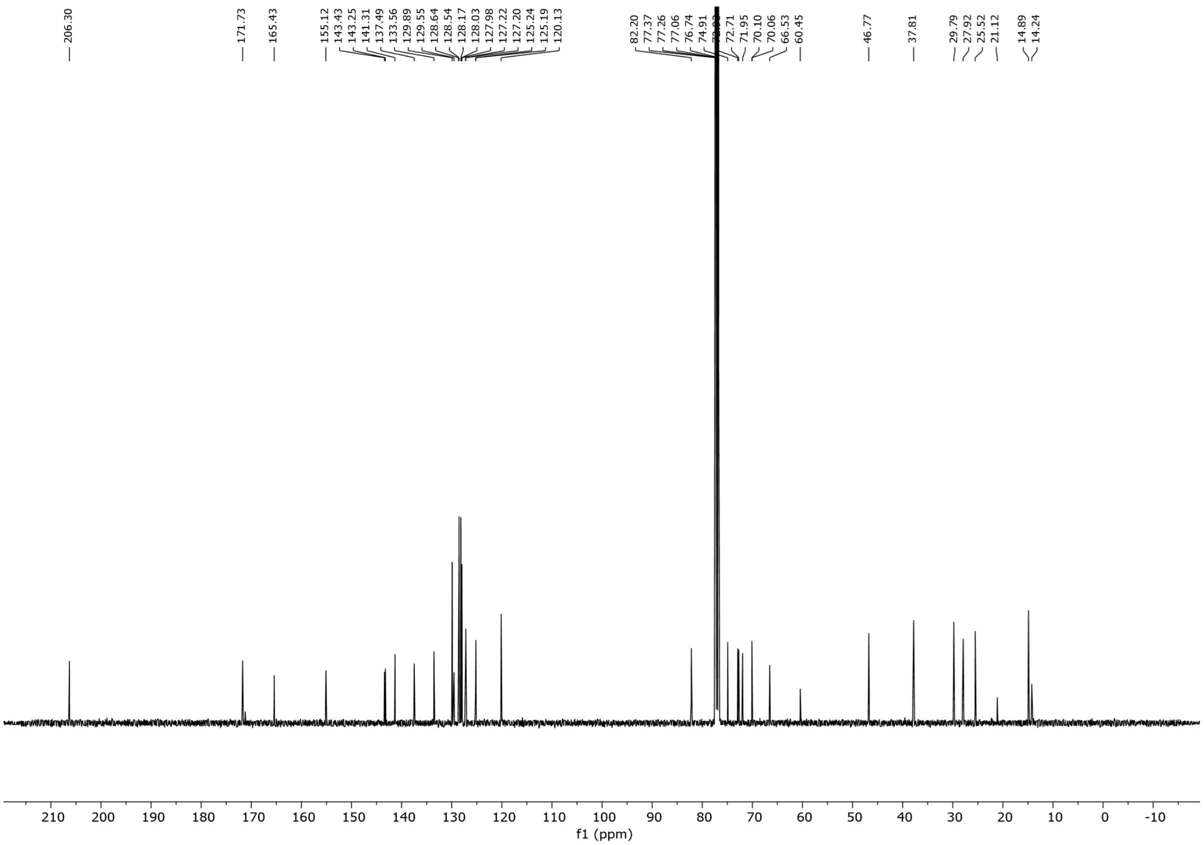
206.30
171.73
165.43
155.12
143.43
143.25
141.31
137.49
133.56
129.89
129.55
128.64
128.54
128.17
128.03
127.98
127.22
127.20
125.24
125.19
120.13
82.20
77.37
77.26
77.06
76.74
74.91
72.71
71.95
70.10
70.06
66.53
60.45
46.77
37.81
29.79
27.92
25.52
21.12
14.89
14.24
210 200 190 180 170 160 150 140 130 120 110 100 90 80 70 60 50 40 30 20 10 0 -10
f1 (ppm)

^{13}C NMR

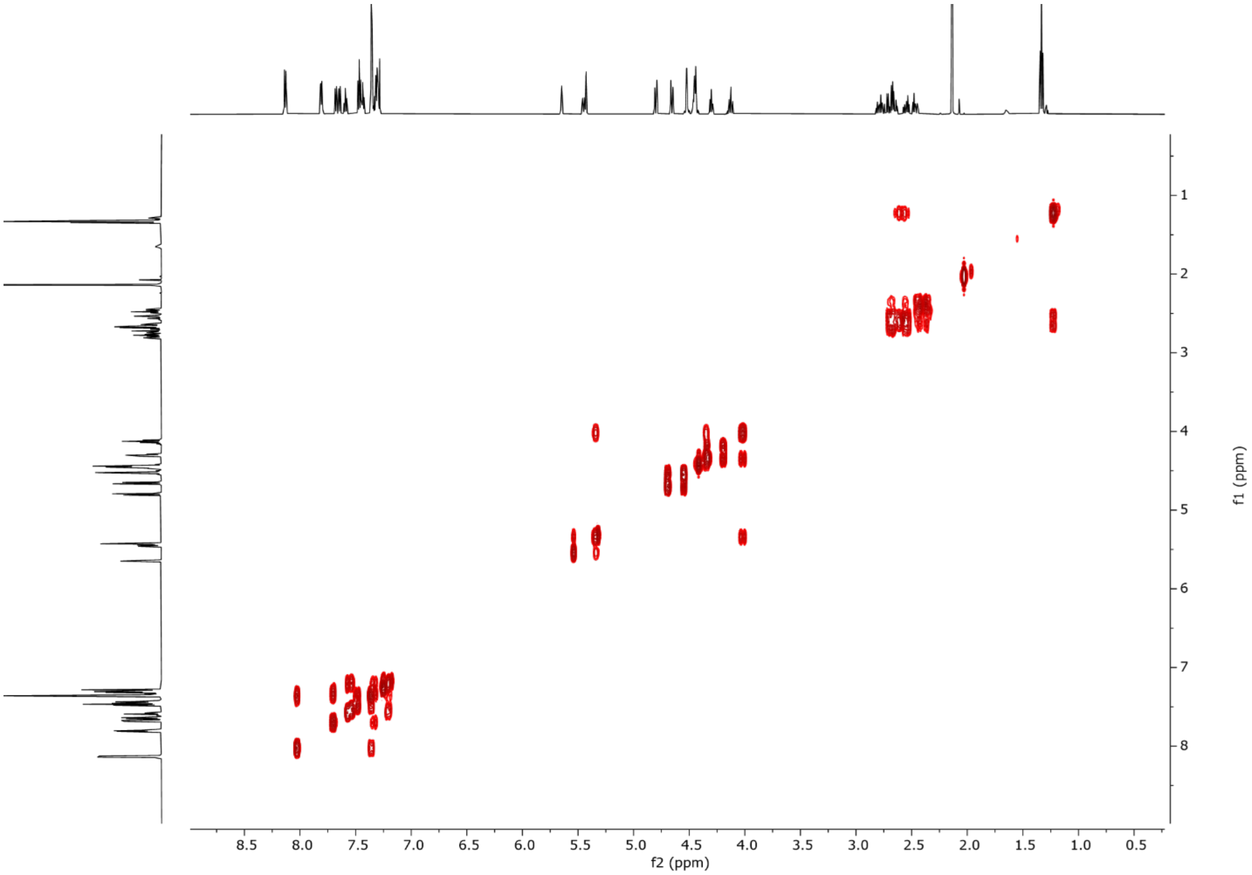

^{1}H-^{1}H COSY NMR

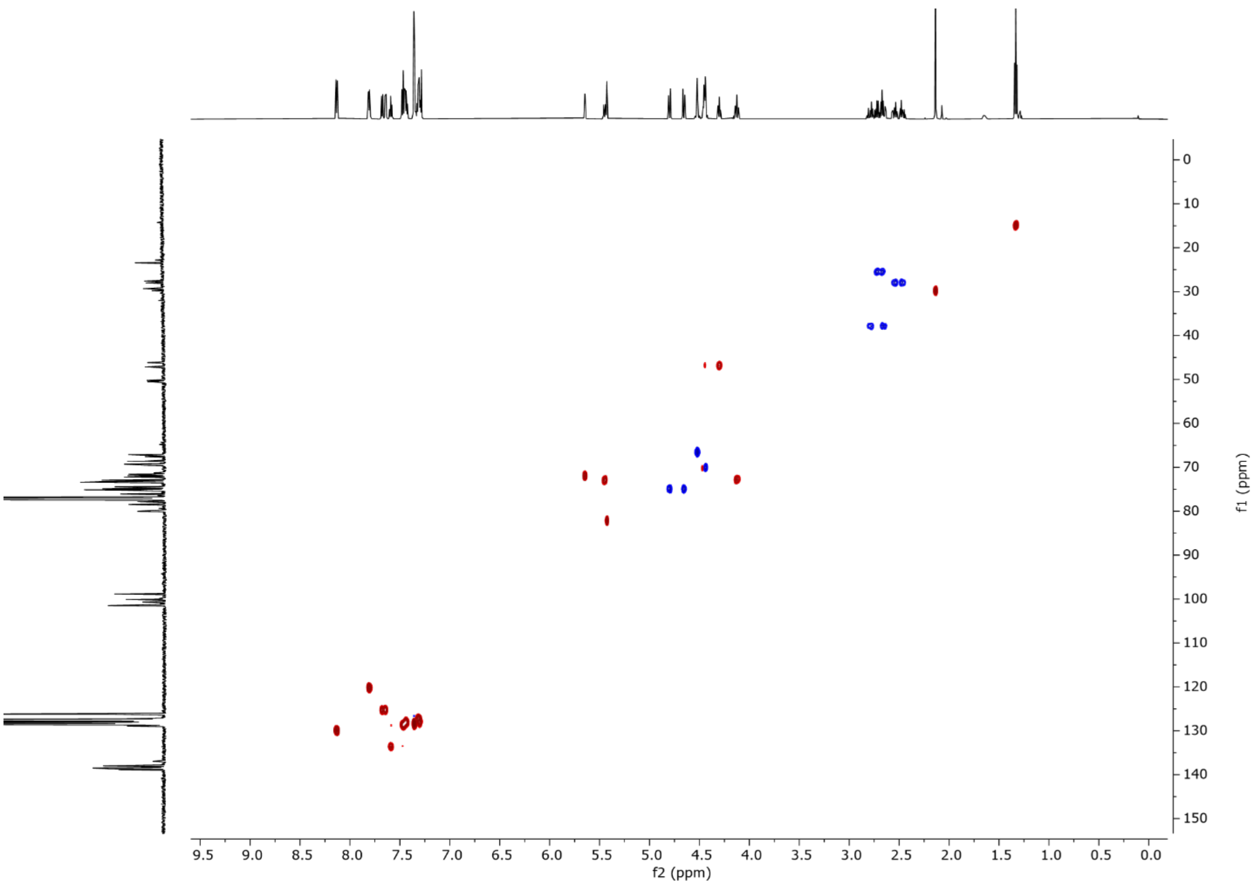

^{1}H-^{13}C HSQC NMR

Dibutyl 2-*O*-benzoyl-4-*O*-benzyl-6-*O*-(9-fluorenylmethoxycarbonyl)-*tert*-butyl diphenylsilyl-3-*O*-levulinoyl-1-thio-α-D-mannopyranoside phosphate (1)

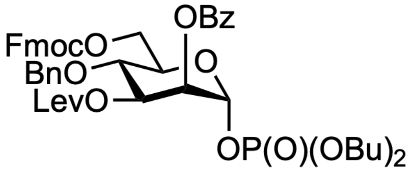

Donor **5** (2.2 g, 2.55 mmol) and dibutylphosphate (2.0 g, 3.57 mmol, 1.4 eq.) were dissolved in CH_2Cl_2 (20 mL) and 4Å molecular sieves were added. After 30 minutes, the mixture was cooled to 0 °C and *N*-iodosuccinimide (0.57 g, 2.55 mmol, 1 eq.) and TMSOTf (100 μL, 0.51 mmol, 0.2 eq.) were added. Once TLC analysis indicated complete consumption of the glycosyl donor (2 hours), the reaction was quenched with pyridine (2 mL) and diluted with DCM. After filtration through a plug of Celite®, the mixture was washed with 10 % aqueous Na_2S2O_3, saturated aqueous $NaHCO_3$, brine, and dried over Na_2SO_4 and concentrated *in vacuo*. The material was purified by flash chromatography (SiO_2, Hexane/EtOAc) to yield compound **1** (2.61 g, 98%).

^{1}H NMR (600 MHz, $CDCl_3$) δ 8.12 – 8.07 (m, 2H), 7.78 (dd, *J* = 7.6, 3.1 Hz, 2H), 7.63 (dd, *J* = 13.9, 7.5 Hz, 2H), 7.58 – 7.54 (m, 1H), 7.46 – 7.38 (m, 4H), 7.33 – 7.25 (m, 7H), 5.78 (dd, *J* = 6.7, 2.2 Hz, 1H), 5.59 (t, *J* = 2.7 Hz, 1H), 5.51 (dd, *J* = 9.5, 3.2 Hz, 1H), 4.78 (d, *J* = 11.1 Hz, 1H), 4.62 (d, *J* = 11.1 Hz, 1H), 4.52 – 4.38 (m, 4H), 4.30 – 4.19 (m, 2H), 4.12 (th, *J* = 8.1, 2.6 Hz, 5H), 2.83 – 2.74 (m, 1H), 2.68 – 2.60 (m, 1H), 2.57 – 2.49 (m, 1H), 2.48 – 2.40 (m, 1H), 2.12 (s, 3H), 1.73 – 1.63 (m, 4H), 1.47 – 1.36 (m, 4H), 0.93 (dt, *J* = 12.3, 7.4 Hz, 6H). **^{13}C NMR** (151 MHz, $CDCl_3$) δ 206.2, 171.7, 165.1, 155.0, 143.4, 143.2, 141.3, 141.3, 137.3, 133.7, 129.9, 129.1, 128.7, 128.6, 128.2, 128.1, 128.0, 127.3, 127.2, 125.2, 125.1, 120.1, 95.0, 95.0, 77.2, 77.0, 76.8, 75.1, 71.9, 71.7, 71.4, 70.1, 69.6, 69.5, 68.3, 68.2, 68.1, 68.1, 66.1, 46.8, 37.8, 32.3, 32.2, 32.2, 29.7, 27.9, 18.6, 13.6. **^{31}P NMR** (243 MHz, $CDCl_3$) δ -2.9. **HRMS** QTOF-MS: calcd. $C_{43}H_{49}O_{10}P$ for $[M+H]^+$ 772.3012, found 772.3012.

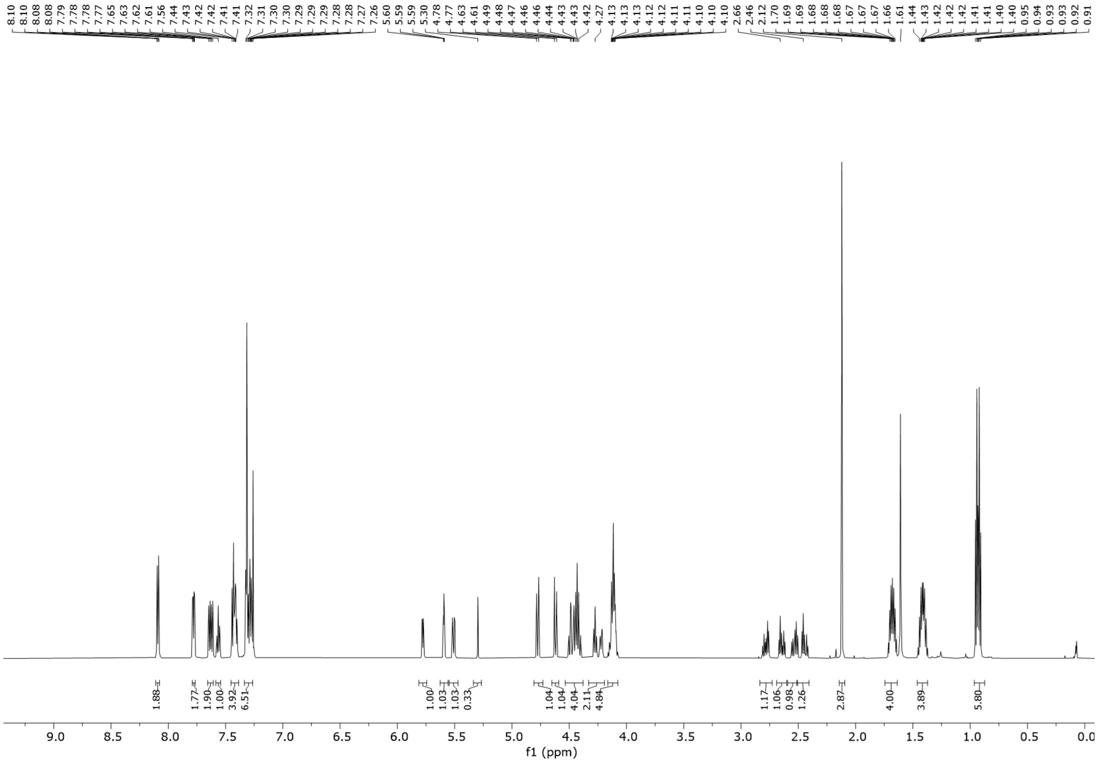

^1H NMR

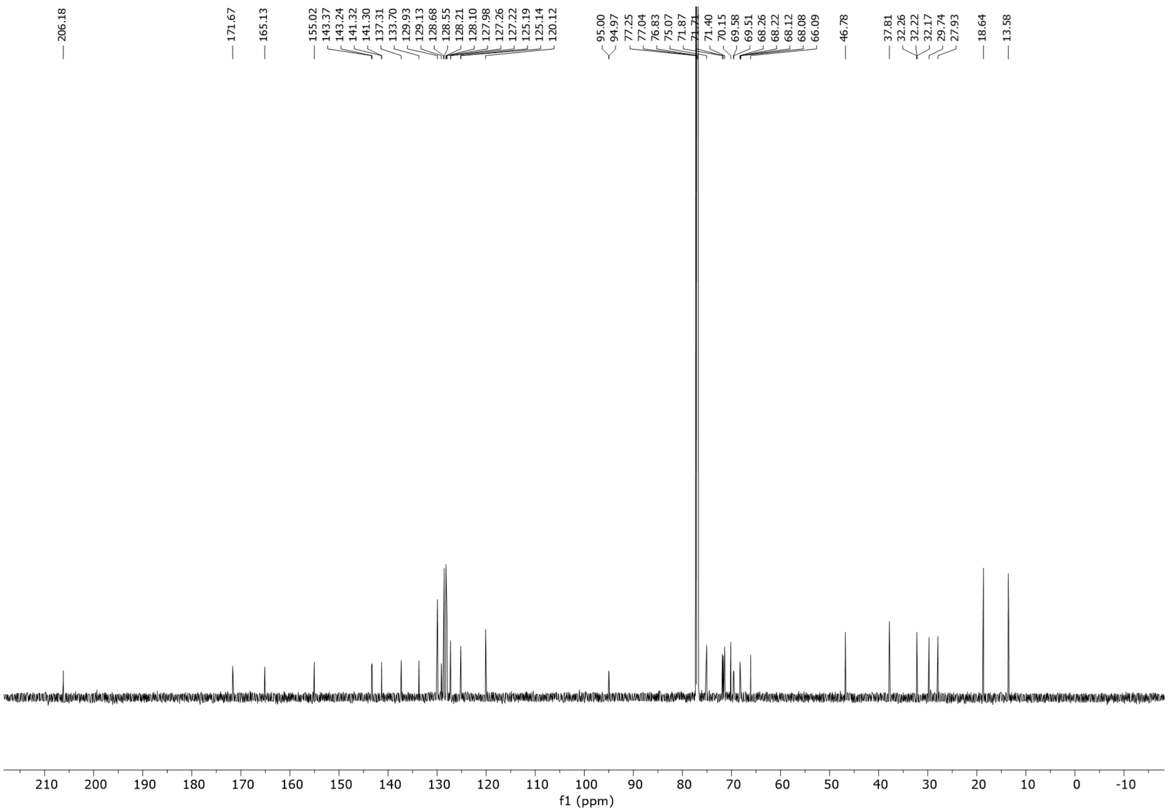

^{13}C NMR

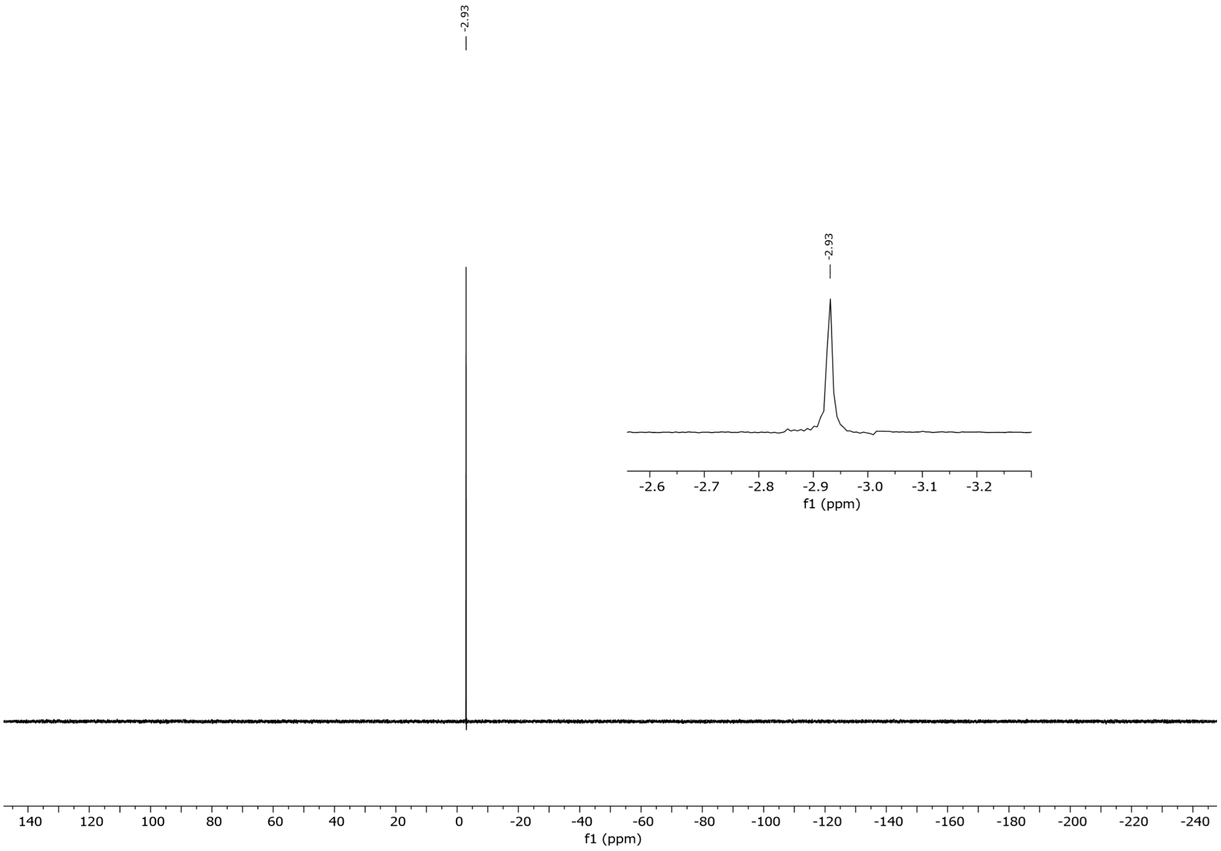
-2.93
-2.93
-2.6 -2.7 -2.8 -2.9 -3.0 -3.1 -3.2
f1 (ppm)
140 120 100 80 60 40 20 0 -20 -40 -60 -80 -100 -120 -140 -160 -180 -200 -220 -240
f1 (ppm)

^{31}P NMR

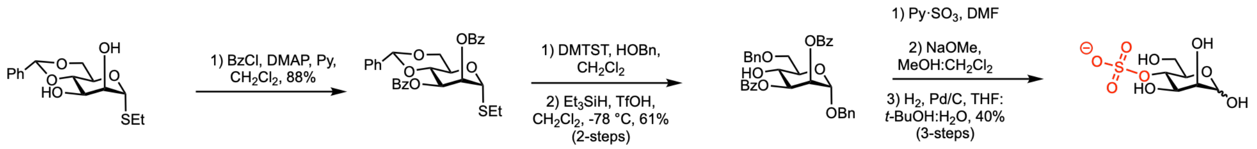

SI Scheme X. Synthesis of 4-*O*-sulfated mannose.

Ethyl 4,6-*O*-benzylidene-2,3-di-*O*- benzoyl-1-thio-α-D-mannopyranoside (6)

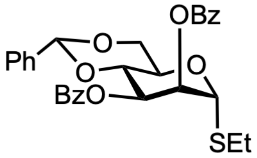

Ethyl 4,6-*O*-benzylidene-1-thio-α-D-mannopyranoside (1.5 g, 4.8 mmol, 0.2 eq.) was dissolved in CH_2Cl_2 (0.1 M, 20 mL) at 0 °C. Subsequently, benzoyl chloride (1.12 mL, 9.6 mmol, 2 eq.), pyridine (1.9 mL, 24.0 mmol, 5 eq.) and 4-dimethylaminopyridine (12 mg, 0.1 mmol, 0.2 eq.) were added. The mixture was stirred at room temperature under argon atmosphere. Once complete, as indicated by TLC analysis (≈16 h), the reaction was quenched with citric acid (10% w/v) and extracted. The organic layer was washed with saturated aqueous $NaHCO_3$, brine, dried over Na_2SO_4, and concentrated. The residue was purified by flash chromatography (SiO_2, Hexane/Ethyl acetate) to give compound **6** (2.2 g, 4.2 mmol, 88%) as white solid. R_f = 0.91 (Hexane/Ethyl acetate, 50:50, v/v).

^{1}H NMR (400 MHz, $CDCl_3$) δ 8.03 – 7.95 (m, 2H), 7.87 – 7.79 (m, 2H), 7.60 – 7.13 (m, 22H), 5.78 (dd, *J* = 10.2, 3.6 Hz, 1H), 5.67 (dd, *J* = 3.6, 1.6 Hz, 1H), 5.57 (s, 1H), 5.00 (d, *J* = 1.6 Hz, 1H), 4.72 (d, *J* = 11.9 Hz, 1H), 4.61 – 4.53 (m, 3H), 4.30 – 4.20 (m, 2H), 4.14 – 3.99 (m, 1H), 3.92 – 3.83 (m, 1H). **^{13}C NMR** (101 MHz, $CDCl_3$) δ 192.6, 165.5, 165.5, 141.0, 137.1, 136.5, 133.6, 133.1, 130.0, 129.9, 128.7, 128.7, 128.7, 128.3, 128.2, 127.7, 127.1, 126.2, 102.0, 98.0, 71.1, 69.9, 69.1, 68.9, 65.4, 64.2. **HRMS** QTOF-MS: calcd. $C_{29}H_{30}NaO_6S$ for $[M+H]^+$ 529.1661, found 529.1668.

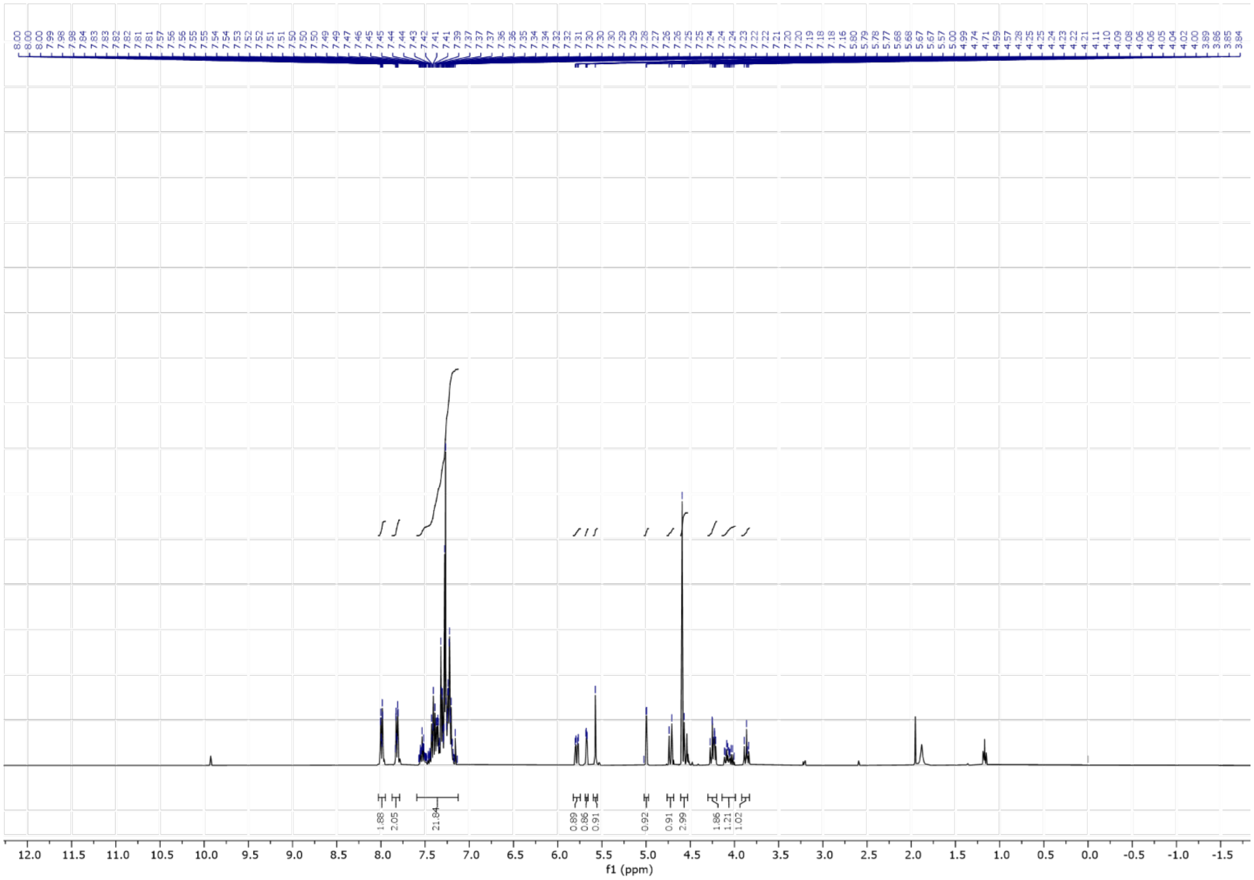

^{1}H NMR

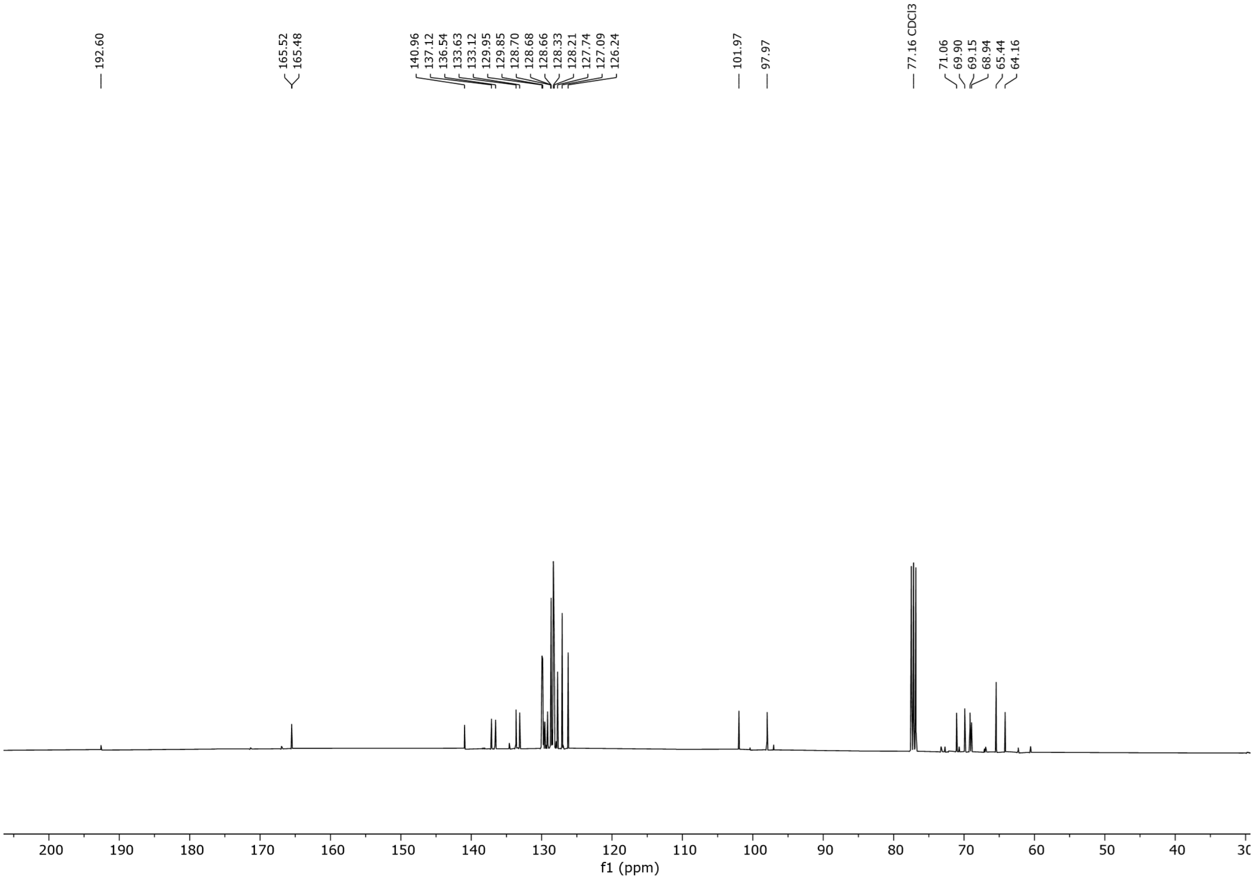
192.60
165.52
165.48
140.96
137.12
136.54
133.63
133.12
129.95
129.85
128.70
128.68
128.66
128.33
128.21
127.74
127.09
126.24
101.97
97.97
77.16 CDCl3
71.06
69.90
69.15
68.94
65.44
64.16
200 190 180 170 160 150 140 130 120 110 100 90 80 70 60 50 40 30
f1 (ppm)

^{13}C NMR

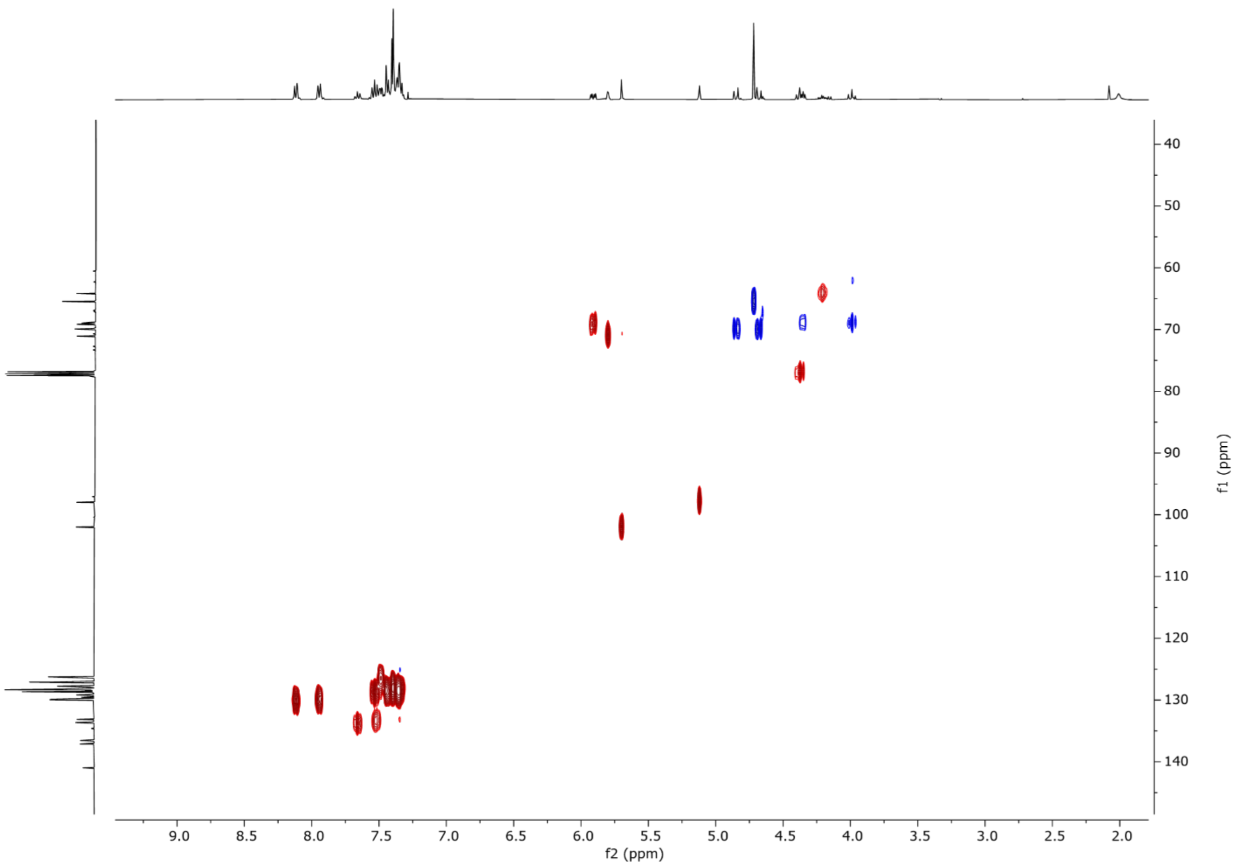

^{1}H-^{13}C HSQC NMR

Benzyl 6-*O*-benzy-2,3-di-*O*- benzoyl-1-α-D-mannopyranoside (7)

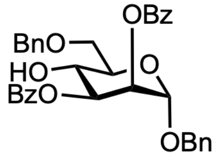

Ethyl 4,6-*O*-benzylidene-2,3-di-*O*-benzoyl-1-thio-α-D-mannopyranoside (1.5 g, 2.64 mmol) was dissolved in anhydrous CH_2Cl_2 (0.1M, 20 mL) and benzyl alcohol (0.55 mL, 5.28 mmol, 2 eq.) and 4Å molecular sieves were added. After 30 minutes, the mixture was cooled to 0 °C and DMTST (2.24 g, 8.71 mmol, 3.3 eq.) was added. Once TLC analysis indicated the reaction had reached completion it was quenched with triethylamine (2 mL), diluted with DCM, and filtered through a plug of Celite®. The solution was washed with saturated aqueous $NaHCO_3$, brine, and dried over Na_2SO_4 and concentrated. The material was purified by flash chromatography (SiO_2, Hexane/EtOAc) to yield the intermediate benzyl 4,6-*O*-benzylidene-2,3-di-*O*-benzoyl-1-α-D-mannopyranoside. The intermediate compound was dissolved in dissolved in anhydrous CH_2Cl_2 (0.2 M, 208 mL) and 4Å molecular sieves were added. The mixture was stirred at room temperature under an argon atmosphere for 20 minutes. Subsequently, the mixture was cooled to -78 °C, and triethylsilane (1.26 mL, 7.92 mmol, 3 eq.) and triflic acid (0.7 mL, 7.92 mmol, 3 eq.) was added. The reaction was maintained at -78 °C until the complete, as indicated by TLC analysis (20 minutes). The reaction was neutralized with Et_3N (1 mL) and the mixture was filtered through a pad of Celite®. The filtrate was washed with saturated aqueous $NaHCO_3$, brine, dried over Na_2SO_4, and concentrated. The residue was purified by flash chromatography (SiO_2, Hexane/Ethyl acetate) to give compound **7** (0.91 g, 1.61 mmol, 61%) as white solid.

^{1}H NMR (400 MHz, $CDCl_3$) δ 8.14 – 8.04 (m, 2H), 8.00 – 7.91 (m, 2H), 7.68 – 7.23 (m, 19H), 5.72 – 5.66 (m, 2H), 5.14 (d, *J* = 1.5 Hz, 1H), 4.84 (d, *J* = 11.9 Hz, 1H), 4.79 – 4.62 (m, 4H), 4.48 – 4.39 (m, 1H), 4.10 – 4.01 (m, 1H), 4.01 – 3.94 (m, 1H), 3.88 (dd, *J* = 10.5, 3.6 Hz, 1H). **^{13}C NMR** (101 MHz, $CDCl_3$) δ 166.8, 165.6, 138.2, 136.8, 133.5, 133.4, 130.0, 129.9, 129.6, 129.5, 128.7, 128.6, 128.6, 128.5, 128.4, 128.2, 128.2, 128.1, 127.8, 127.7, 127.7, 127.1, 97.0, 77.5, 77.2, 76.8, 73.9, 73.1, 71.8, 70.6, 69.9, 69.7, 67.6. **HRMS** QTOF-MS: calcd. $C_{34}H_{32}NaO_8$ for $[M+Na]^+$ 591.1995, found 591.1989.

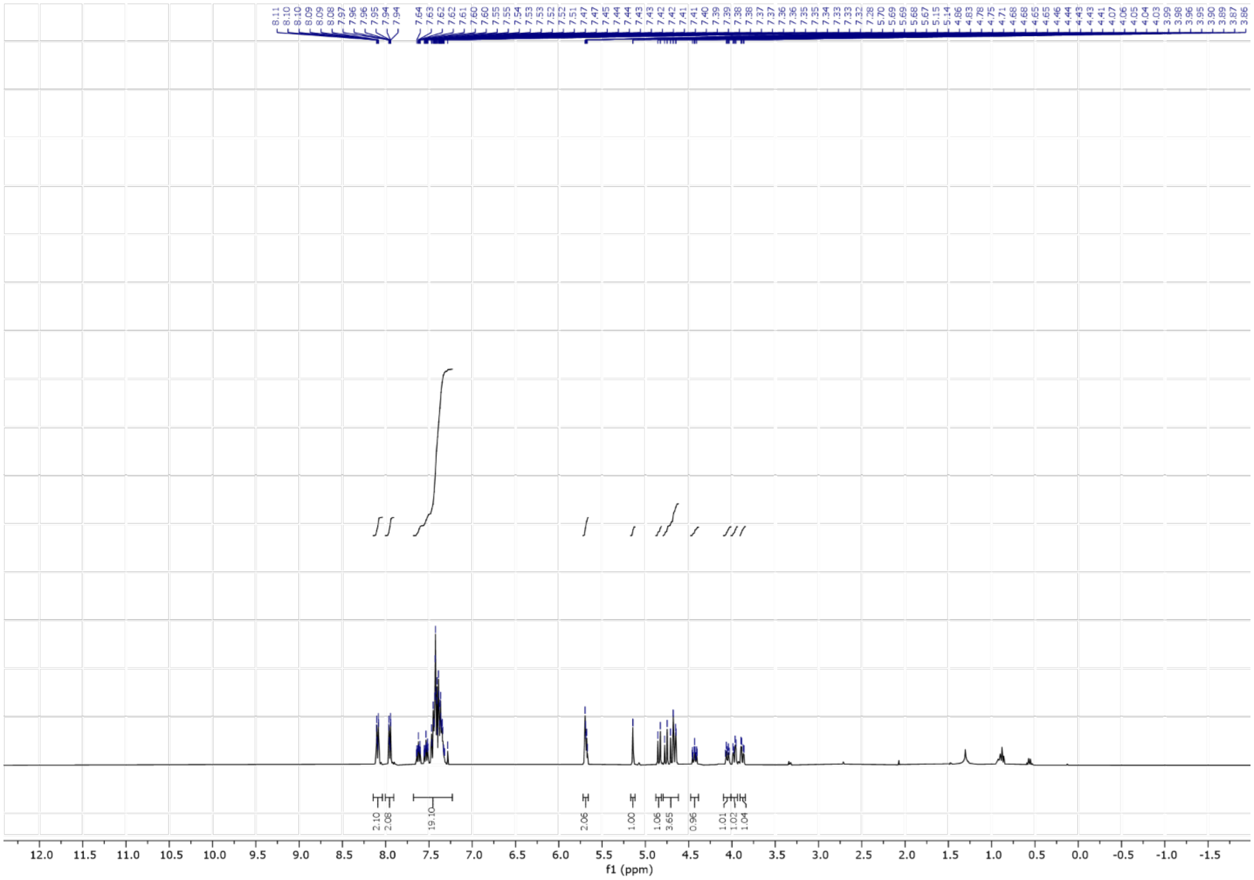

^{1}H NMR

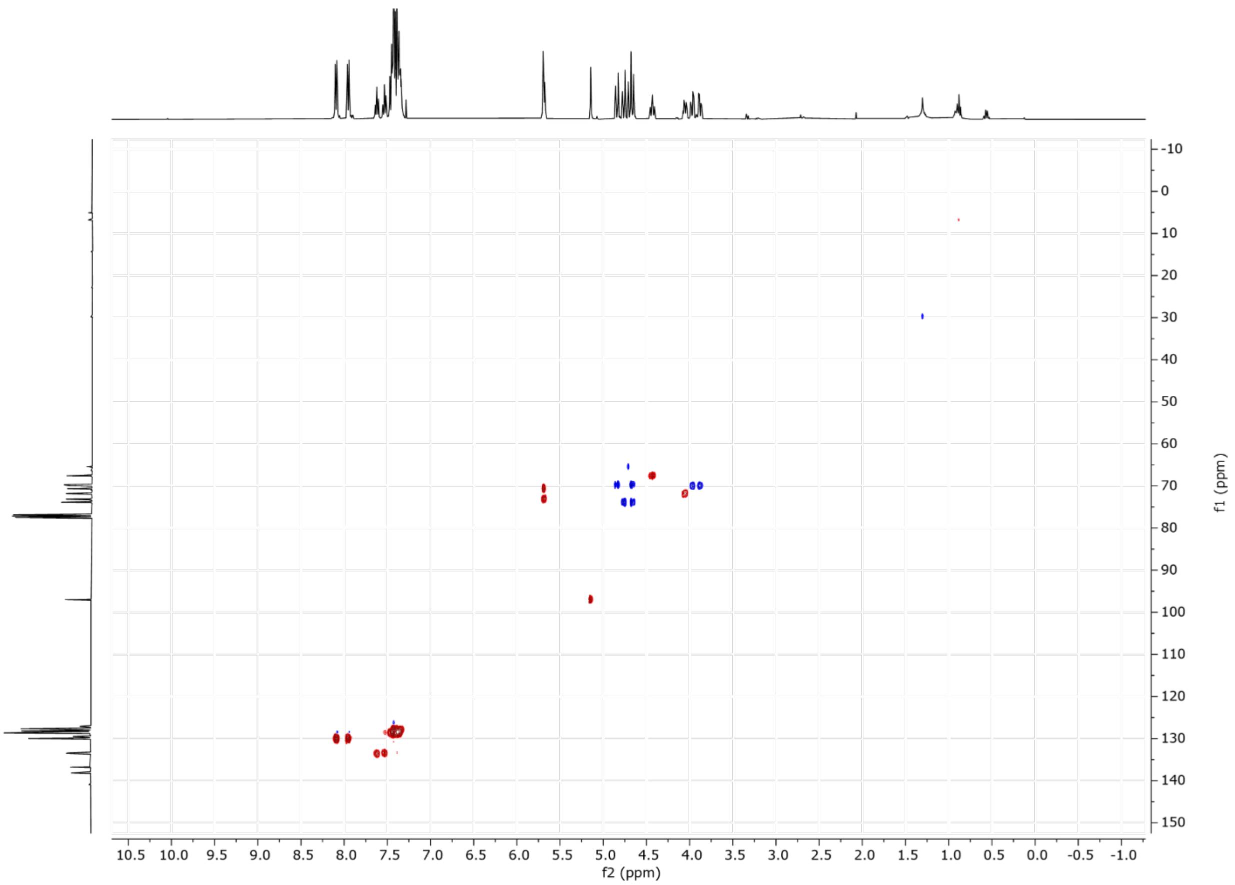

f2 (ppm)
f1 (ppm)

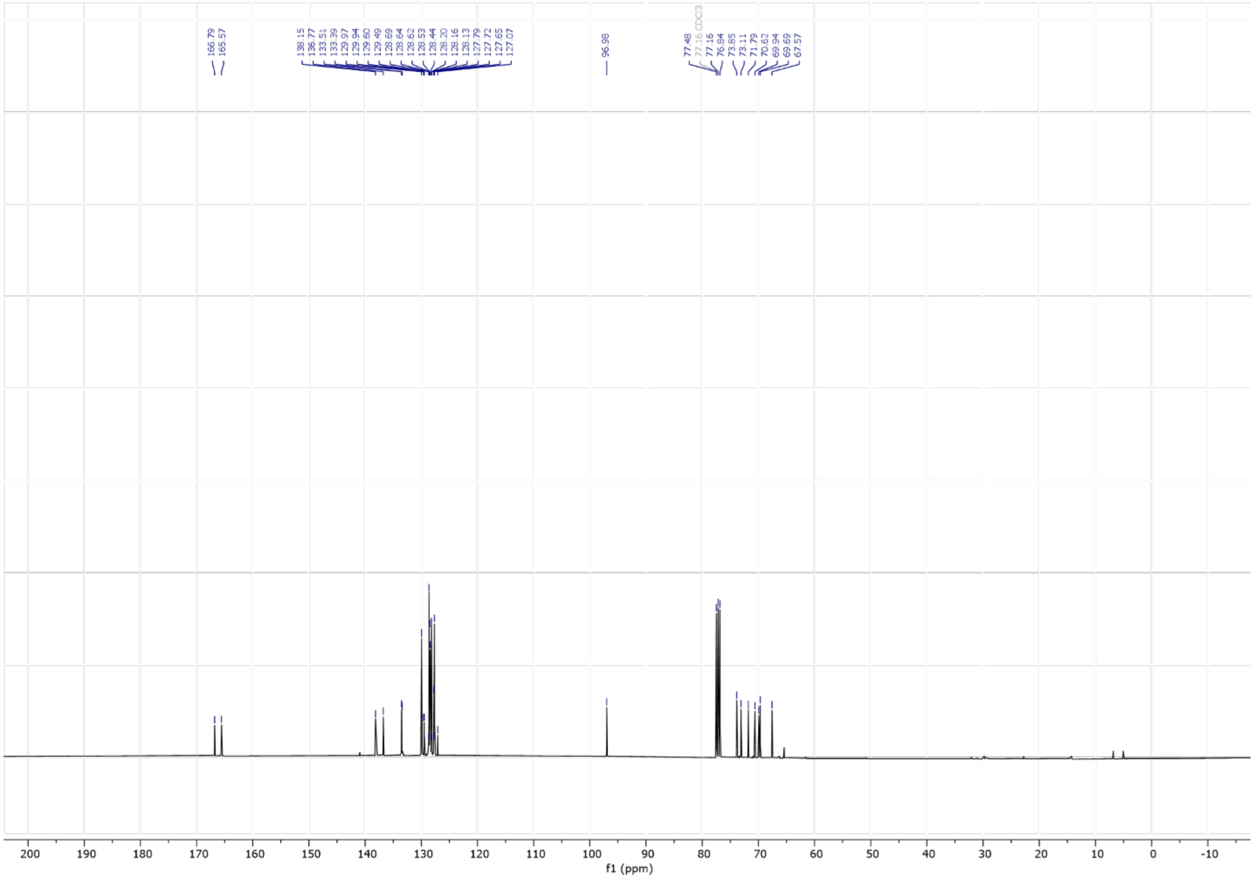

^{13}C NMR

^{1}H-^{13}C HSQC NMR

4-*O*-sulfonate-D-mannopyranose (8)

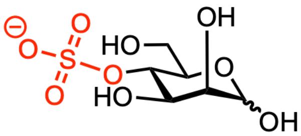

Benzyl 6-*O*-benzy-2,3-di-*O*-benzoyl-1-α-D-mannopyranoside (80 mg, 0.141 mmol) was dissolved in DMF (2 mL) and Py·SO_3 (140 mg, 0.70 mmole, 5 eq.) was added. The reaction was stirred at room temperature until completion, as indicated by TLC (16 h). A premixed solution of Et_3N/MeOH (1/1, v/v, 1.0 mL) was added to quench the reaction and stirring was continued for another 30 mins. The reaction mixture was concentrated and purified by flash chromatography (SiO_2, MeOH/ CH_2Cl_2) to give the intermediate benzyl 6-*O*-benzy-2,3-di-*O*-benzoyl-4-*O*-sulfonate-1-α-D-mannopyranoside. This intermediate was then dissolved in THF: *t*BuOH: H_2O (4 mL, 50:20:30, v/v/v) and 5% Pd-C (200 mg) was added. The reaction was stirred under H_2 atmosphere for 12 h. The reaction was filtered through a pad of Celite® and washed with *t*BuOH and H_2O. The filtrates were concentrated *in vacuum*, and dissolved in 3.0 mL of water for RP-HPLC purification (Method 6) to yield **8** (16 mg, 0.063 mmol, 40%).

^{1}H NMR (400 MHz, D_2O) δ 5.12 (s, 1H), 4.86 (s, 1H), 4.32 (t, *J* = 9.5 Hz, 1H), 4.25 (t, *J* = 9.7 Hz, 0H), 3.98 (dd, *J* = 9.2, 3.3 Hz, 1H), 3.96 – 3.85 (m, 3H), 3.85 – 3.76 (m, 1H), 3.76 – 3.66 (m, 2H), 3.50 – 3.43 (m, 0H). **^{13}C NMR** (101 MHz, D_2O) δ 93.5, 75.2, 74.9, 74.4, 71.7, 71.1, 70.6, 70.6, 69.0, 60.6. **HRMS** QTOF-MS: calcd. $C_6H_{11}O_8S$ for $[M-H]^-$ 260.0202, found 260.0209.

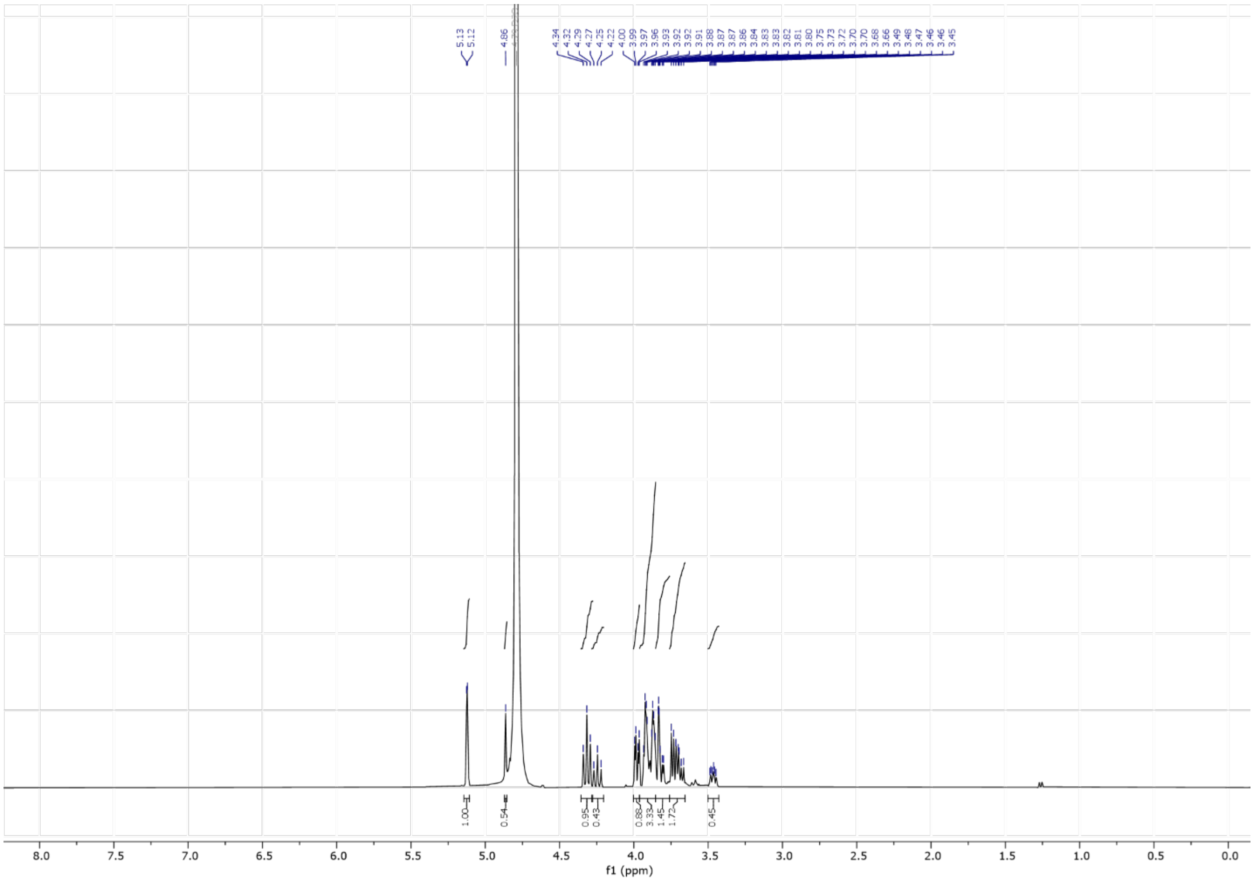

^{1}H NMR

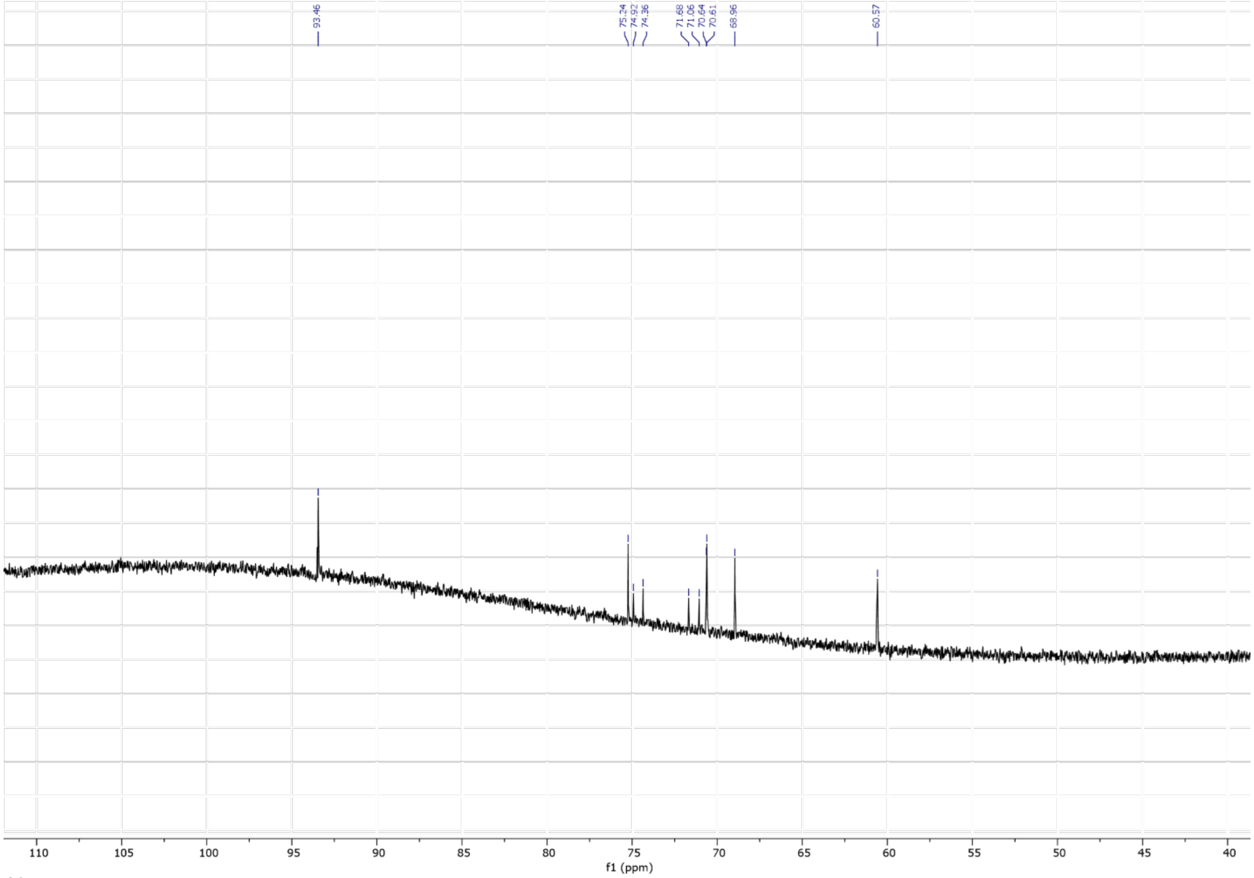

^{13}C NMR

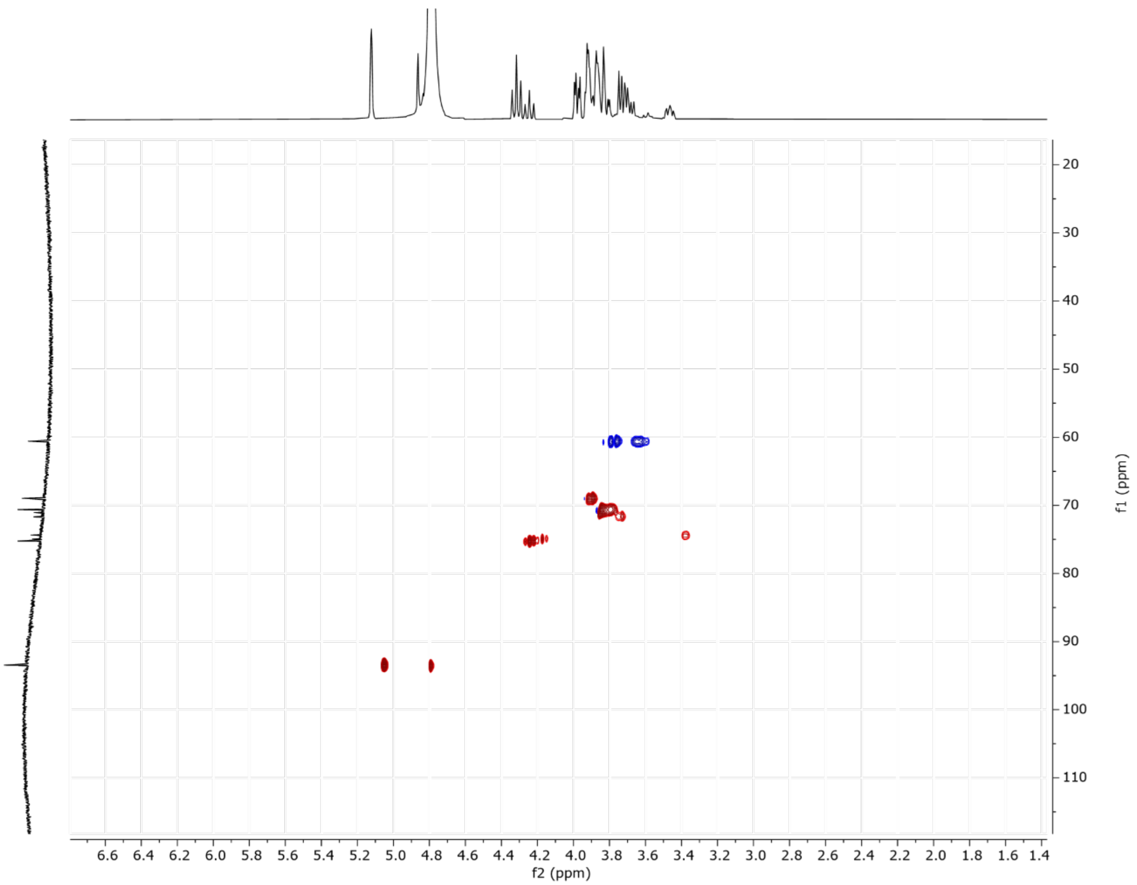

^{1}H-^{13}C HSQC NMR

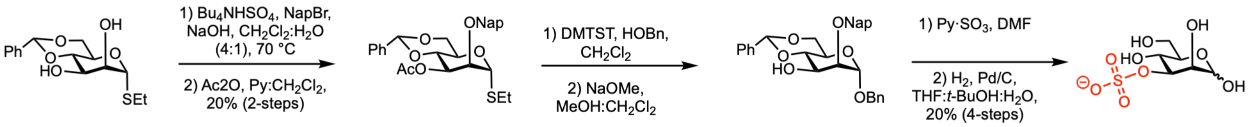

Scheme 1. Synthesis of 3-*O*-sulfated mannose.

Ethyl 4,6-*O*-benzylidene-3-*O*-acetyl-2-*O*-(2-naphthalenylmethyl)-1-thio-α-D-mannopyranoside (9)

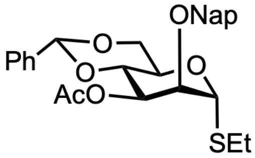

Ethyl 4,6-*O*-benzylidene-1-thio-α-D-mannopyranoside (1.0 g, 3.2 mmol) was dissolved in CH_2Cl_2 (50 mL) and Bu_4NHSO_4 (0.2 g, 0.6 mmol, 0.2 equiv.) and 2-(bromomethyl)naphthalene (0.78 g, 3.5 mmol, 1.1 eq.) were added. Subsequently, an aqueous solution of NaOH (4.0 g in 50 mL) was added and the biphasic solution was refluxed at 70 °C for 16h. The mixture was allowed to cool down to room temperature and the aqueous phase was extracted with CH_2Cl_2. The combined organic phase was washed with a 10% citric acid solution, dried over Na_2SO_4, filtered, and concentrated. The intermediate ethyl 4,6-*O*-benzylidene-2-*O*-(2-naphthalenylmethyl)-1-thio-α-D-mannopyranoside was isolated after purification by column chromatography (SiO_2, Hex/EtOAc). The intermediate was dissolved in CH_2Cl_2 (0.1 M, 35 mL) and acetic anhydride (0.6 mL, 6.4 mmol, 2 eq.), pyridine (1.3 mL, 16.0 mmol, 5 eq.) and 4-dimethylaminopyridine (78 mg, 0.64 mmol, 0.2 eq.) were added. The mixture was stirred at room temperature. Once complete, as indicated by TLC analysis (16 h), the reaction was quenched with citric acid (10% w/v) and extracted. The organic layer was washed with saturated aqueous $NaHCO_3$, brine, dried over Na_2SO_4, and concentrated. The residue was purified by flash chromatography (SiO_2, Hexane/Ethyl acetate) to give compound **9** (0.3 g, 0.64 mmol, 20% over 2 steps) as white solid.

^{1}H NMR (400 MHz, $CDCl_3$) δ 7.83 – 7.69 (m, 4H), 7.50 – 7.34 (m, 5H), 7.33 – 7.21 (m, 3H), 5.51 (s, 1H), 5.30 (s, 1H), 5.17 (dd, *J* = 10.2, 3.5 Hz, 1H), 4.78 (d, *J* = 12.1 Hz, 1H), 4.66 (d, *J* = 12.1 Hz, 1H), 4.30 – 4.11 (m, 3H), 4.08 – 4.02 (m, 1H), 3.83 (t, *J* = 10.1 Hz, 1H), 3.09 (s, 2H), 2.59 – 2.45 (m, 3H), 1.90 (s, 3H), 1.16 (t, *J* = 7.4 Hz, 3H). **^{13}C NMR** (101 MHz, $CDCl_3$) δ 170.3, 137.4, 135.0, 133.3, 133.2, 129.2, 128.5, 128.4, 128.0, 127.8, 127.1, 126.5, 126.4, 126.3, 126.0, 101.9, 83.1, 78.0, 76.6, 73.5, 70.8, 68.7, 64.6, 25.5, 23.1, 21.1, 14.9. **HRMS** QTOF-MS: calcd. $C_{28}H_{30}NaO_6S$ for $[M+Na]^+$ 517.1661, found

517.1654.

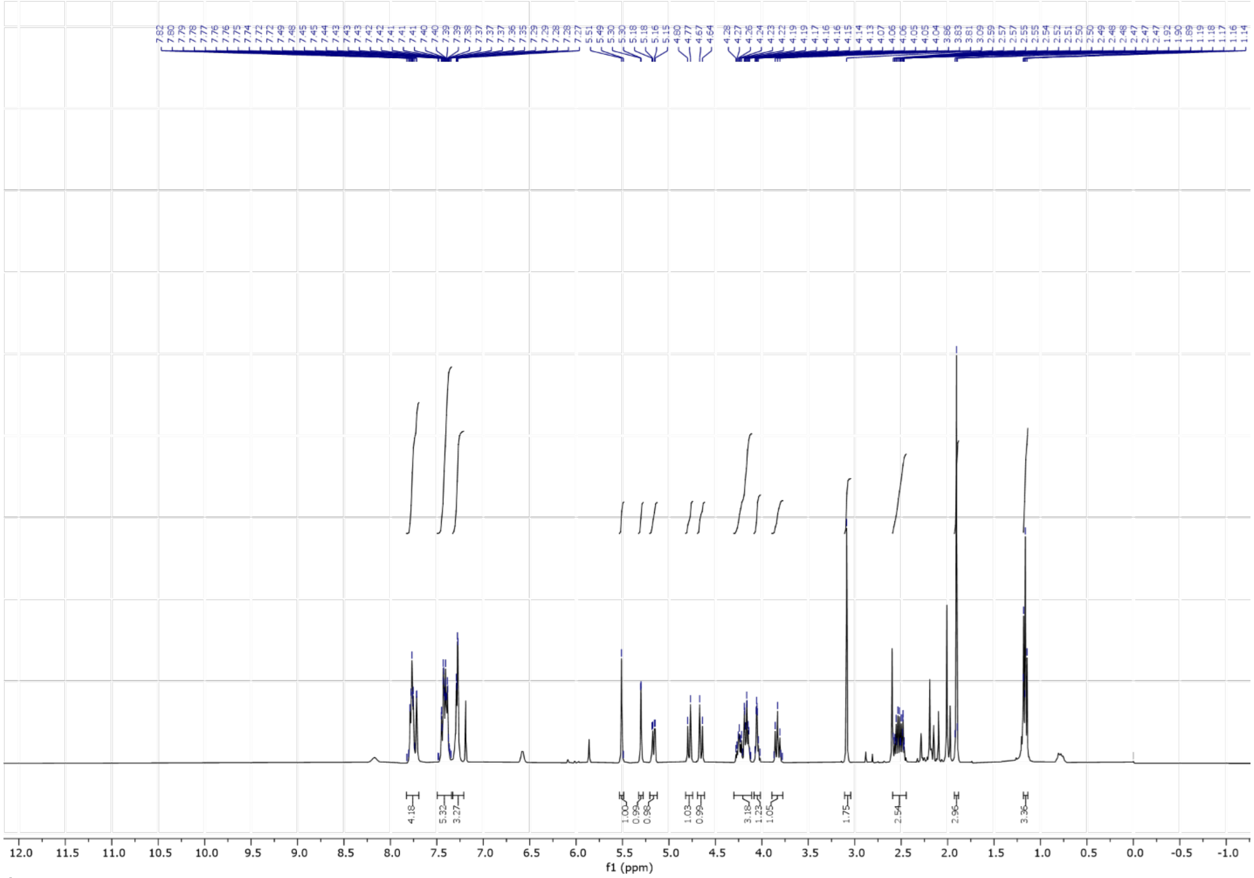

^{1}H NMR

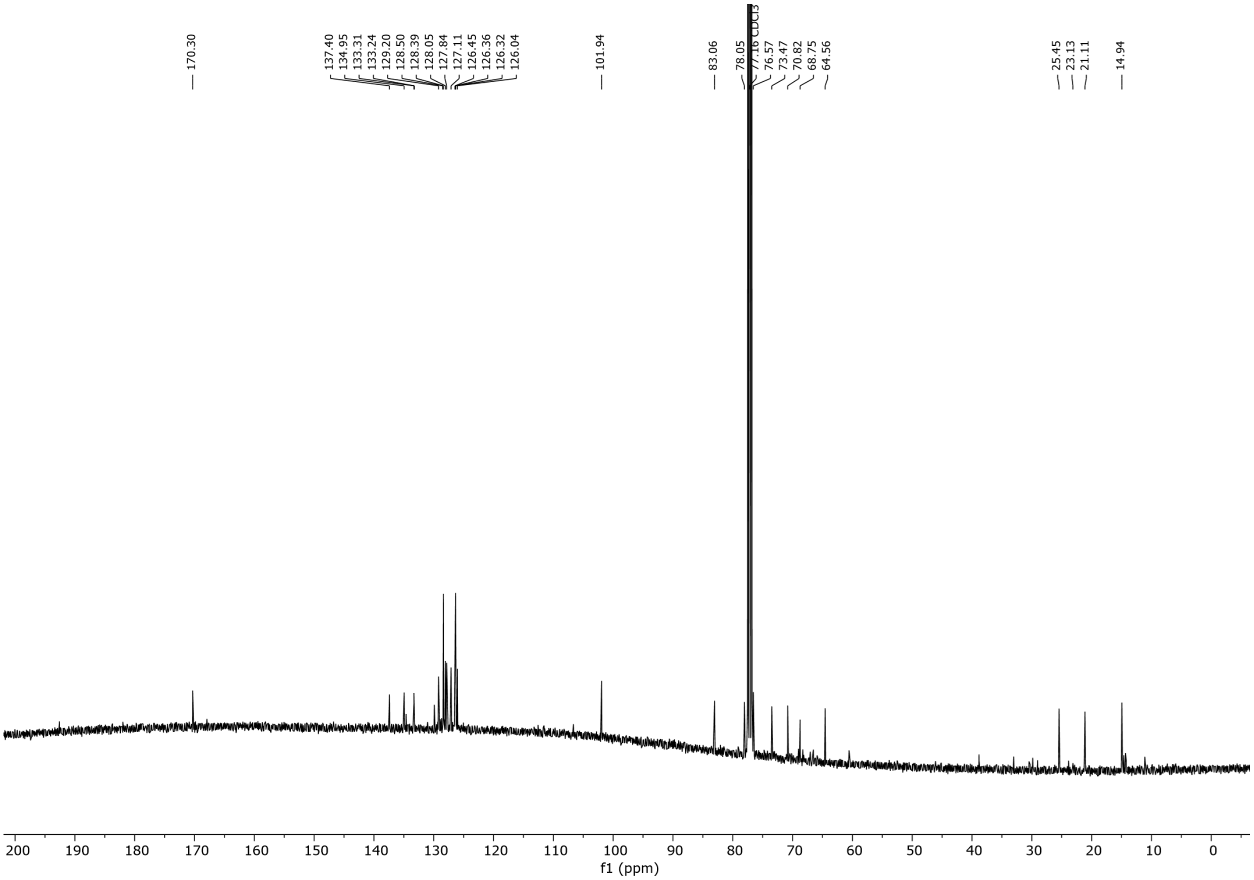
170.30
137.40
134.95
133.31
133.24
129.20
128.50
128.39
128.05
127.84
127.11
126.45
126.36
126.32
126.04
101.94
83.06
78.05
77.16 CDCl3
76.57
73.47
70.82
68.75
64.56
25.45
23.13
21.11
14.94
200 190 180 170 160 150 140 130 120 110 100 90 80 70 60 50 40 30 20 10 0
f1 (ppm)

^{13}C NMR

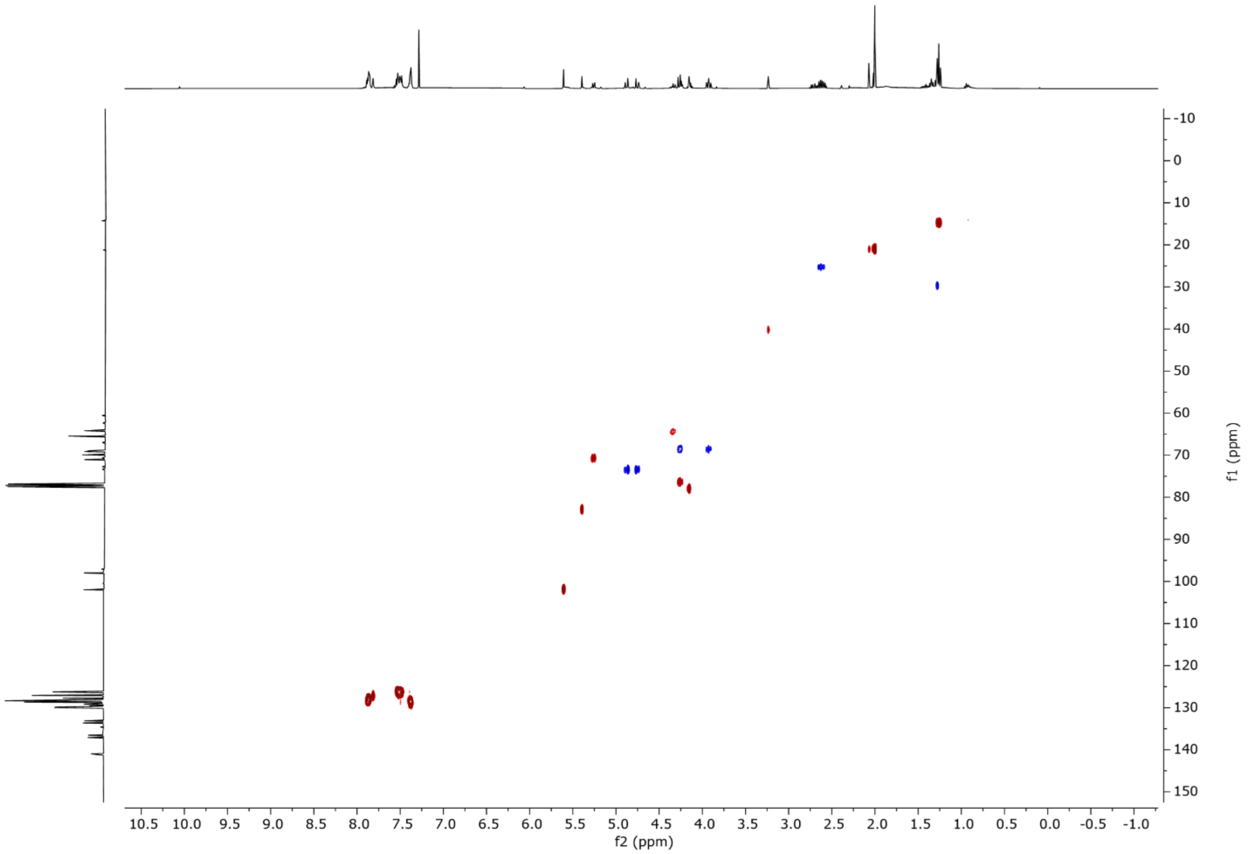

^{1}H-^{13}C HSQC

3-*O*-sulfonate-D-mannopyranose (10)

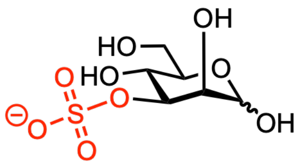

Ethyl 4,6-*O*-benzylidene-3-*O*-acetyl-2-*O*-(2-naphthalenylmethyl)-1-thio-α-D-mannopyranoside (0.3 g, 0.60 mmol) was dissolved in anhydrous CH_2Cl_2 (0.1M, 6 mL) and benzyl alcohol (0.18 mL, 1.82 mmol, 3eq) and 4Å molecular sieves were added. After 30 minutes, the mixture was cooled to 0 °C and DMTST (0.4 g, 1.82 mmol, 3 eq.) was added. Once TLC analysis indicated the reaction had reached completion it was quenched with triethylamine (3 mL), diluted with DCM, and filtered through a plug of Celite®. The solution was washed with saturated aqueous $NaHCO_3$, brine, and dried over Na_2SO_4 and concentrated. The material was purified by flash chromatography (SiO_2, Hexane/EtOAc) to yield the intermediate benzyl 4,6-*O*-benzylidene-3-*O*-acetyl-2-*O*-(2-naphthalenylmethyl)-1-thio-α-D-mannopyranoside. The intermediate was dissolved in CH_2Cl_2 (0.1M, 6 mL) and the pH was raised to pH 8 with a sodium methoxide (approx. 0.1 mL, 0.5M solution). Once complete, the reaction was quenched by the addition of Amberlite™ (Hydrogen foam), filtered, and concentrated under vacuum. The crude material was then dissolved in DMF (6 mL) and Py·SO_3 (5 eq) was added and stirred at room temperature. Upon completion of reaction as indicated by TLC (16 h), a premixed solution of Et_3N/MeOH (1/1, v/v, 1.0 mL) was added to quench the reaction and stirring was continued for another 30 mins. The reaction mixture was concentrated and purified by flash chromatography (SiO_2, MeOH/ CH_2Cl_2) to give the intermediate benzyl 4,6-*O*-benzylidene-3-*O*-sulfonate-2-*O*-(2-naphthalenylmethyl)-1-thio-α-D-mannopyranoside.
This intermediate was then dissolved in THF: *t*BuOH: H_2O (4 mL, 50:20:30) and 5% Pd-C (200 mg) was added. The reaction was stirred under H_2 atmosphere for 12 h. The reaction was filtered through a pad of celite and washed with *t*BuOH and H_2O. The filtrates were concentrated *in vacuum*, and dissolved in 3.0 mL of water for RP-HPLC purification (Method 6) to yield **10** (31 mg, 0.12 mmol, 20%).

^{1}H NMR (400 MHz, D_2O) δ 5.13 (d, *J* = 2.0 Hz, 1H), 4.89 (d, *J* = 1.1 Hz, 0H), 4.47 (dd, *J* = 9.5, 3.2 Hz, 1H), 4.30 – 4.26 (m, 0H), 4.21 (dd, *J* = 3.3, 2.0 Hz, 1H), 3.89 – 3.62 (m, 4H), 3.41 (ddd, *J* = 9.9, 6.0, 2.3 Hz, 0H). **^{13}C NMR** (101 MHz, D_2O) δ 93.9, 93.4, 78.9, 72.4, 69.2, 64.7, 60.8. **HRMS** QTOF-MS: calcd. $C_6H_{11}O_9S$ for $[M-H]^-$ 259.0129, found 259.0134.

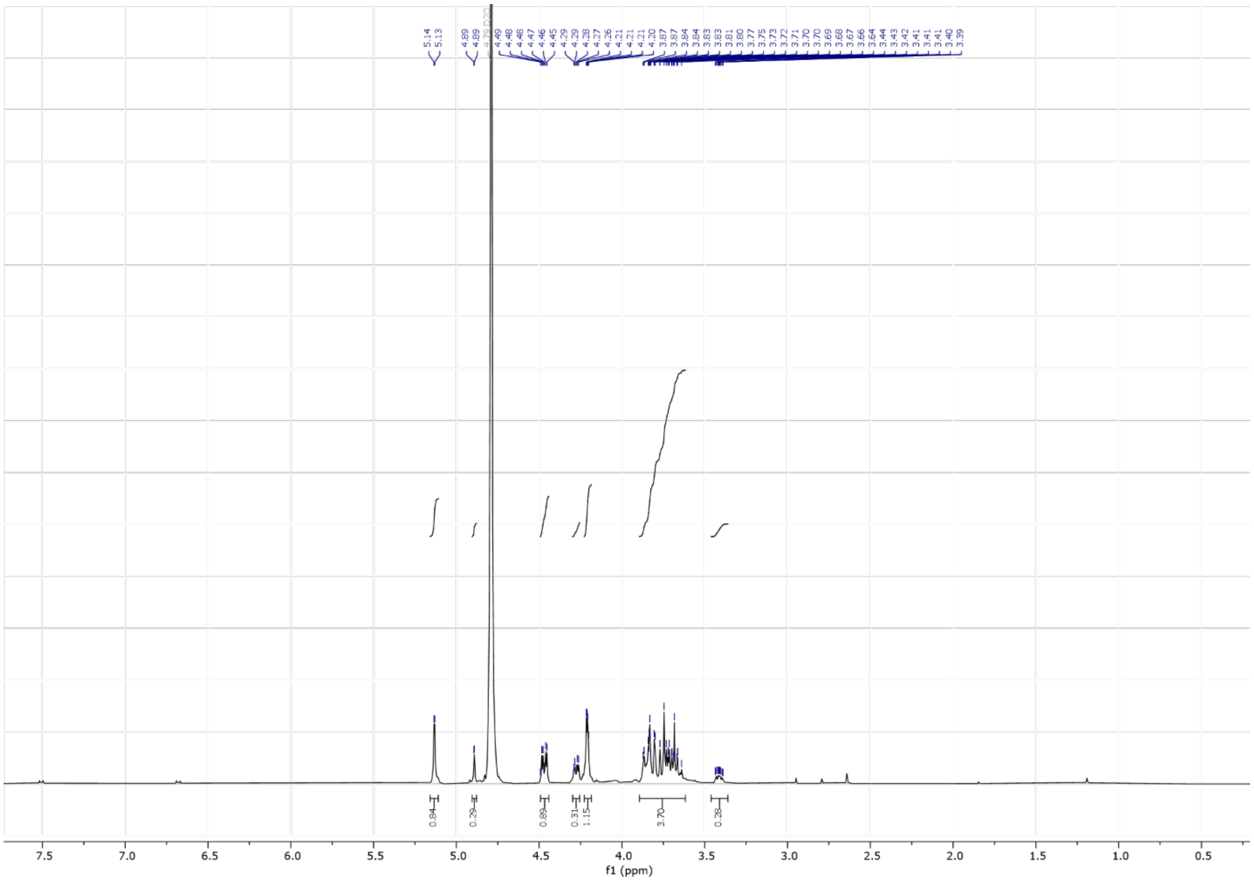

^{1}H NMR

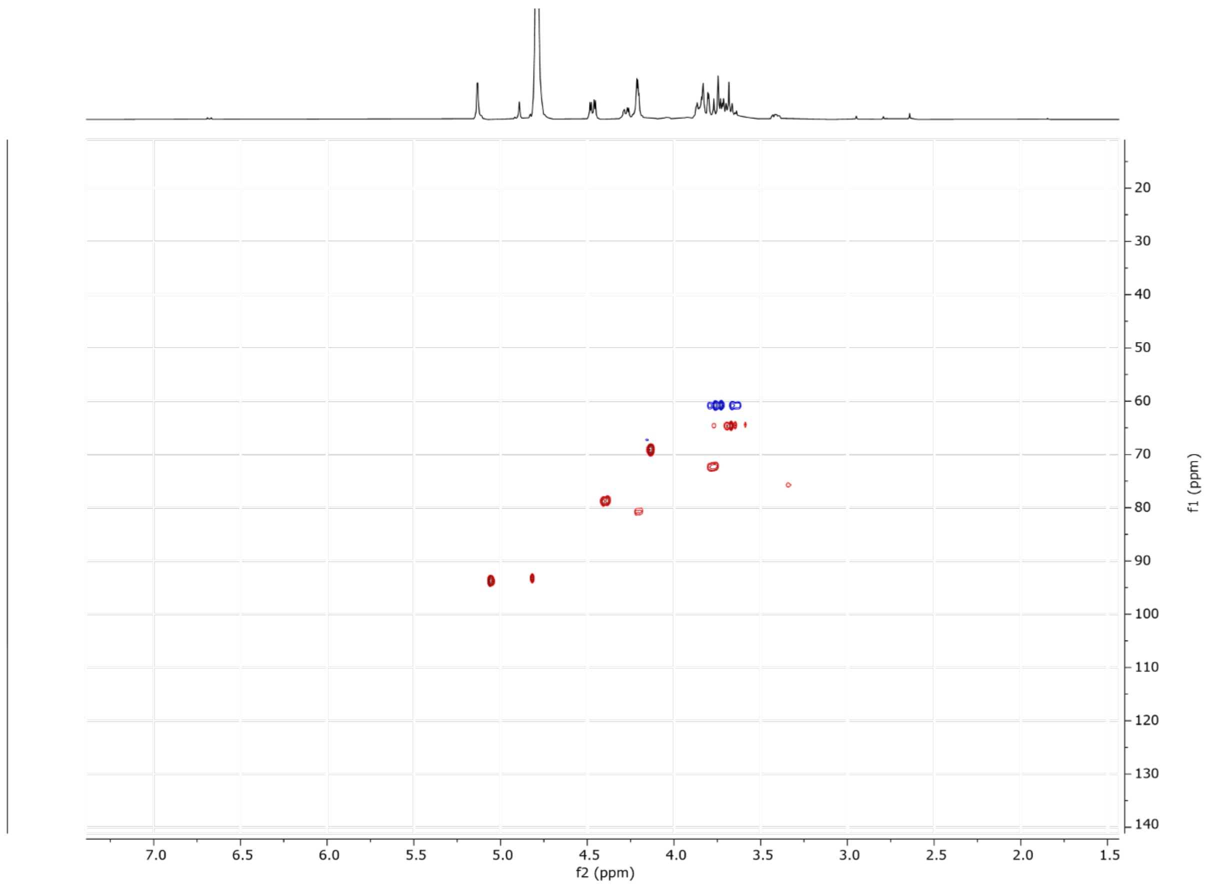

^{1}H-^{13}C HSQC NMR

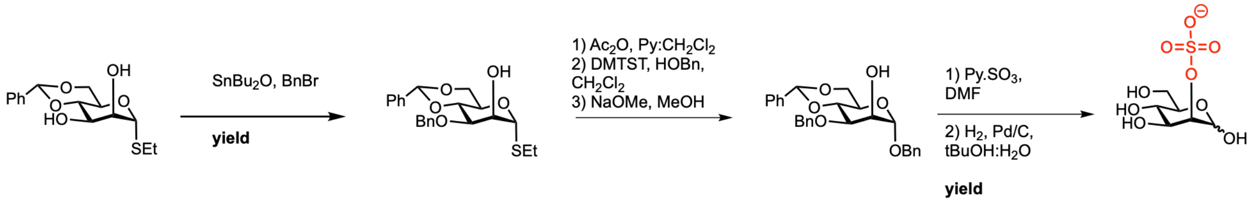

Scheme X. Synthesis of 2-*O*-sulfated mannose.

Ethyl 4,6-*O*-benzylidene-3-*O*-benzyl-1-thio-α-D-mannopyranoside (11)

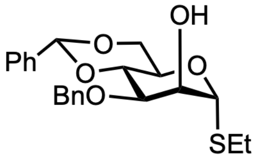

Ethyl 4,6-*O*-benzylidene-1-thio-α-D-mannopyranoside (1.5 g, 4.19 mmol) was dissolved in anhydrous toluene (0.1M, 65 mL), *n*Bu_2SnO (1.25 g, 5.03 mmol, 1.2 eq.) was added, and the reaction was left to reflux overnight. Subsequently, the reaction was brought to 40 °C. Thereafter, benzyl bromide (2.8 g, 12.6 mmol, 3 eq.) and TBAI (0.3 g, 0.83 mmol, 0.2 eq.) was added. Once the reaction was complete, the solution was concentrated under vacuum and purified by flash chromatography (SiO_2, hexane/EtOAc) to yield **11** (1.1 g, 2.93 mmol, 70%).

^{1}H NMR (400 MHz, $CDCl_3$) δ 7.51 – 7.39 (m, 2H), 7.38 – 7.16 (m, 9H), 5.54 (s, 1H), 5.29 (s, 1H), 4.78 (d, *J* = 11.7 Hz, 1H), 4.62 (d, *J* = 11.9 Hz, 1H), 4.22 – 3.95 (m, 4H), 3.86 – 3.71 (m, 2H), 2.64 – 2.46 (m, 3H), 1.29 – 1.14 (m, 3H). **^{13}C NMR** (101 MHz, $CDCl_3$) δ 137.9, 137.6, 134.6, 129.9, 129.1, 129.1, 128.6, 128.4, 128.1, 128.0, 126.2, 101.7, 84.2, 79.2, 76.0, 73.2, 71.5, 68.8, 63.9, 25.0, 15.0. **HRMS** QTOF-MS: calcd. $C_{22}H_{27}O_5S$ for $[M+H]^+$ 403.1579, found 403.1585.

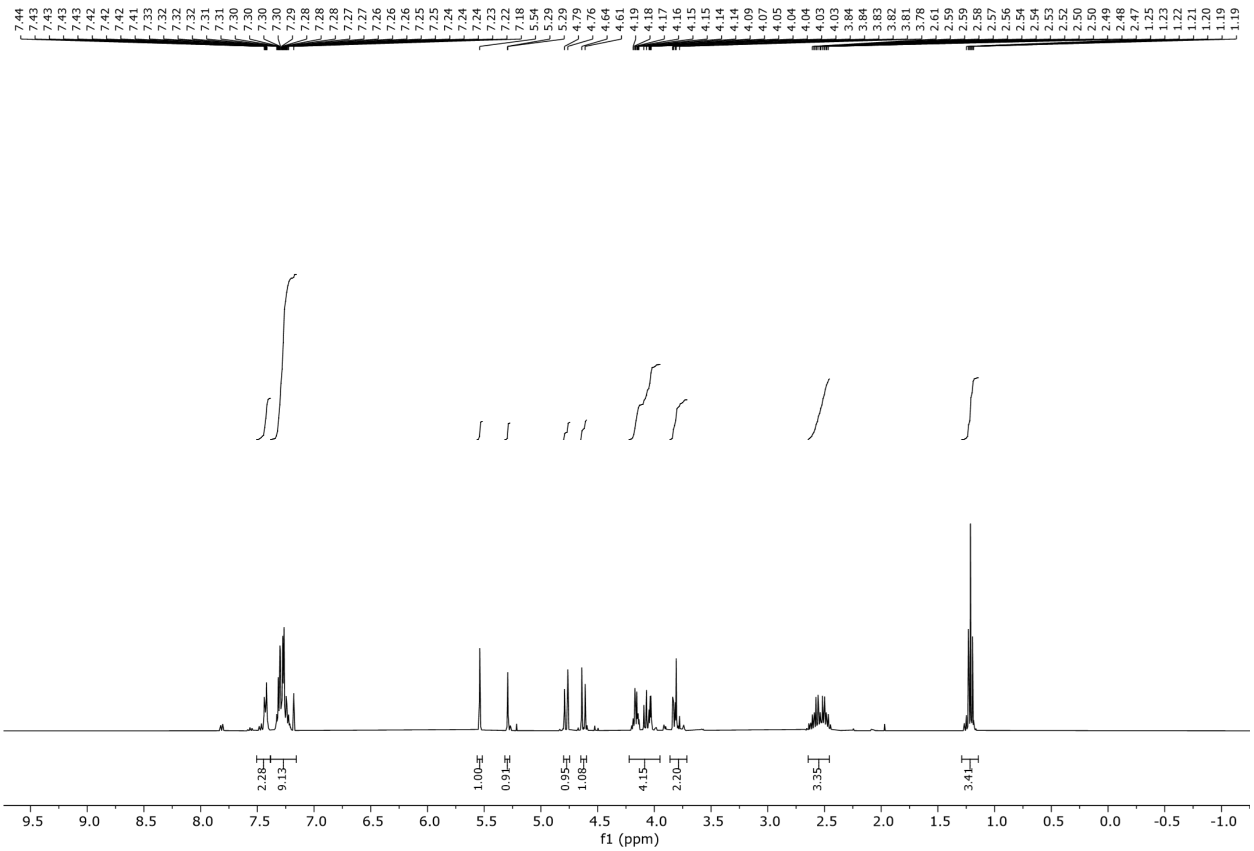

^{1}H NMR

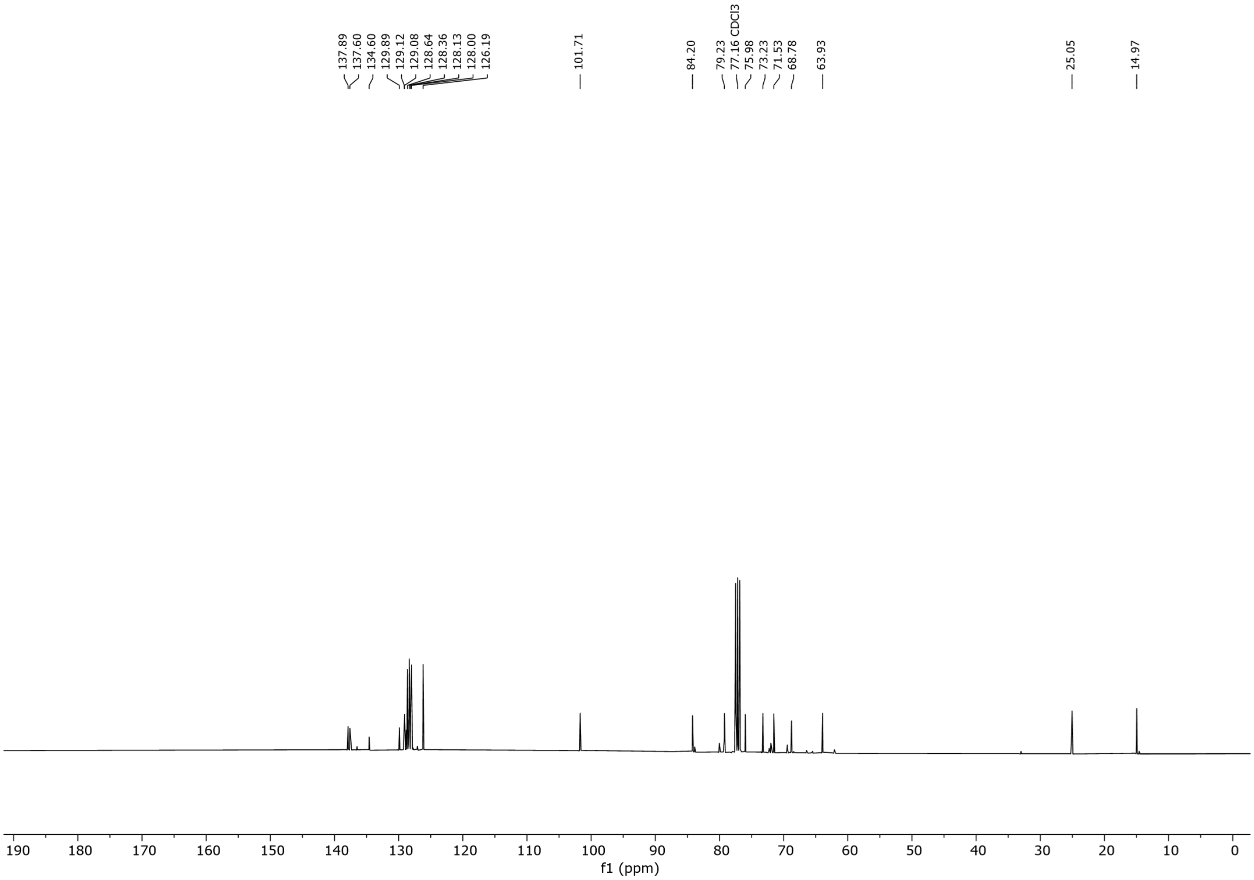
137.89
137.60
134.60
129.89
129.12
129.08
128.64
128.36
128.13
128.00
126.19
101.71
84.20
79.23
77.16 CDCl3
75.98
73.23
71.53
68.78
63.93
25.05
14.97
190 180 170 160 150 140 130 120 110 100 90 80 70 60 50 40 30 20 10 0
f1 (ppm)

^{13}C NMR

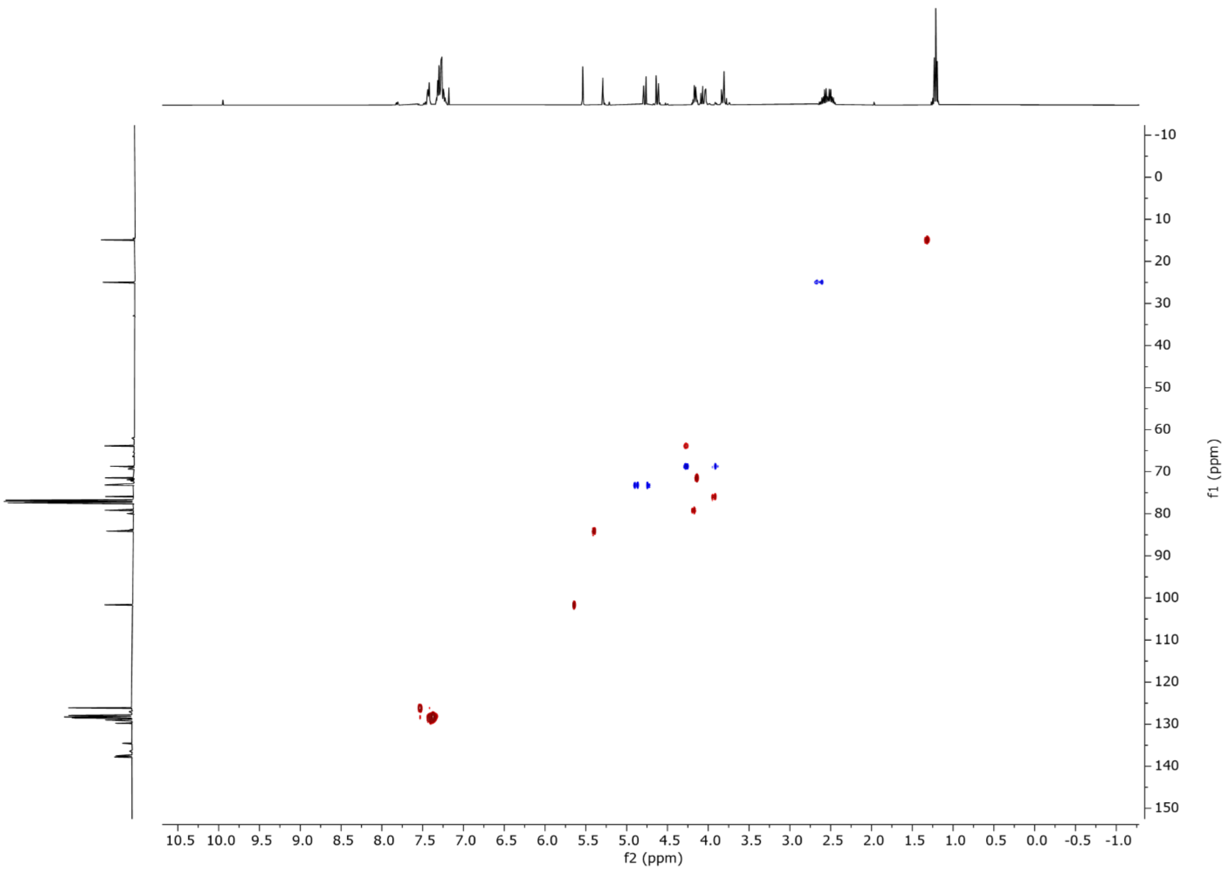

^{1}H-^{13}C HSQC NMR

2-*O*-sulfonate-D-mannopyranose (12)

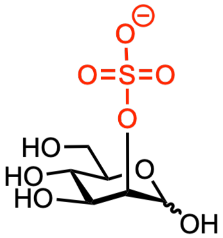

Ethyl 4,6-*O*-benzylidene-3-*O*-benzyl-1-thio-α-D-mannopyranoside (1 g, 2.48 mmol) was dissolved in CH_2Cl_2 (0.1 M, 25 mL) at 0 °C. Subsequently, acetic anhydride (0.4 mL, 4.96 mmol, 2 eq.), pyridine (1 mL, 12.4 mmol, 5 eq.) and 4-dimethylaminopyridine (60 mg, 0.49 mmol, 0.2 eq.) were added. The mixture was stirred at room temperature under argon atmosphere. Once complete, as indicated by TLC analysis (3 h), the reaction was quenched with citric acid (10% w/v) and extracted. The organic layer was washed with saturated aqueous $NaHCO_3$, brine, dried over Na_2SO_4, and concentrated. The residue was purified by flash chromatography (SiO_2, Hexane/Ethyl acetate) to give intermediate ethyl 2-*O*-acetyl-4,6-*O*-benzylidene-3-*O*-benzyl-1-thio-α-D-mannopyranoside. The intermediate was then dissolved in anhydrous CH_2Cl_2 (0.1M, 25 mL) and benzyl alcohol (0.7 mL, 7.44 mmol, 3 eq.) and 4Å molecular sieves were added. After 30 minutes, the mixture was cooled to 0 °C and DMTST (1.2 g, 4.96 mmol, 2 eq.) was added. Once TLC analysis indicated the reaction had reached completion it was quenched with triethylamine (3 mL), diluted with DCM, and filtered through a plug of Celite®. The solution was washed with saturated aqueous $NaHCO_3$, brine, and dried over Na_2SO_4 and concentrated. The material was purified by flash chromatography (SiO_2, Hexane/EtOAc) to yield the intermediate benzyl 2-*O*-acetyl-4,6-*O*-benzylidene-3-*O*-benzyl-1-thio-α-D-mannopyranoside. This intermediate was dissolved in CH_2Cl_2 (0.1M, 25 mL) and the pH was raised to pH 8 with a sodium methoxide (approx. 0.2 mL, 0.5M solution). Once complete, the reaction was quenched by the addition of Amberlite™ (Hydrogen foam), filtered, and concentrated under vacuum. Purification by flash chromatography (SiO_2, CH_2Cl_2/methanol). The benzyl 4,6-*O*-benzylidene-3-*O*-benzyl-1-thio-α-D-mannopyranoside (0.1 g, 0.11 mmol) was dissolved in DMF (1 mL) and Py·SO_3 (1 g, mmol, 1.2 eq.) was added and stirred at room temperature. Upon completion of reaction as indicated by TLC (16 h), a premixed solution of Et_3N/MeOH (1/1, v/v, 1.0 mL) was added to quench the reaction and stirring was continued for another 30 mins. The reaction mixture was concentrated and purified by flash chromatography (SiO_2, MeOH/ CH_2Cl_2) to give the intermediate sulfated compound. This intermediate was then dissolved in THF: *t*BuOH: H_2O (4 mL, 50:20:30) and 5% Pd-C (200 mg) was added. The reaction was stirred under H_2 atmosphere for 16 h. The reaction was filtered through a pad of celite and washed with *t*BuOH and H_2O. The filtrates were concentrated *in vacuum*, and dissolved in 2.0 mL of water for RP-HPLC purification (Method 6) to yield **12** (14 mg, 0.05 mmol, 2%, over 6 steps).

^{1}H NMR (400 MHz, D_2O) δ 5.39 (s, 1H), 4.47 – 4.43 (m, 1H), 3.91 (dd, *J* = 9.4, 3.9 Hz, 1H), 3.84 – 3.66 (m, 3H), 3.59 (t, *J* = 9.6 Hz, 1H). **^{13}C NMR** (101 MHz, D_2O) δ 91.6, 77.7, 72.2, 68.7, 66.6, 60.6. **HRMS** QTOF-MS: calcd. $C_6H_{11}O_9S$ for $[M-H]^-$ 259.0129, found 259.0134.

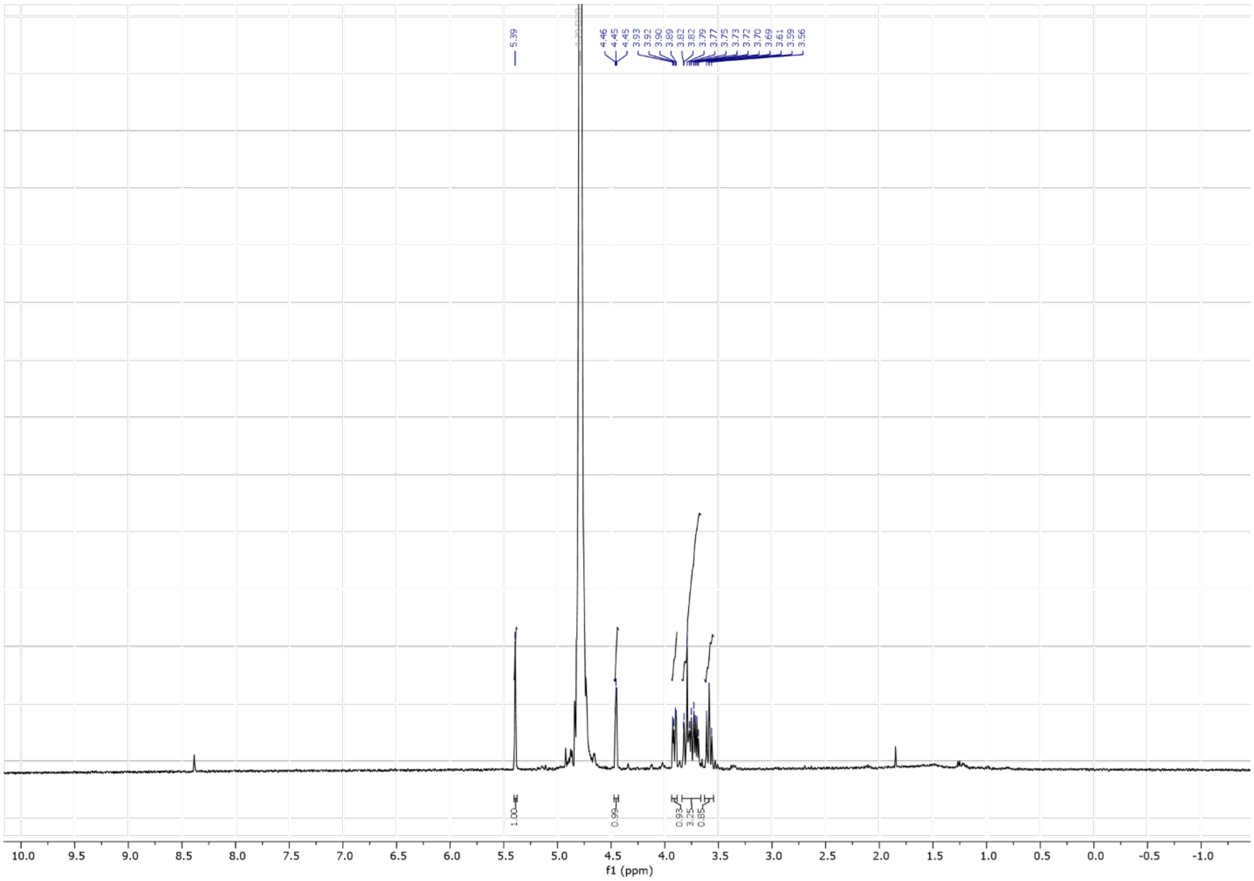
5.39
4.46
4.45
4.45
3.93
3.92
3.90
3.89
3.82
3.82
3.79
3.77
3.75
3.73
3.72
3.70
3.69
3.61
3.59
3.56
1.00
0.99
0.93
3.25
0.85
10.0
9.5
9.0
8.5
8.0
7.5
7.0
6.5
6.0
5.5
5.0
4.5
4.0
3.5
3.0
2.5
2.0
1.5
1.0
0.5
0.0
-0.5
-1.0
f1 (ppm)

^{1}H NMR

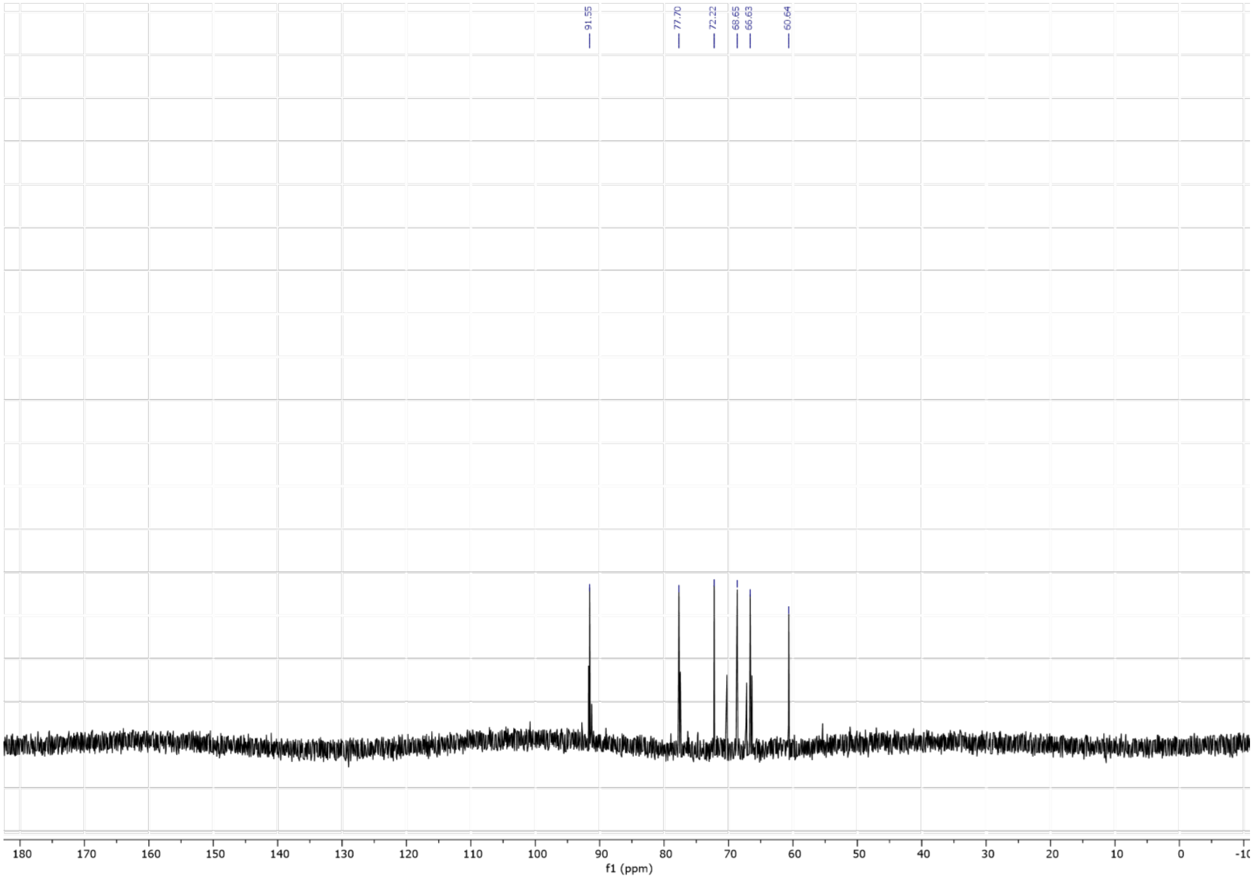

91.55
77.70
72.22
68.65
66.63
60.64
180 170 160 150 140 130 120 110 100 90 80 70 60 50 40 30 20 10 0
f1 (ppm)

^{13}C NMR

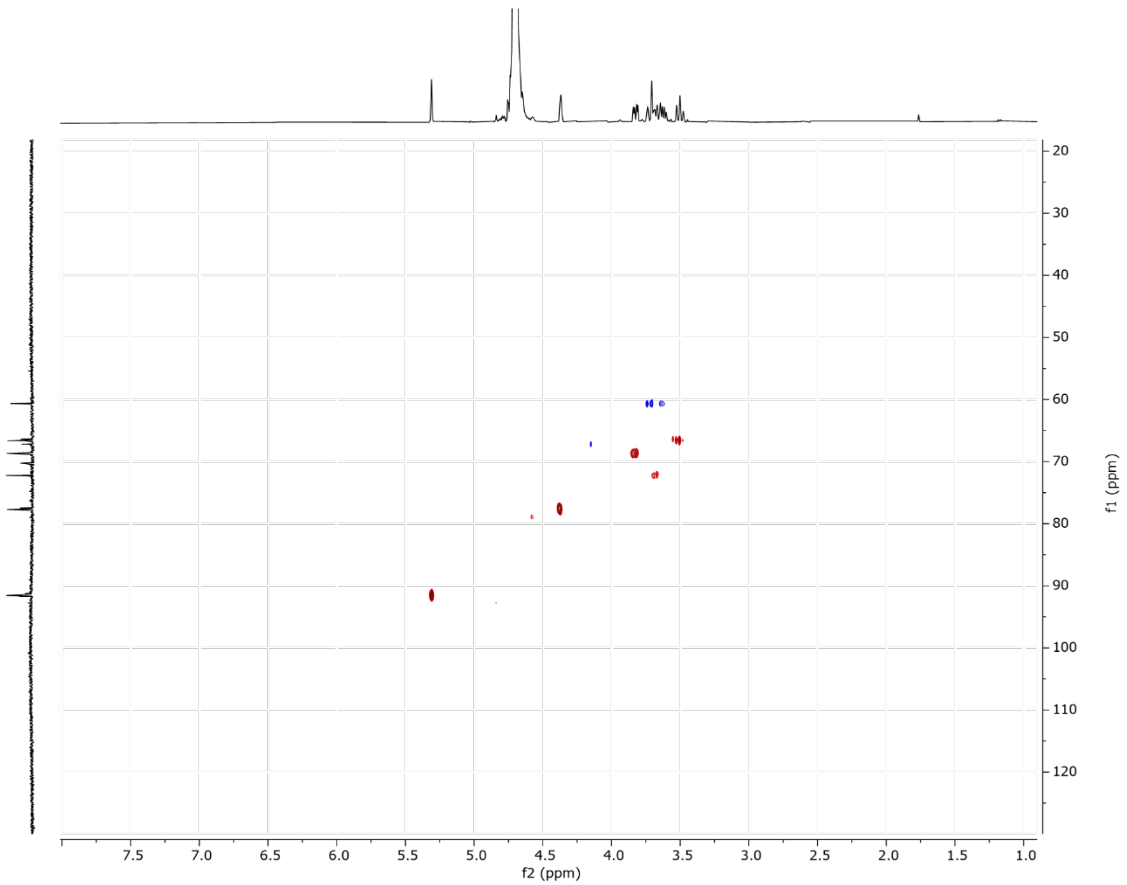

^{1}H-^{13}C HSQC

6-*O*-sulfonate-α-D-mannopyranosyl-(1→3)-6-*O*-sulfonate-α-D-mannopyranosyl-(1→3)-6-*O*-sulfonate-D-mannopyranosyl-(1→3)-6-*O*-sulfonate-α-D-mannopyranosyl-(1→3)-6-*O*-sulfonate-α-D- mannopyranosyl-(1→3)-6-*O*-sulfonate-α-D-mannopyranose (2)

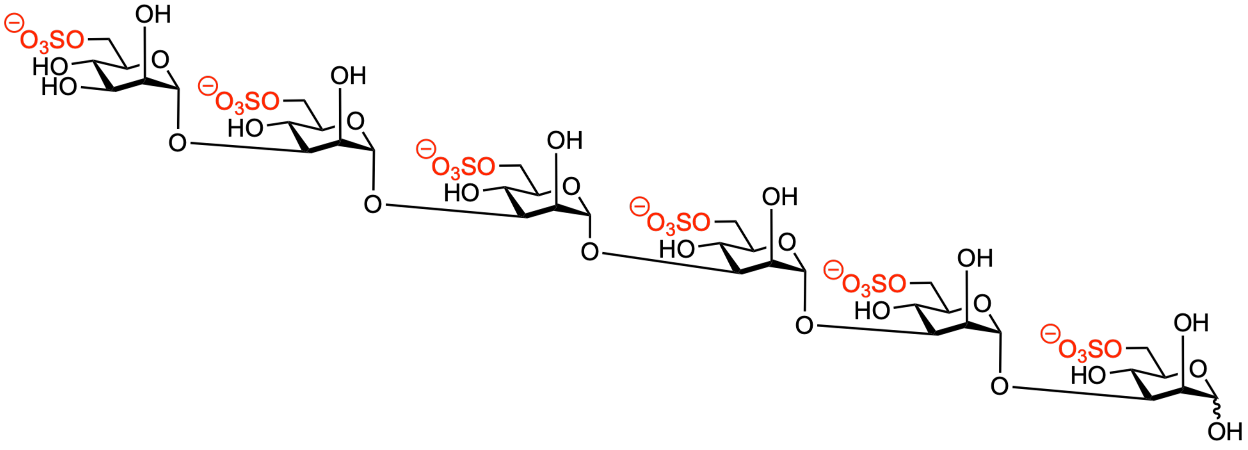

		Modules	Notes
**1. AGA**			L2
	**1**	a, b (x2), c, e	x6
		d	x2
	**Step**	**Module**	**Notes**
**2. Solid-phase synthesis**	Sulfation	g	
	Methanolysis	f	72h
	photocleavage	h	
	**Step**	**Module**	**Notes**
**3. Solution-phase**	Hydrogenolysis	i2	
	Purification	Method 6	

The desired fractions were then collected and lyophilized to yield 0.7 mg (2.3%). **^{1}H NMR** (400 MHz, D_2O) δ 5.10 (d, *J* = 1.8 Hz, 1H, H-1), 5.08 (d, *J* = 1.7 Hz, 1H, H-1), 5.07 – 5.05 (m, 3H, H-1), 4.89 (s, 0H, H-1), 4.36 – 4.27 (m, 6H, H-6, H-2), 4.26 – 4.13 (m, 6H), 4.11 – 3.94 (m, 5H, H-3), 3.91 – 3.82 (m, 2H, C-3), 3.82 – 3.71 (m, 4H), 3.71 – 3.56 (m, 1H). **^{13}C NMR** (101 MHz, D_2O) δ 102.6 (C-1), 94.0 (C-1), 79.0 (C-3), 71.3, 69.4, 67.4 (C-6), 65.5. **HRMS** QTOF-MS: calcd. $C_{36}H_{59}O_{49}S_6$ for $[M-3H]^{-3}$ 489.0155, found 489.0159.

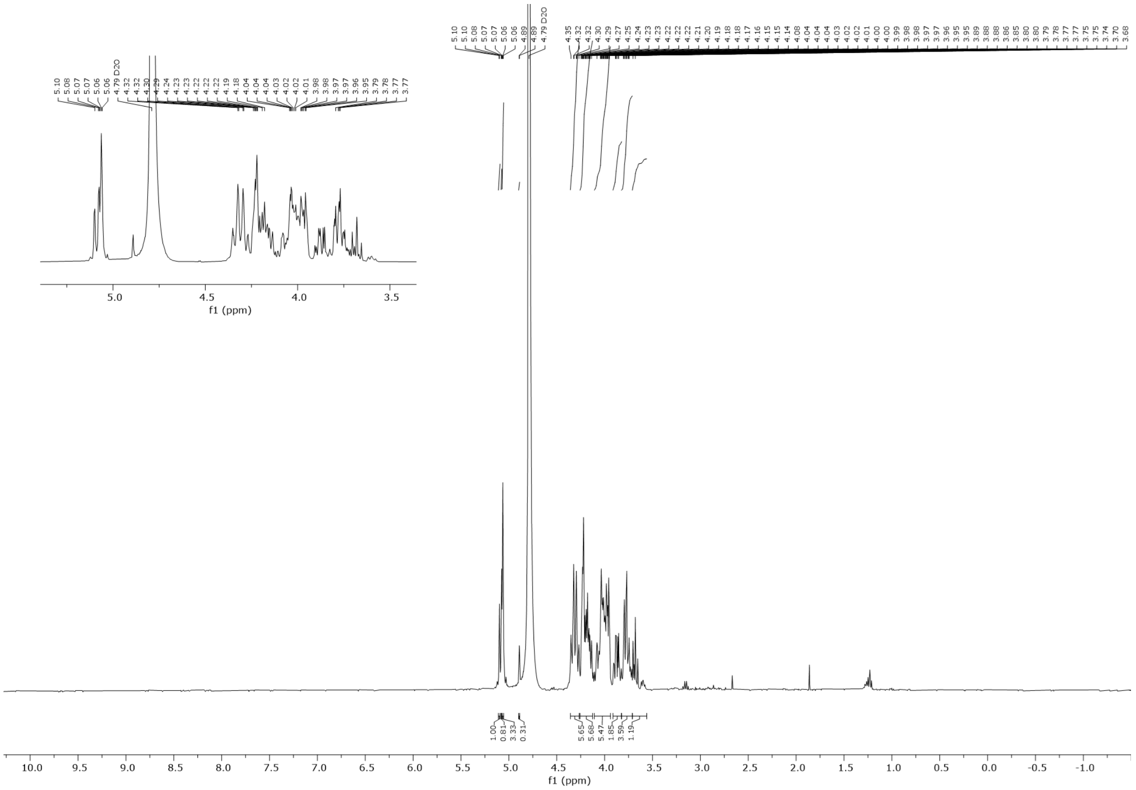

^{1}H NMR

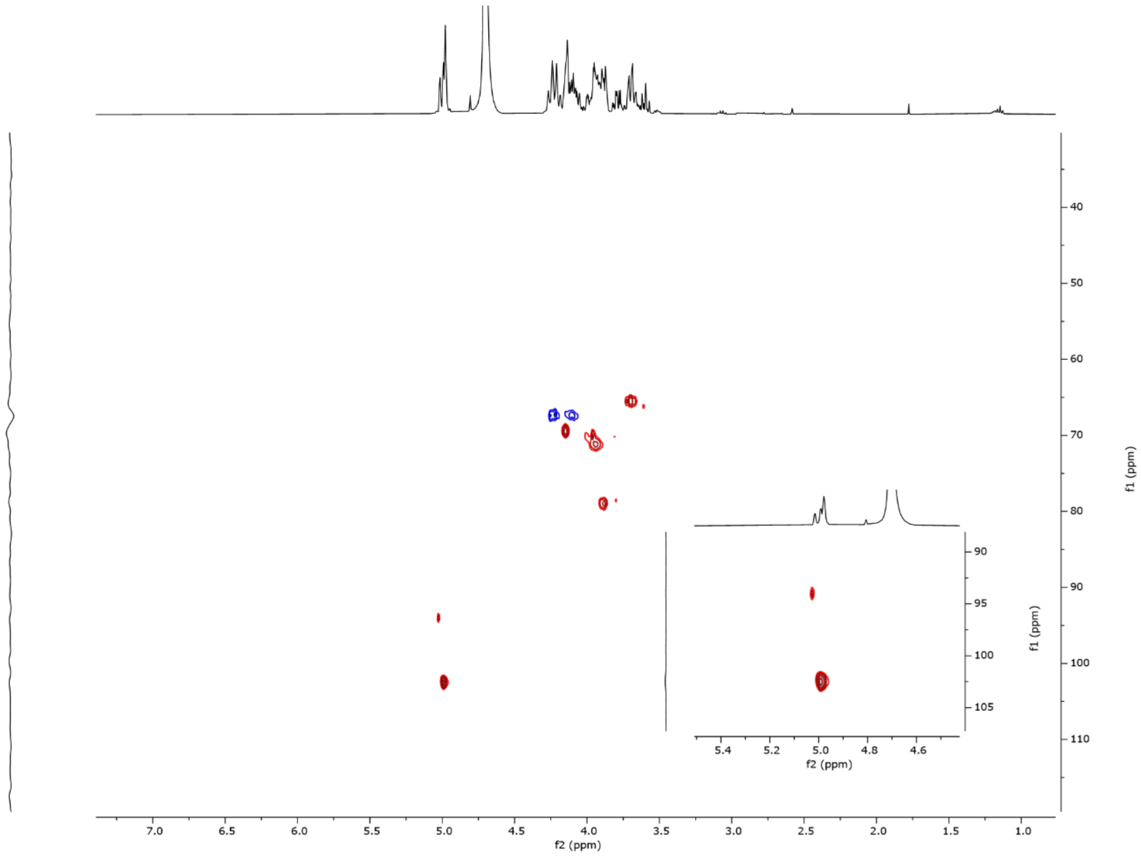

^{1}H-^{13}C HSQC NMR

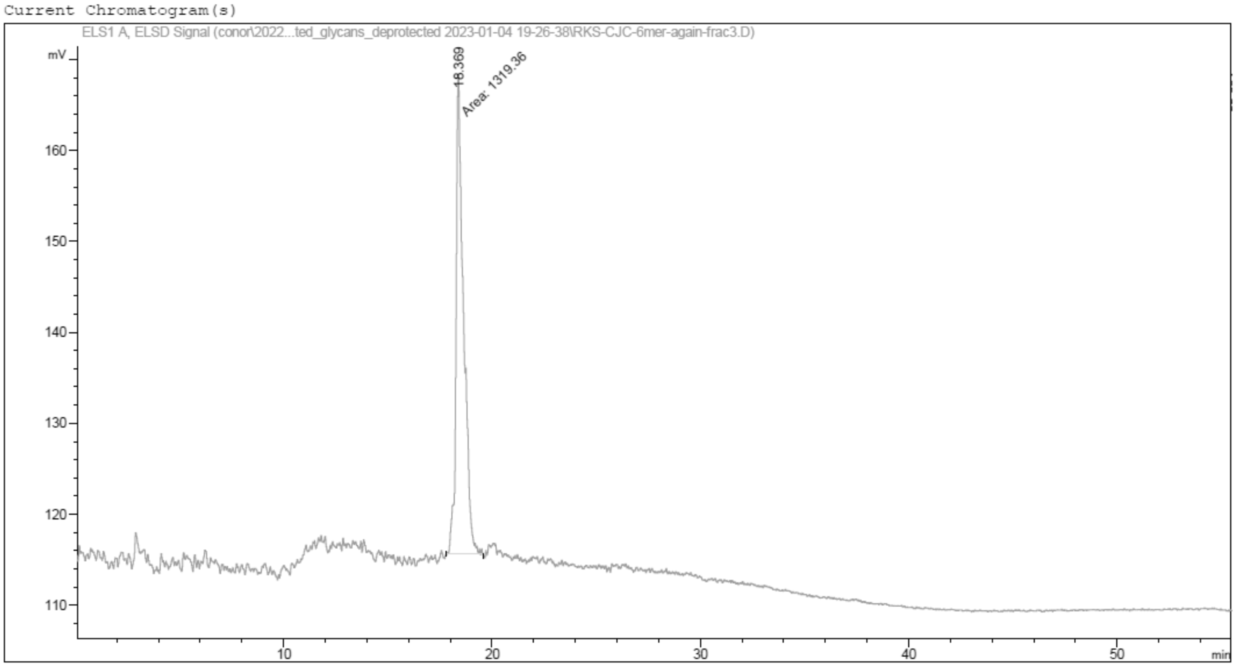

RP HPLC Trace. Hypercarb**.**

α-D-mannopyranosyl-(1→3)-α-D-mannopyranosyl-(1→3)-D-L- mannopyranosyl-(1→3)-α-D-mannopyranosyl-(1→3)-α-D- mannopyranosyl-(1→3)-α-D-mannopyranose (3)

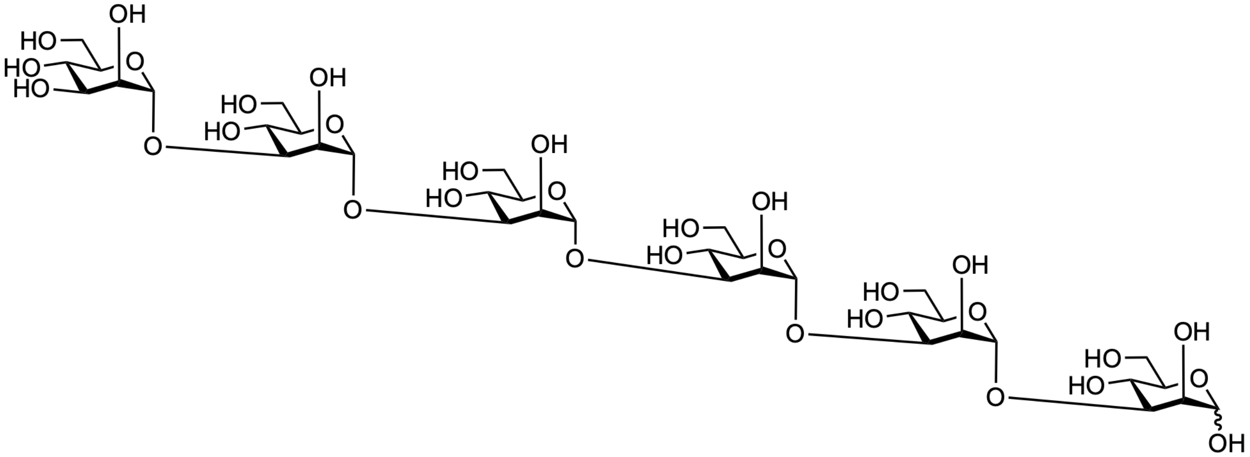

		Modules	Notes
**1. AGA**			L2
	**1**	a, b (x2), c, e	x6
		d	x2
	**Step**	**Module**	**Notes**
**2. Solid-phase synthesis**	Methanolysis	f	
	photocleavage	h	
	**Step**	**Module**	**Notes**
**3. Solution-phase**	Hydrogenolysis	i1	
	Purification	Method 5	

The desired fractions were then collected and lyophilized to yield 0.9 mg (4.5%). **^{1}H NMR** (600 MHz, D_2O) δ 5.15 (d, *J* = 1.9 Hz, 1H, H-1), 5.14 (d, *J* = 1.9 Hz, 1H, H-1), 5.13 – 5.11 (m, 5H, H-1), 4.91 (sf, 0H), 4.26 – 4.22 (m, 6H, H-2), 4.10 – 4.05 (m, 4H), 4.05 – 4.00 (m, 6H, H-3), 3.95 – 3.87 (m, 12H), 3.87 – 3.81 (m, 8H), 3.81 – 3.72 (m, 19H), 3.66 (m, 7H). **^{13}C NMR** (151 MHz, D_2O) δ 102.2 (C-1), 93.9 (C-1), 78.2 (C-3), 73.5, 70.3, 70.0, 69.7 (C-2), 66.1, 61.0 (C-6). **HRMS** QTOF-MS: calcd. $C_{36}H_{62}NaO_{31}$ for [M+Na]$^{-}$ 1013.3173, found 1013.3179.

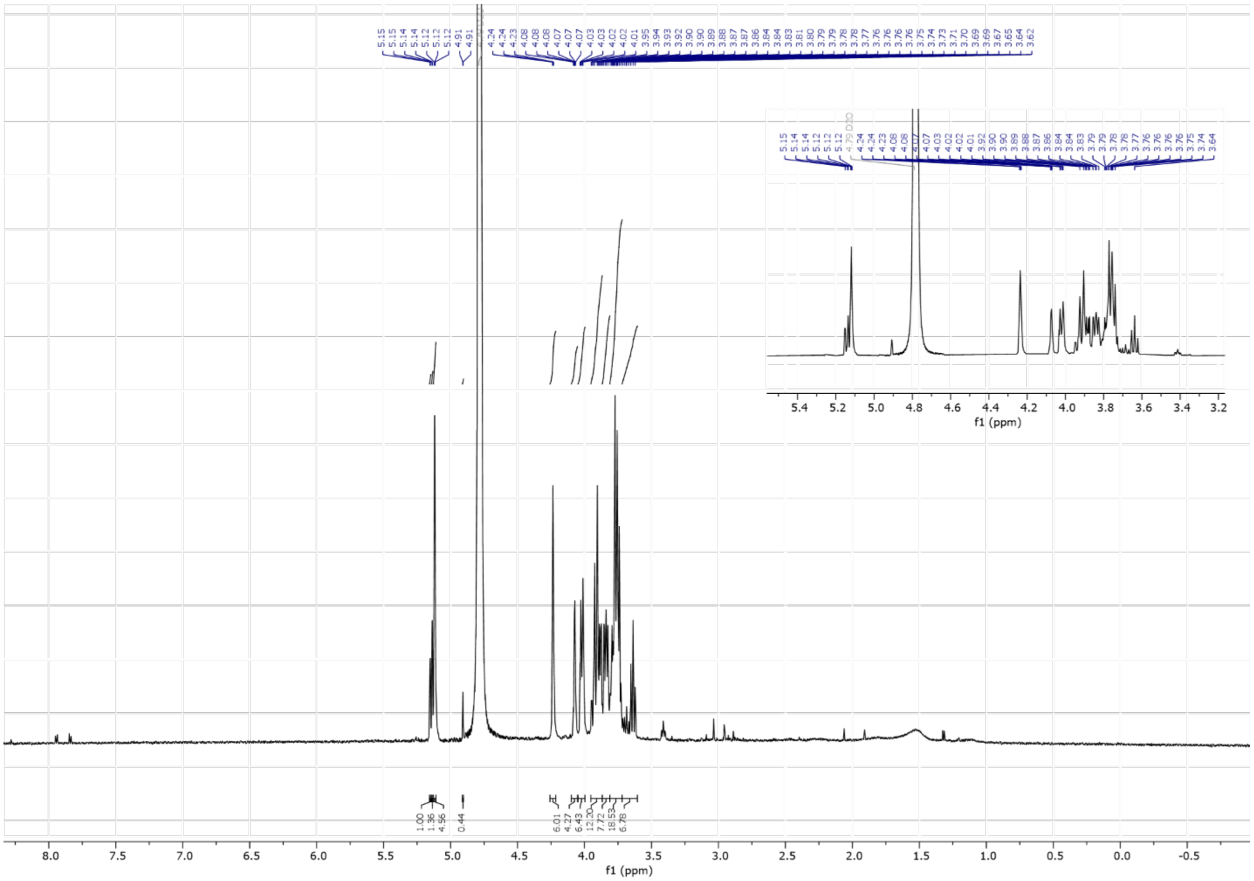

^{1}H NMR

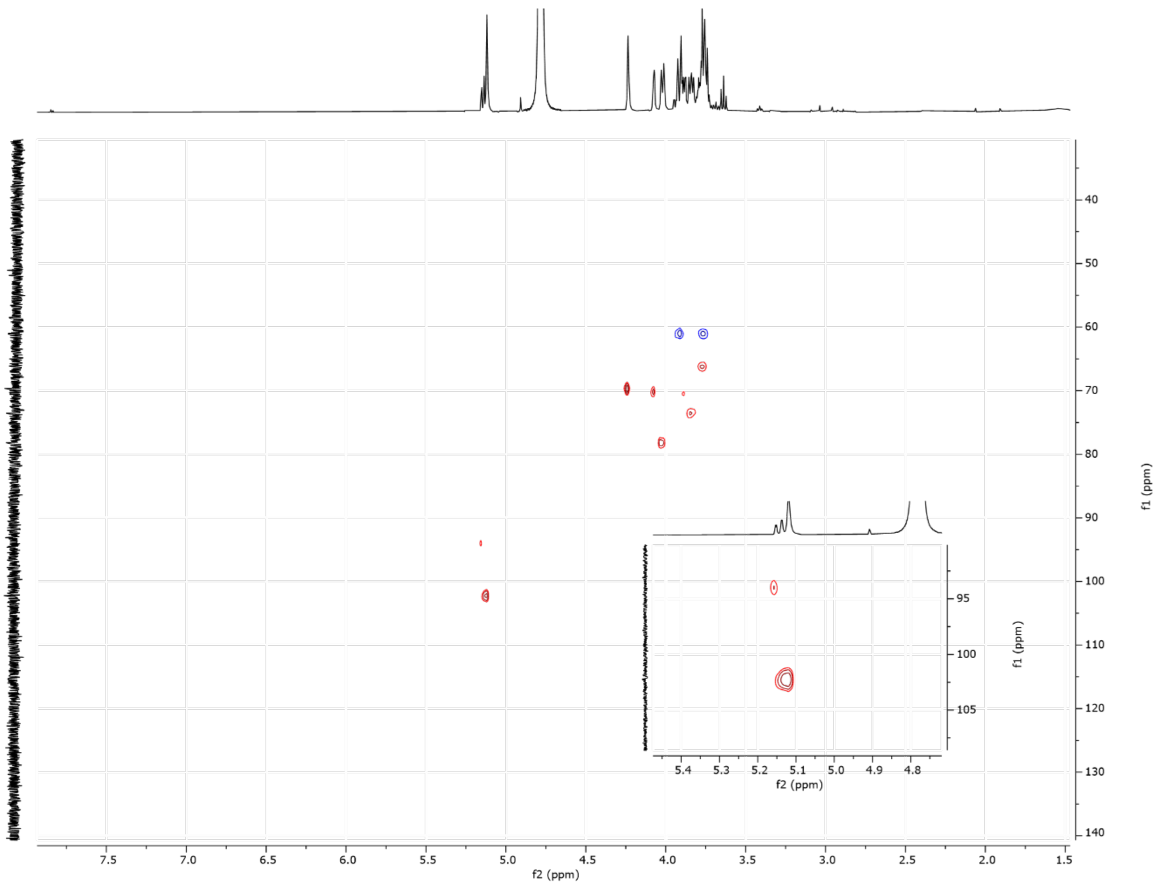

^{1}H-^{13}C HSQC

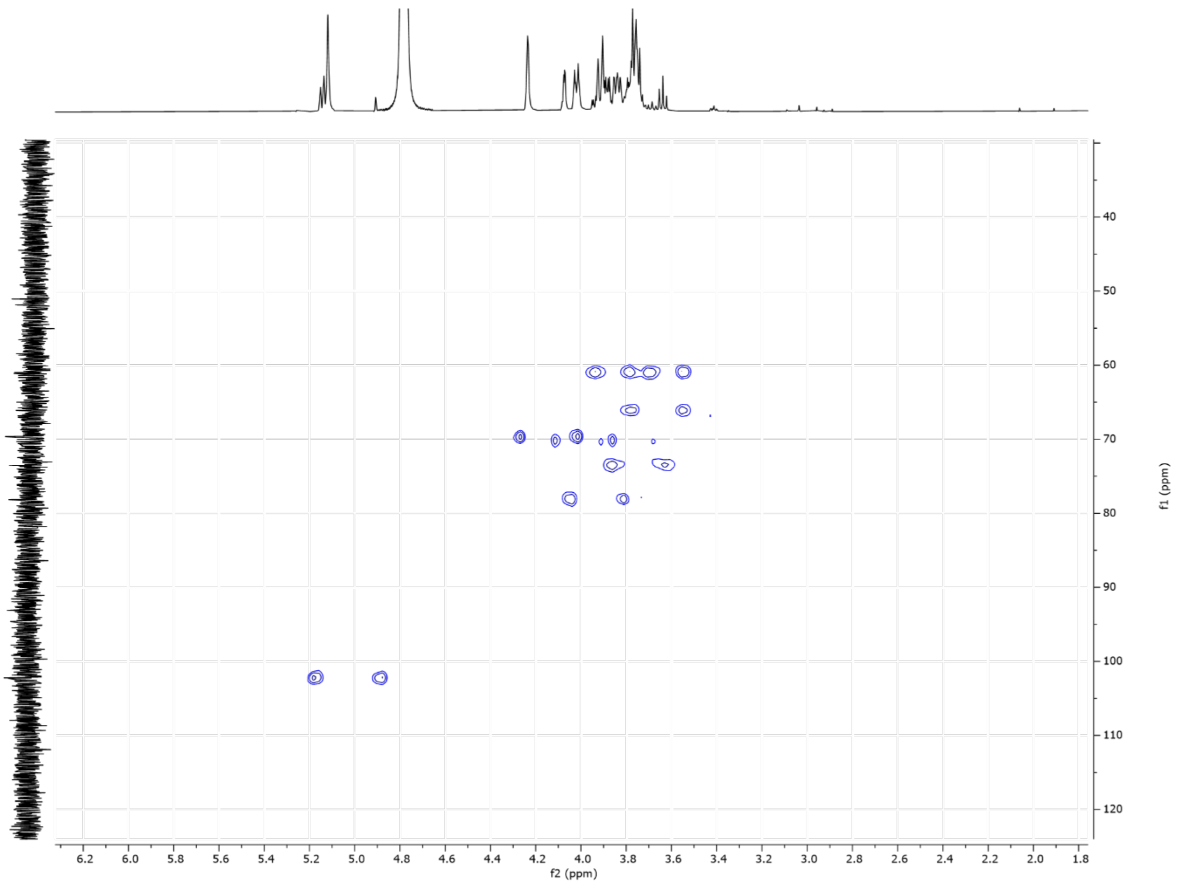

^1H-^{13}C HSCQC (coupled)

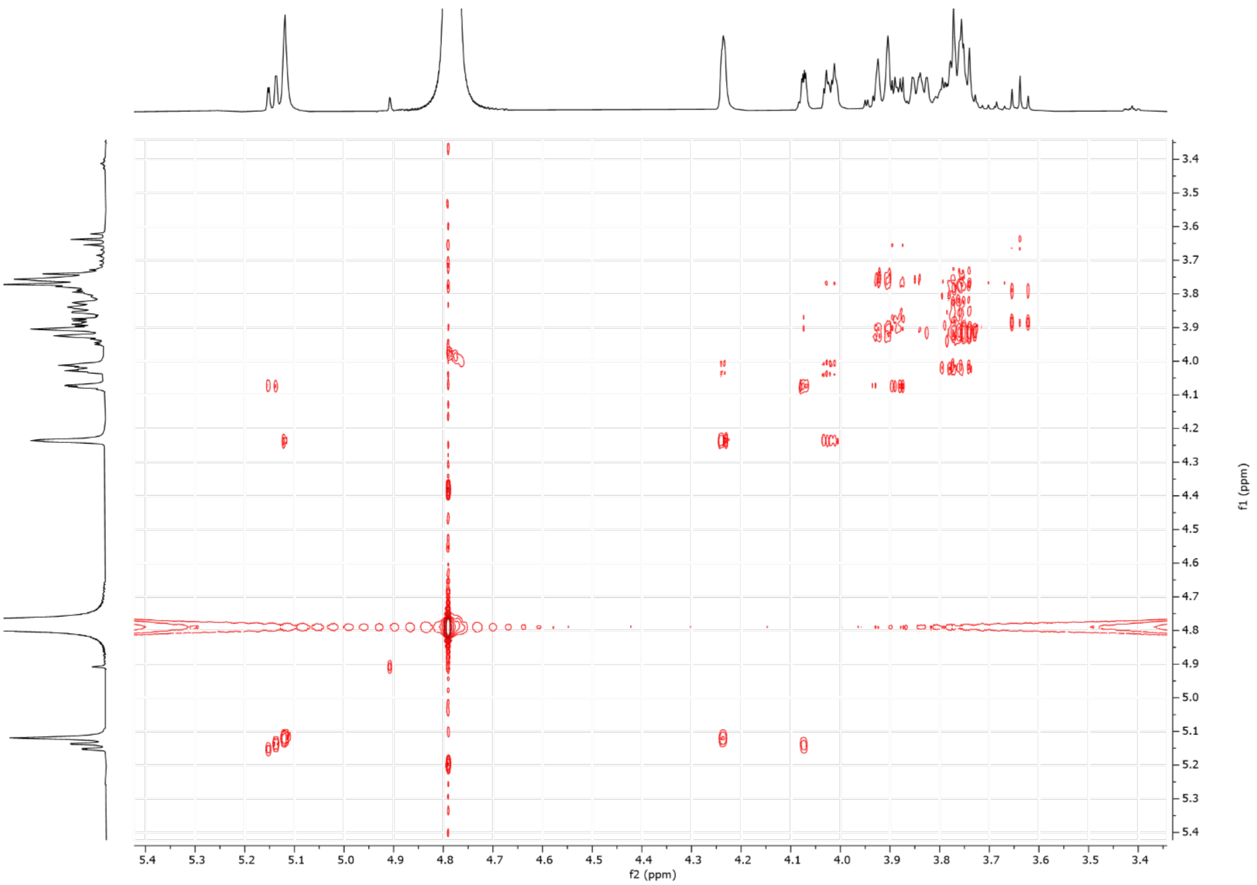

^{1}H-^{1}H COSY NMR

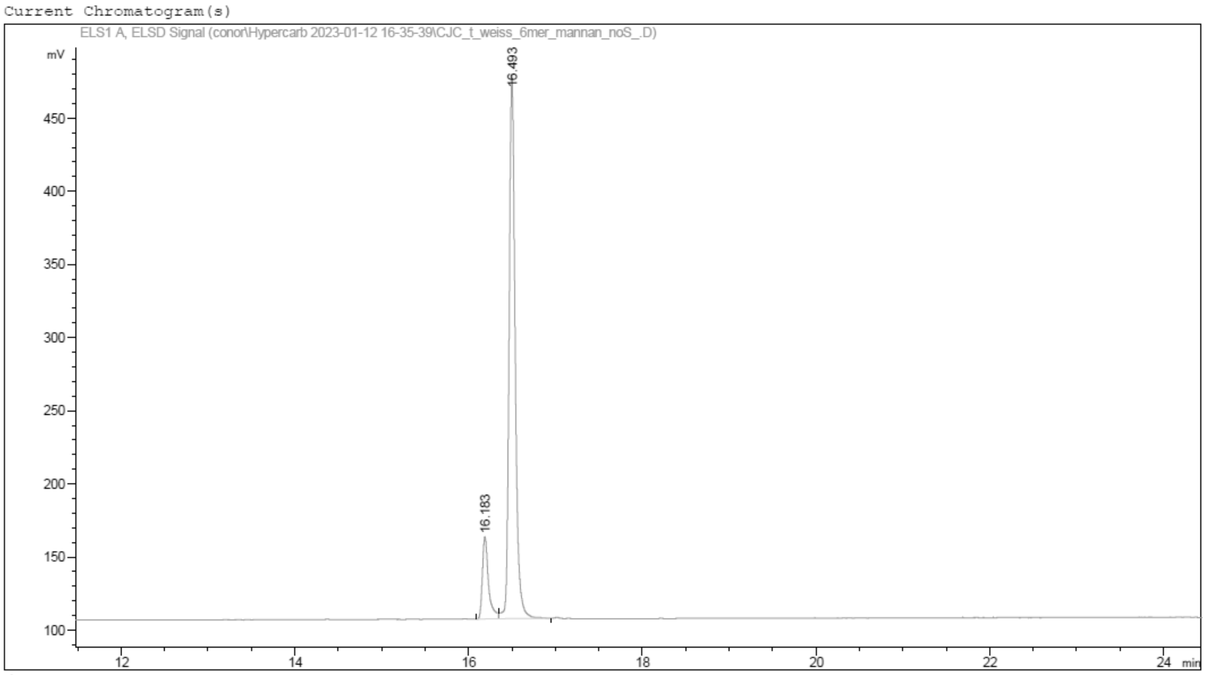

RP HPLC Trace. Hypercarb.
